# Curved π-conjugated corannulene dimer diradicaloids†
†Electronic supplementary information (ESI) available: Synthetic procedures and characterization data of all new compounds; details for all physical characterization and theoretical calculations; and additional spectroscopic data. CCDC 1570628, 1819178 and 1819179. For ESI and crystallographic data in CIF or other electronic format see DOI: 10.1039/c8sc01388h


**DOI:** 10.1039/c8sc01388h

**Published:** 2018-05-16

**Authors:** Qing Wang, Pan Hu, Takayuki Tanaka, Tullimilli Y. Gopalakrishna, Tun Seng Herng, Hoa Phan, Wangdong Zeng, Jun Ding, Atsuhiro Osuka, Chunyan Chi, Jay Siegel, Jishan Wu

**Affiliations:** a Department of Chemistry , National University of Singapore , 3 Science Drive 3 , 117543 , Singapore . Email: chmwuj@nus.edu.sg ; Email: chmcc@nus.edu.sg; b Department of Chemistry , Graduate School of Science , Kyoto University , Sakyo-ku , Kyoto 606-8502 , Japan; c Department of Materials Science and Engineering , National University of Singapore , 119260 , Singapore; d Health Science Platform , Tianjin University , 92 Weijin Road, Nankai District , Tianjin , 300072 , P. R. China . Email: dean_spst@tju.edu.cn

## Abstract

So far, most reported open-shell singlet diradicaloids are based on planar π-conjugated molecules. Herein, we report the bridged corannulene dimer diradicaloids, **Cor-D1** and **Cor-D2**, both showing a three-dimensional curved π-conjugated structure. **Cor-D1** has a small diradical character (*y*_0_ = 5.4%) and behaves more like a closed-shell quinoidal compound at room temperature, while **Cor-D2** is a typical open-shell diradicaloid with a larger diradical character (*y*_0_ = 16.9%). Both compounds exhibited paramagnetic activity at elevated temperatures, with a singlet–triplet energy gap (Δ*E*_S–T_) of –8.4 and –3.0 kcal mol^–1^, respectively. X-ray crystallographic analysis revealed that both molecules have a dumbbell-shaped geometry, with the two terminal corannulene bowls bent to opposite directions. The spin is largely delocalized onto the two bowls in **Cor-D2** and there are multiple [CH···π] interactions between the neighboring bowls. Chemical oxidation/reduction to their respective dications/dianions results in global aromaticity with [4*n* + 2] π-electrons delocalized through the periphery of the whole framework.

## Abstract 2

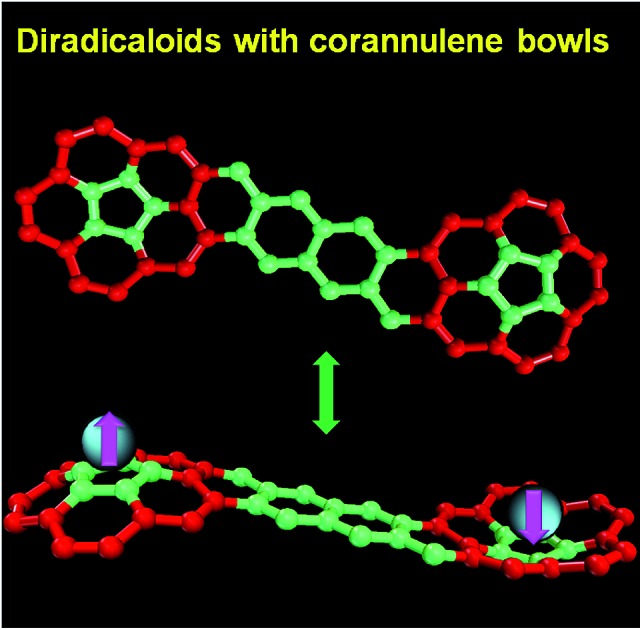
Curved π-conjugated diradicaloids based on bridged corannulene dimers were synthesized, showing 3D spin delocalization and global aromaticity for their dianions.

## Introduction

**Fig. 1:**
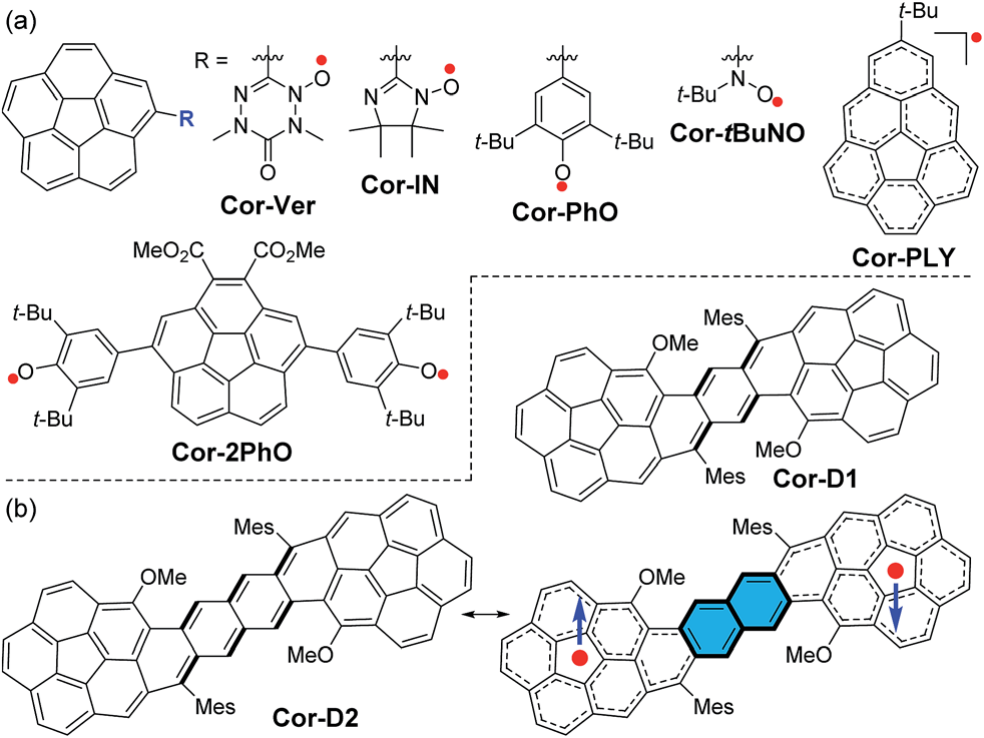
(a) Structures of neutral radical-substituted corannulenes, phenalenyl-fused corannulene, and corannulene-bridged phenoxyl diradical. (b) Structures of the new *p*-QDM and 2,6-NQDM bridged corannulene dimer diradicaloids **Cor-D1** and **Cor-D2**. The closed-shell quinoidal and open-shell diradical resonance forms are shown for **Cor-D2**. Mes: mesityl.

Recently there have been increasing research interests on open-shell singlet diradicaloids due to their unique magnetic activity and potential applications in organic electronics, non-linear optics and spintronics.^1^ So far, most reported open-shell singlet diradicaloids are based on planar π-conjugated molecules or their oligomers, and there are very rare examples based on non-planar systems.^2^ Our particular interest here is to exploit stable diradicaloids based on curved π-conjugated molecules, which may exhibit unique three-dimensional (3D) spin distribution nature and intra- and intermolecular magnetic exchange interactions. Bowl-shaped corannulene is a very good candidate given its curved structure. It was reported that the radical anions of corannulene and its derivatives showed effective spin delocalization throughout the π-conjugated framework but they were still highly reactive and some of them tended to form intermolecular C–C σ-bond linked dimer or polymer.^3^ On the other hand, stable neutral radicals-substituted corannulenes such as **Cor-Ver** (verzazyl), **Cor-IN** (iminonitroxide), **Cor-PhO** (phenoxyl) and **Cor-*^t^*BuNO** (*tert*-butylnitroxide) (Fig. 1a) were prepared and in all cases, the spin can be partially delocalized to the corannulene bowl.^4^ The phenalenyl-fused corannulene anion was synthesized and fully characterized, but the property of its neutral radical **Cor-PLY** (Fig. 1a) was not reported, presumably due to its poor stability.^5^ The corannulene-bridged phenoxyl diradical **Cor-2PhO** showed significant intramolecular magnetic interaction with a singlet–triplet energy gap (Δ*E*_S–T_ = 2*Jk*_B_^–1^) of –810 K (–1.61 kcal mol^–1^).^6^ Calculations reveal that the unpaired electronic spin in the triplet state is delocalized onto the corannulene skeleton from the phenoxyl moieties. However, so far, corannulene-based radicals with more than one corannulene unit have never been reported. Herein, we report the first two stable π-conjugated corannulene dimer diradicaloids, **Cor-D1** and **Cor-D2**, in which the two corannulene units are annulated with a *para*-quinodimethane (*p*-QDM) and 2,6-naphthoquinodimethane (2,6-NQDM) moiety, respectively (Fig. 1b). The central pro-aromatic quinodimethanes have irresistible wish to become diradicals by recovering aromaticty,1*d* and one can expect a significant contribution of the open-shell diradical form to the ground-state electronic structure. Therefore, they are ideal open-shell singlet diradicaloids with a unique 3D curved π-conjugated structure and the spin is supposed to be largely delocalized onto the two corannulene bowls (Fig. 1b).

## Results and discussion

### Synthesis

**Scheme 1:**
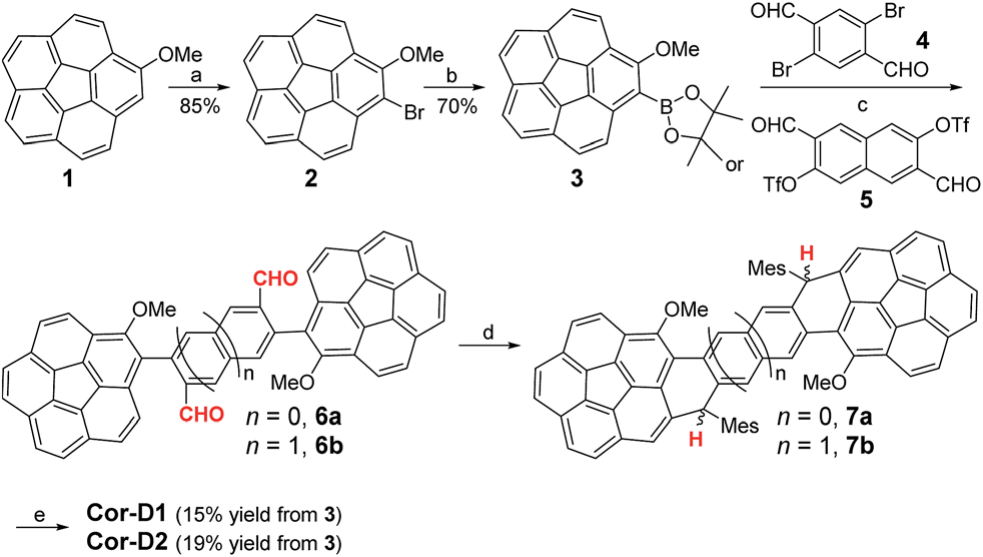
Synthetic routes of **Cor-D1** and **Cor-D2**. *Reagents and conditions*: (a) NBS, *i*-Pr_2_NH, DCM, rt; (b) (i) *n*-BuLi, –78 °C, THF; (ii) 2-isopropoxy-4,4,5,5-tetramethyl-1,3,2-dioxaborolane, –78 °C – rt; (c) Pd(PPh_3_)_4_, K_2_CO_3_, toluene/EtOH/H_2_O (2/1/1), 110 °C; (d) (i) mesitylmagnesium bromide, THF, rt; (ii) BF_3_·OEt_2_, DCM, rt; (e) DDQ, toluene, 80 °C.

The synthesis of π-extended corannulenes is challenging^7^ and our synthesis was based on regio-selective reactions starting from the 1-methoxy-corannulene **1** (ref. 8) (Scheme 1). Mono-bromination of **1** at the *ortho*-position by *N*-bromosuccinimide (NBS) in the presence of diisopropylamine gave the 1-bromo-2-methoxy-corannulene **2** in 85% yield. The bromo-group was then converted into pinacol boronate in **3** by lithiation followed by quenching with 2-isopropoxy-4,4,5,5-tetramethyl-1,3,2-dioxaborolane. Suzuki coupling reaction between **3** and the 2,5-dibromoterephthalaldehyde (**4**) or 3,7-diformylnaphthalene-2,6-diyl bis(trifluoromethanesulfonate) (**5**)^9^ gave the corresponding dialdehyde **6a** or **6b**. Nucleophilic addition of the aldehyde groups in **6a**/**6b** with excessive mesitylmagnesium bromide generated the diol intermediates and subsequent BF_3_·Et_2_O mediated intramolecular Friedel–Crafts alkylation afforded the dihydro-precursors **7a**/**7b**. At this step, the existence of a methoxy group on each corannulene unit ensured a six-membered ring cyclized product only and avoided possible formation of five-membered rings.^10^ Oxidative dehydrogenation of **7a**/**7b** with 2,3-dichloro-5,6-dicyano-1,4-benzoquinone (DDQ) in toluene at 80 °C gave the final desired products **Cor-D1** and **Cor-D2**, respectively. The bulky mesityl groups are essential to kinetically block the reaction sites and also make the final products reasonably soluble. Both **Cor-D1** and **Cor-D2** are stable and can be purified by normal silica gel column chromatography.

### Ground-state geometry

**Fig. 2:**
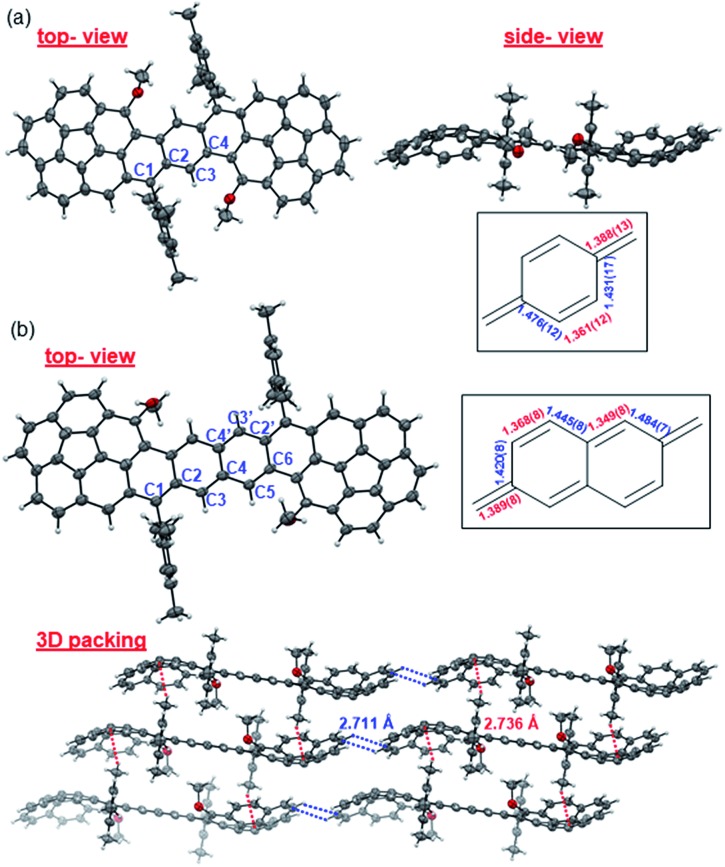
X-ray crystallographic structures of (a) **Cor-D1** and (b) **Cor-D2** (top-view, side-view and 3D packing), together with selected bond lengths (in Å) for the central *p*-QDM and 2,6-NQDM units.

Single crystals of **Cor-D1** and **Cor-D2** suitable for X-ray crystallographic analysis were grown by slow solvent evaporation from chlorobenzene solution.^11^ Both molecules adopt a double-curved geometry, with the two corannulene bowls bent to opposite directions (Fig. 2). The bowl depth (*d*) of **Cor-D1** is measured to be 0.89 Å, which is slightly larger than that of parent corannulene (*d* = 0.87 Å),^12^ and **Cor-D2** shows even larger bowl depth of 0.94 Å. No intermolecular close contact is found between **Cor-D1** molecules, and chlorobenzene molecules fill the void with [CH···π] interactions with backbone of **Cor-D1**. On the other hand, multiple [CH···π] interactions (distance: 2.711 Å) between neighboring corannulene bowls are found in the crystallographic structure of **Cor-D2**, and there are additional [CH···π] interactions between the methyl groups and the corannulene bowls (distance: 2.736 Å), both drive the molecules into a densely packed structure (Fig. 2b). The central *p*-QDM unit in **Cor-D1** has a large bond length alternation (BLA) (Fig. 2a), indicating that the quinoidal structure is more important. However, the *exo*-methylene bond, C1–C2 (1.388(13) Å), is significantly longer than that of typical olefins (1.33–1.34 Å), implying certain contribution of diradical form. The central 2,6-NQDM unit in **Cor-D2** also has significant BLA, with the *exo*-methylene C1–C2 bond being elongated (1.389(8) Å) (Fig. 2b). Natural orbital occupation number (NOON) calculations at UCAM-B3LYP/6-31G(d,p) level based on the X-ray structures suggest that **Cor-D1** and **Cor-D2** have a diradical character (*y*_0_) of 5.4% and 16.9%, respectively. Interestingly, a dihydro-product (**Cor-D2-2H**), the *anti*-isomer of **7b**, was found during the crystal growth of **Cor-D2** by slow evaporation of chlorobenzene at 60 °C. The structure was confirmed by X-ray crystallographic analysis (Fig. S60 in ESI†), and the formation can be explained by the hydrogen abstraction from solvents at the most reactive sites (C1). Variable-temperature (VT) ^1^H NMR spectra of **Cor-D1** in CDCl_3_ (245–335 K) revealed splitting of the protons for both the central benzenoid ring, the OMe group, and the *ortho*-methyl groups in the mesityl substituents below the coalescent temperature *T*_c_ (≈280 K for the central benzene ring protons) (Fig. S1 in ESI†). Due to the bowl-shaped corannulene units, there would be two isomers of **Cor-D1**: one with two terminal bowls bent to the opposite directions (*anti*-form) and the other with two terminal bowls bent to the same directions (*syn*-form). Our calculations (B3LYP/6-31G(d,p)) predict that the *syn*-form is 0.538 kcal mol^–1^ higher in energy than the *anti-*isomer (Table S13 in ESI†), and thus the species with higher NMR integration can be correlated to the *anti*-isomer. The bowl inversion rate constant *k* at different temperatures was estimated by careful line-shape analysis^13^ and fitting of the data by Eyring equation (ln(*k*/*T*) = –Δ*H*^‡^/*R* × 1/*T* + ln(*k*_B_/*h*) + Δ*S*^‡^/*R*) gave thermodynamic parameters Δ*H*^‡^ = 19.8 ± 0.6 kcal mol^–1^ and Δ*S*^‡^ = 21.9 ± 2.3 cal (mol^–1^ K). Accordingly, Δ*G*^‡^ was estimated to be 13.3 ± 1.3 kcal mol^–1^ at 298 K (Fig. S2 and Table S1 in ESI†). This barrier is larger than the parent corannulene (∼11.5 kcal mol^–1^) but slightly smaller than that of the corannulene annulated with a –CH_2_–CE_2_–CH_2_– (E = COOCH_3_) ring (∼15.5 kcal mol^–1^).12*b***Cor-D2** showed similar behavior with slightly lower *T*_c_ (≈270 K for central naphthalene ring protons) (Fig. S3 in ESI†) and line-shape analysis gave Δ*H*^‡^ = 19.7 ± 1.4 kcal mol^–1^, Δ*S*^‡^ = 22.5 ± 5.3 cal (mol^–1^ K^–1^) and Δ*G*^‡^ (298 K) = 13.0 ± 3.0 kcal mol^–1^ (Fig. S4 and Table S2 in ESI†). Calculations show that the *anti*- and *syn*-isomers of **Cor-D2** have nearly identical energy (Table S13 in ESI†), and thus both isomers exist equally in solution.

### Optical, electrochemical and magnetic properties

**Fig. 3:**
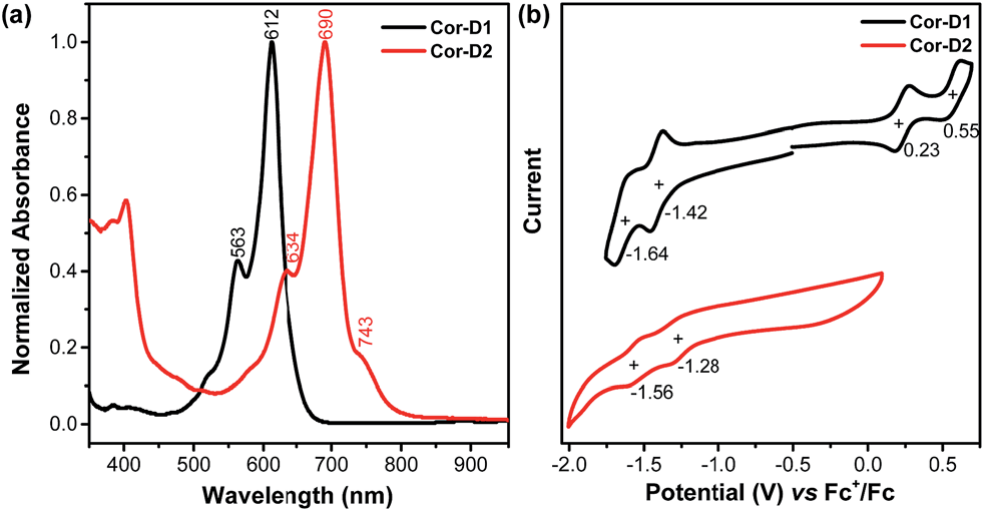
(a) Normalized UV-vis absorption spectra in DCM and (b) cyclic voltammograms of **Cor-D1** and **Cor-D2** in 1,2-DCB at 50 °C.

Compound **Cor-D1** is blue while **Cor-D2** is green in dichloromethane (DCM). **Cor-D1** shows an intense absorption band with maximum (*λ*_max_) at 612 nm (Fig. 3a), which can be correlated to HOMO → LUMO electronic transition based on time-dependent (TD) DFT calculations (see ESI†). **Cor-D2** displays a similar intense absorption band with *λ*_max_ at 690 nm, but with one weak shoulder at 743 nm. The appearance of such weak shoulder at long wavelength is characteristic for many open-shell singlet diradicaloids, which can be mainly correlated to the H,H → L,L double excitation.^14^ This is in accordance with the calculated moderate diradical character for **Cor-D2**. Our TD DFT calculations show that the *anti*- and *syn*-isomers show almost the same absorption spectra (ESI†) and thus one cannot simply distinguish the contribution of the individual isomers.

**Fig. 4:**
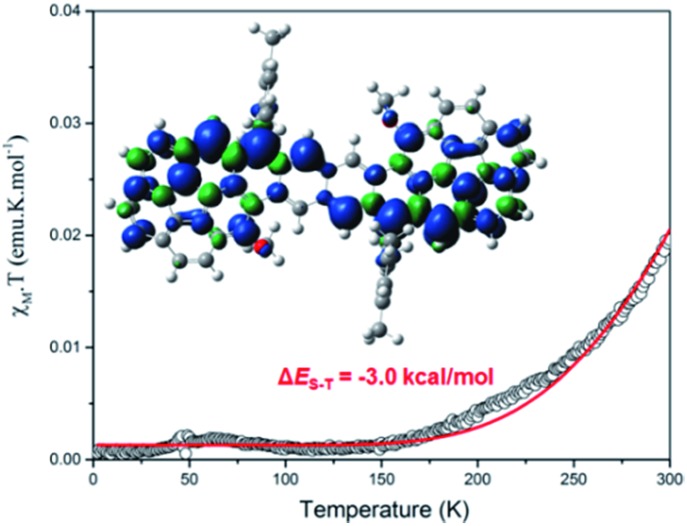
*χ*_M_ *T*–*T* curve in the SQUID measurement for the solid of **Cor-D2** and the red line is the fitted curve by Bleaney–Bows equation. Inset is the calculated spin density distribution map of its triplet biradical.

**Cor-D1** showed sharp NMR spectra in THF-*d*_8_/CS_2_, CDCl_3_ and d-toluene even at 373 K. Its solid powder displayed a weak broad electron spin resonance (ESR) signal (Fig. S5 in ESI†). Heating led to increase of the ESR intensity due to thermal population from the diamagnetic singlet state to the paramagnetic triplet state. Fitting of the *IT*–*T* data by Bleaney–Bowers equation^15^ gave a large singlet-triplet energy gap (Δ*E*_S–T_) of –8.4 kcal mol^–1^ (Fig. S6 in ESI†). Such a large gap is in accordance with its very small diradical character as well as the observed sharp NMR spectra. **Cor-D2** also exhibited sharp NMR spectra in THF-*d*_8_/CS_2_ and CDCl_3_ (from 245 K to 335 K). However, NMR spectral broadening in toluene-*d*_8_ was observed along with the elevated temperatures from 333 K to 373 K (Fig. S10 in ESI†). This phenomenon is consistent with its larger diradical character. The powder of **Cor-D2** exhibited an intense one-line ESR signal with *g*_e_ = 2.0027 at room temperature (Fig. S7 in ESI†), indicating existence of thermally populated paramagnetic species. Superconducting quantum interference device (SQUID) measurement on the powder sample of **Cor-D2** revealed that the product of molar magnetic susceptibility and temperature (*χ*_M_*T*) increased after 150 K and fitting of the data using Bleaney–Bowers equation gave a moderate Δ*E*_S–T_ = –3.0 kcal mol^–1^ (Fig. 4). The calculated spin-density distribution maps for the triplet diradical of **Cor-D2** in both *anti*- and *syn*-forms show that the unpaired electronic spin is delocalized onto the two corannulene bowls, with the largest spin density distributed at the zigzag edges (inset in Fig. 4 and S15 in ESI†).

**Fig. 5:**
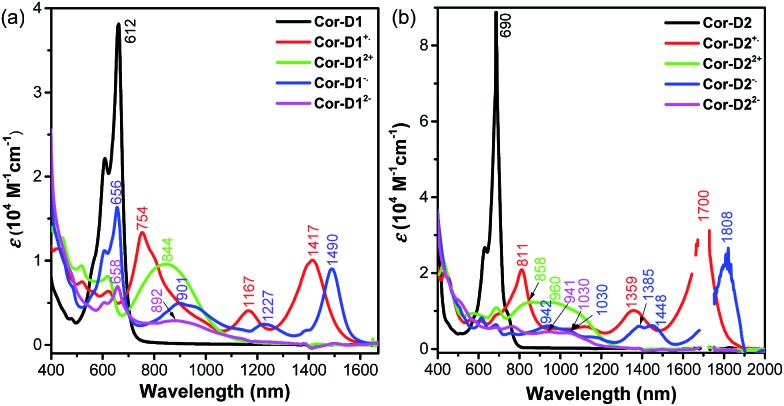
UV-vis-NIR absorption spectra of the neutral, radical cation, dication, radical anion and dianion of **Cor-D1** (a) and **Cor-D2** (b), *ε* is the molar extinction coefficient.

Cyclic voltammetry measurements of **Cor-D1** and **Cor-D2** were carried out in dry 1,2-dichlorobenzene (1,2-DCB) under heating (50 °C) which can suppress molecular aggregation and give better resolved redox waves (Fig. 3b). **Cor-D1** exhibited two oxidation waves with half-wave potential *E*ox1/2 = 0.23 and 0.55 V, and two reduction waves with half-wave potential *E*red1/2 = –1.42 and –1.64 V (*vs.* Fc^+^/Fc). The HOMO and LUMO energy levels were calculated to be –5.03 and –3.38 eV from the onset of the first oxidation/reduction wave, respectively, and an electrochemical energy gap (*E*ECg) of 1.65 eV was estimated. **Cor-D2** showed two reduction waves at *E*red1/2 = –1.28 and –1.56 V, with a LUMO energy level of –3.52 eV. Due to strong aggregation, clear oxidation wave could not be observed. Both compounds can be chemically oxidized into radical cation and dication by NO·SbF_6_ in dry DCM, and reduced into radical anion and dianion by sodium anthracenide (NaAn) in anhydrous THF (Fig. 5 and S11–S12 in ESI†). In both cases, the radical anion and radical cation show similar band structure, indicating a similar electronic structure, but the corresponding bands of radical anion are red-shifted compared to the radical cation (*λ*_max_ = 1490 nm *vs.* 1417 nm in **Cor-D1**, and *λ*_max_ = 1808 nm *vs.* 1700 nm in **Cor-D2**). The dianion and dication exhibit similar band structure and trend (*λ*_max_ = 892 nm *vs.* 844 nm in **Cor-D1**, and *λ*_max_ = 1030 nm *vs.* 960 nm in **Cor-D2**). Clear ^1^H NMR spectra of the dianions of **Cor-D1** and **Cor-D2** can be recorded by *in situ* reduction with NaAn in anhydrous THF-*d*_8_ and most of the aromatic protons appear above 7 ppm despite their electron-rich character (Fig. S13–S14 and S59 in ESI†), indicating that they are strongly aromatic compounds.

**Fig. 6:**
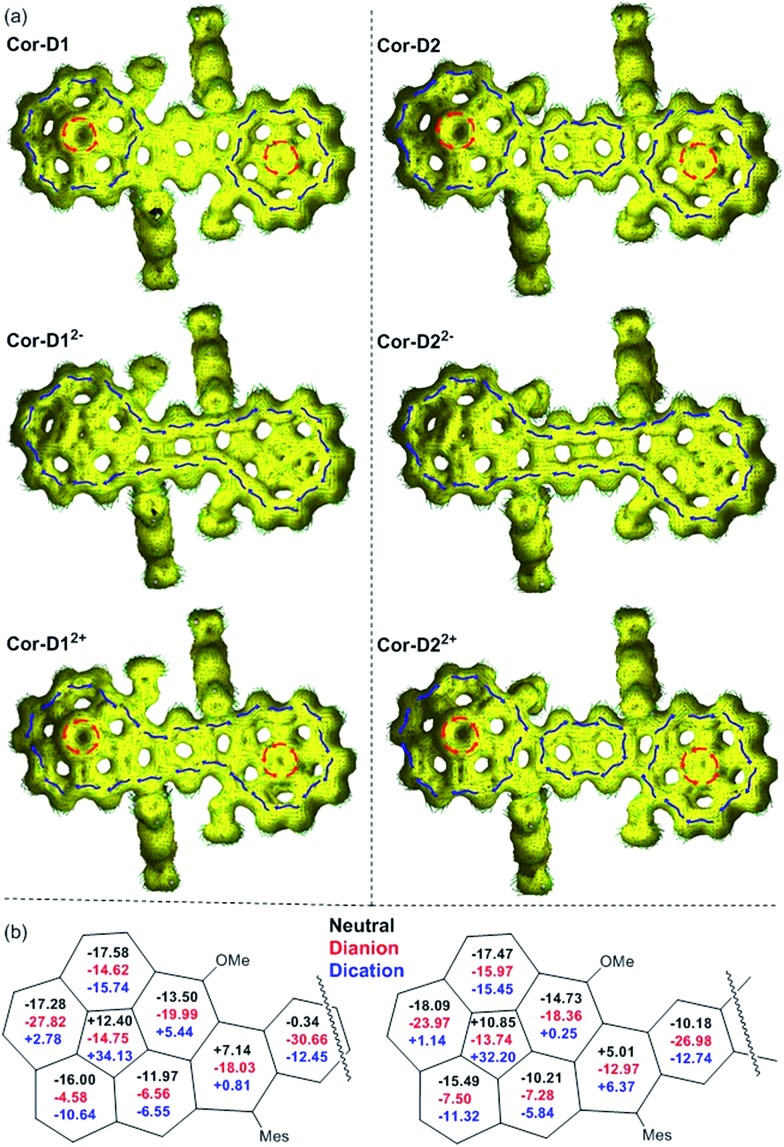
Calculated ACID plots (a) and NICS values (b) of the neutral, dication and dianion of *anti*-**Cor-D1** and **Cor-D2**. For ACID calculations, the magnetic field is perpendicular to the molecular plane and points out through the paper. Isovalue is 0.02, and the blue and red arrows indicate the diamagnetic and paramagnetic ring current flow, respectively. The NICS values are the average of NICS(1)_zz_ and NICS(–1)_zz_ at each individual ring.

To better understand the electronic structure, anisotropy of the induced current-density (ACID) plot^16^ and nucleus independent chemical shift (NICS)^17^ calculations were conducted at (U)B3LYP/6-31G(d,p) level (see Fig. 6 and more images in Fig. S16–S25 in ESI†). The neutral *anti*-**Cor-D1** shows clear diamagnetic/paramagnetic ring current flow along the outer/inner ring of each corannulene bowl, like the parent corannulene (Fig. S26 in ESI†), while there is no ring current circuit at the central *p*-QDM unit, indicating its non-aromatic character. In accordance with this, the average NICS(1)_zz_ value^18^ of the central benzene ring is near zero (–0.34 ppm). However, its dianion shows diamagnetic ring current along the periphery of the whole π-conjugated framework. At the same time, the average NICS(1)_zz_ value of the central benzene ring becomes largely negative (–30.66 ppm), and even the inner cyclopenta-ring in the each corannulene unit shows significantly negative NICS(1)_zz_ value (–14.75 ppm), indicating a strong global aromatic system. Apparently, 38 π electrons can be counted along the periphery, which satisfies Hückel's [4*n* + 2] aromaticity rule. Its dication exhibits similar global aromaticity with 34 π electrons delocalized along the periphery. The NICS(1)_zz_ value of the central benzene ring (–12.45 ppm) is less negative than that in dianion, and that of the cyclopenta-ring becomes quite positive (+34.13 ppm), indicating relatively weak global aromaticity. In the *anti*-**Cor-D2**, the two corannulene bowls remain their individual aromatic character similar to that in **Cor-D1**, but diamagnetic ring current was found in the central naphthalene unit (NICS(1)_zz_: –10.18 ppm), indicating significant contribution of the open-shell diradical form. Its dianion also show global aromaticity with 42 π-electrons delocalized along the periphery and the NICS(1)_zz_ value in the central naphthalene ring is shifted to –26.98 ppm. Its dication does not display global aromaticity as **Cor-D1^2+^**, and the localized aromaticity at the central naphthalene unit dominates (NICS(1)_zz_: –12.74 ppm), making the two corannulene units somehow segregated. The radical anions and radical cations of both compounds with unpaired electron show partial global aromatic character (Fig. S22–S25 in ESI†).

## Conclusions

In summary, rigid, *p*-QDM and 2,6-NQDM bridged corannulene dimers **Cor-D1** and **Cor-D2** were synthesized in stable form, which represent a new type of curved π-conjugated open-shell singlet diradicaloids. **Cor-D1** has a small diradical character and behaves more like a closed-shell hydrocarbon at room temperature, while **Cor-D2** possesses a moderate diradical character. Both compounds display magnetic activity at elevated temperatures. Electronic spins are distributed onto both corannulene bowls in **Cor-D2**, and stronger intermolecular interactions and more ordered packing are observed in its single crystals. Notably, their dianions show prominent global aromaticity with [4*n* + 2] π electrons delocalized along the periphery. Our studies reveal the unique electronic structure of such kind of bowl-shaped π-conjugated polycyclic hydrocarbons, which implies their potential applications for organic electronic and spintronic devices in the future.

## Conflicts of interest

There are no conflicts to declare.

## Supplementary Material

Supplementary information

Crystal structure data

## References

[cit1] (c) Nakano M., Excitation Energies and Properties of Open-Shell Singlet Molecules, Springer, New York, 2014.

[cit2] Ma J., Liu J., Baumgarten M., Fu Y., Tan Y.-Z., Schellhammer K. S., Ortmann F., Cuniberti G., Komber H., Berger R., Müllen K., Feng X. (2017). Angew. Chem., Int. Ed..

[cit3] Janata J., Gendell J., Ling C.-Y., Barth W., Backs L., Mark Jr H. B., Lawton R. G. (1967). J. Am. Chem. Soc..

[cit4] Morita Y., Nishida S., Kobayashi T., Fukui K., Sato K., Shiomi D., Takui T., Nakasuji K. (2004). Org. Lett..

[cit5] Nishida S., Morita Y., Ueda A., Kobayashi T., Fukui K., Ogasawara K., Sato K., Takui T., Nakasuji K. (2008). J. Am. Chem. Soc..

[cit6] Ueda A., Nishida S., Fukui K., Ise T., Shiomi D., Sato K., Takui T., Nakasuji K., Morita Y. (2010). Angew. Chem., Int. Ed..

[cit7] Wu Y.-T., Siegel J. S. (2006). Chem. Rev..

[cit8] Sygula A., Sygula A., Kobryn L. (2008). Org. Lett..

[cit9] Hu P., Lee S., Park K. H., Das S., Herng T. S., Gonçalves T. P., Huang K.-W., Ding J., Kim D., Wu J. (2016). J. Org. Chem..

[cit10] Ota K., Tanaka T., Osuka A. (2014). Org. Lett..

[cit11] CCDC no. for Cor-D1, Cor-D2 and Cor-D2-2H are 1570628, ; 1819179, ; 1819178, respectively.

[cit12] Biedermann P. U., Pogodin S., Agranat I. (1999). J. Org. Chem..

[cit13] Shanan-Atidi H., Bar-Eli K. H. (1970). J. Phys. Chem..

[cit14] Motta S. D., Negri F., Fazzi D., Castiglioni C., Canesi E. V. (2010). J. Phys. Chem. Lett..

[cit15] Bleaney B., Bowers K. D. (1952). Proc. R. Soc. London, Ser. A.

[cit16] Geuenich D., Hess K., Köhler F., Herges R. (2005). Chem. Rev..

[cit17] Chen Z., Wannere C. S., Corminboeuf C., Puchta R., Schleyer P. V. R. (2005). Chem. Rev..

[cit18] Reisi-Vanani A., Rezaei A. A. (2015). J. Mol. Graphics Modell..

