# Supplementary material for: Curved π-conjugated corannulene dimer diradicaloids
Source: Chem Sci. 2018 May 16;9(22):5100–5. doi: 10.1039/c8sc01388h (PMC5994875; doi:10.1039/c8sc01388h)
Supplement: Supplementary file 1 [file SC-009-C8SC01388H-s001.pdf]

Electronic Supplementary Material (ESI) for Chemical Science.
This journal is © The Royal Society of Chemistry 2018

Electronic Supplementary Information (ESI)

Table of Contents

1. Experimental section……………………………………………………………………S2

1.1 General…………………………………………………………………………...S2

1.2 Synthetic procedures and characterization data……………………………………….S3

2. Data analysis for the bowl inversion barrier………………………………………………S7

3. ESR data……..…………….……………………………….….……................................. S12

4. Additional spectra……..………….…………………………………….….……....................S14

5. DFT calculations……..……………..…………………………………………………....S18

6. X-ray crystallographic data……...…………………………………………….….…...S53

7. Appendix: NMR spectra and HR mass spectra……………………………………..……S57

8. References……………………………………………………………………………..S60

1. Experiment Section

1.1 General

All reagents and starting materials were obtained from commercial suppliers and used without further purification. Anhydrous *N*,*N*-dimethylformamide (DMF) and dichloromathane (DCM) were distilled from CaH_2. Anhydrous toluene and THF were distilled from sodium-benzophenone immediately prior to use. The compound **1** was synthesized according to the literature.[1] The ^{1}H NMR and ^{13}C NMR spectra were recorded in deuterated solvents on Bruker DPX 300/400/500 NMR spectrometers with tetramethylsilane (TMS) as the internal standard. The following abbreviations were used to explain the multiplicities: s = singlet, d = doublet, t = triplet, m = multiplet. HR-APCI mass spectra (MS) were recorded on a Bruker amazonX instrument. EI mass spectra were recorded on Agilent 5975C DIP/MS mass spectrometer. The solvents used for UV-vis and PL measurements are of HPLC grade (Merck). The electrochemical measurements were carried out in anhydrous 1,2-dichlorobenzene with 0.1 M tetra-*n*-butylammonium perchlorate (*n*-Bu_4NClO_4) as the supporting electrolyte at 50 ^{o}C under the protection of nitrogen. A gold disk was used as working electrode, platinum wire was used as counting electrode, and Ag/AgCl (3M KCl solution) as reference electrode. The potential was externally calibrated against the ferrocene/ferrocenium couple. Steady-state UV-vis absorption were recorded on a Shimadzu UV-1700 and UV-3600 spectrometer. Continuous wave X-band ESR spectra were obtained with a JEOL (FA200) spectrometer.

A Quantum Design 7 Tesla SQUID-VSM system was available for the magnetic measurements in this work. Powder sample of **Cor-D2** with a weight of 5-10 mg was sealed in a plastic capsule. Magnetic moment was measured in the temperature range of 2 to 300 K. The empty plastic capsule exhibited diamagnetic and its magnetic moment was measured for correction. After correction of diamagnetic contributions from the sample, using tabulated constants, sample holder and paramagnetic contamination, the magnetic data were fitted with Bleaney-Bowers equation:

$$\chi_M T = \frac{2N\beta^2 g^2}{k_B[3 + exp(-2J/k_B T)]}$$

where, -2J is correlated to the excitation energy from the singlet ground state to the triplet excited state.

1.2. Synthetic procedure and characterization data

1-Bromo-2-methoxycorannulene (**2**)

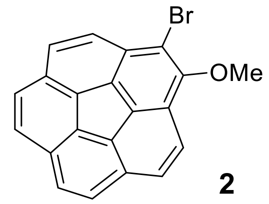

2

N-bromosuccinimide (NBS, 358.0 mg) was added to a solution of compound **1** (500.0 mg, 1.78 mmol) and i-Pr_2NH (18 mg) in DCM. The mixture was stirred for 15 min until the starting material **1** was consumed completely, and then quenched with 100 mL H_2O. The mixture was extracted with DCM for three times, and the organic phase was washed with brine for three times and dried over Mg_2SO_4. The solvent was removed under reduce pressure and the residue was purified with silica gel chromatography to give pure compound **2** in 85% yield. ^{1}H NMR ($CDCl_3$, 300 MHz): δ ppm 7.90 (1H, d, J = 8.7 Hz), 7.88 (1H, d, J = 8.8 Hz), 7.80-7.73 (6H, m), 4.34 (3H, s). ^{13}C NMR ($CDCl_3$, 125 MHz): δ ppm 158.48, 136.39, 136.29, 136.17, 134.90, 133.10, 131.26, 130.91, 130.84, 129.38, 127.36, 127.33, 126.99, 126.78, 126.66, 126.58, 125.76, 124.32, 123.66, 102.58, 55.97. HR-MS (APCI): m/z = 359.0066, calcd. For $C_{21}H_{12}BrO$ $[M+1]^+$: m/z = 359.0072, error = -1.67 ppm.

1-Methoxy-corannulene-2-pinacol boronate (**3**)

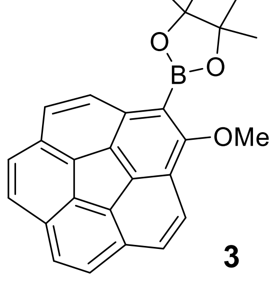

3

A solution of *n*-BuLi in *n*-hexane (2.10 M, 0.32 mL, 1.20 equiv.) was added dropwise into a solution of compound **2** (2.0 g, 5.60 mmol) in 50.0 mL dry THF under nitrogen. The mixture was stirred for 3hrs at -78 ºC, then 2-isopropoxy-4,4,5,5-tetramethyl-1,3,2-dioxaborolane (1.50 g, 8.30 mmol) was added. The reaction mixture was warmed to rt and stirred overnight. The reaction was quenched by water at 0 ºC, and was extracted with DCM. The organic layer was washed with brine for three times and dried over Mg_2SO_4. The solvent was removed and the residue was purified with column chromatography to give compound **3** as a white solid (1.6 g, 70% yield). ^{1}H NMR ($CDCl_3$, 300 MHz): δ ppm 8.12 (1H, d, *J* = 6.6 Hz), 7.94 (1H, d, *J* = 6.3 Hz), 7.83-7.76 (6 H, m), 4.34 (3H, s), 1.48 (12 H, s). ^{13}C NMR ($CDCl_3$, 125 MHz): δ ppm 164.80, 138.46, 136.42, 135.88, 135.66, 135.32, 132.63, 131.39, 130.83, 130.05, 128.30, 127.75, 127.16, 127.12, 126.80, 126.74, 126.25, 125.55, 124.00, 83.76, 64.34, 25.01. HR-MS (APCI): *m/z* = 407.1818, calcd. For $C_{27}H_{24}BO_3$ $[M+1]^+$: *m/z* = 407.1819, error = -0.25 ppm.

Compound **Cor-D1**

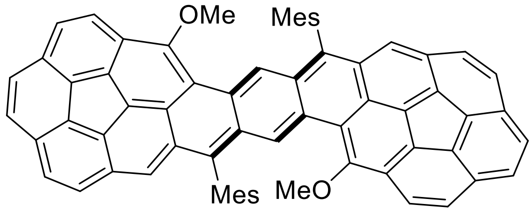

Cor-D1

A mixture of compound **3** (518 mg, 1.28 mmol), 2,5-dibromoterephthalaldehyde (**4**, 150.0 mg, 0.51 mmol), K_2CO_3 (1.80 g, 13.0 mmol), $Pd(PPh_3)_4$ (59.0 mg, 0.051 mmol) in toluene/EtOH/H_2O (2/1/1) (16 mL) was stirred at 110 ºC for 24 hrs under nitrogen. After cooling down, the mixture was extracted with DCM for three times and the organic phase was washed with brine for three times, dried with anhydrous $MgSO_4$. The solvent was removed under vacuum and the residue was washed with DCM and hexane, dried under vacuum. The solubility of the residue was poor so that intermediate dialdehyde compound **6a** was used directly to the next step without purification. Mesitylmagnesium bromide (1.0 M, 1.0 mL) was added to the 15.0 mL dry THF solution of compound **6a** (0.07 g, 0.10 mmol) under argon atmosphere and the solution was stirred at room temperature overnight. The reaction mixture was quenched by water and extracted by dichloromethane. The organic layer

was dried over Na_2SO_4 and the solvent was removed under reduced pressure. The crude compound was then dissolved in 20.0 mL dry DCM under argon atmosphere and 0.2 mL of $BF_3{\cdot}OEt_2$ was added. The mixture was stirred for 5 minutes and quenched by methanol. The solvent was removed under reduced pressure. DDQ (0.0227 g, 0.10 mmol) was added dropwise to the 20.0 mL dry toluene solution of the cyclized intermediate compound **7a** and the reaction mixture was heated to 80 ºC for half hour. The solvent was remvoed and the the residue was purified by column chromatography (silica gel, DCM/hexane (1/1, v/v) as eluent) to give compound **Cor-D1** (13.0 mg, 15% yield in three steps) as a dark black solid. HR-MS (APCI): *m*/*z* = 893.3419, calcd. For $C_{68}H_{45}O_2$ $[M+1]^+$: *m*/*z* = 893.3414, error = -0.5 ppm. ^1H NMR (THF-d_8/CS_2 = 2:1, v/v, 400 MHz, rt): δ ppm 8.49 (2H, s), 7.90 (2H, d, *J* = 8.8 Hz), 7.78 (2H, d, *J* = 8.9 Hz), 7.72 (4H, s), 7.65 (2H, d, *J* = 8.8 Hz), 7.56 (2 H, d, *J* = 8.8 Hz), 7.21 (4H, br), 7.03 (2H, s), 3.86 (6H, s), 2.54 (6H, s). Clear ^{13}C NMR spectrum could not be obtained due to the poor solubility of the compound. HR-MS (APCI): *m*/*z* = 893.3419, calcd. For $C_{68}H_{45}O_2$ $[M+1]^+$: *m*/*z* = 893.3414, error = -0.5 ppm. The dianion of **Cor-D1** was prepared by reduction with 2.1 equivalent of freshly prepared sodium anthracenide in anhydrous THF-d_8 in a J-Y NMR tube. ^1H NMR (THF-d_8, 300 MHz, rt): δ ppm 9.37 (2H, s), 7.80 (2H, d, *J* = 8.6 Hz), 7.56 (2H, d, *J* = 9.0 Hz), 7.27 (2H, d, *J* = 7.8 Hz), 7.15-7.05 (10H, m), 6.95 (2 H, d, *J* = 9.1 Hz), 2.48 (6H, s).

Compound **Cor-D2**

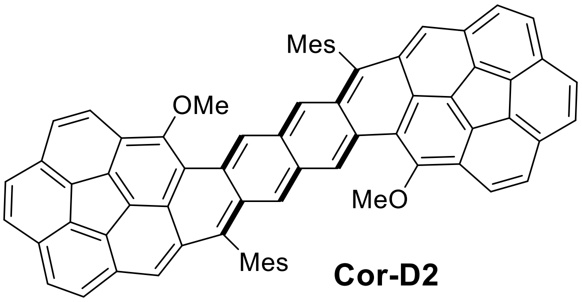

Cor-D2

A mixture of compound **3** (518 mg, 1.28 mmol), 3,7-diformylnaphthalene-2,6-diyl bis(trifluoromethanesulfonate)[2] (**5**, 240 mg, 0.50 mmol), K_2CO_3 (1.80 g, 13.0 mmol), $Pd(PPh_3)_4$ (59.0 mg, 0.051 mmol) in toluene/EtOH/H_2O (2/1/1) (16 mL) was stirred at 110 ºC

for 24 hrs under nitrogen. After cooling, the mixture was extracted with DCM for three times, and the organic phase was washed with brine for three times, dried over anhydrous $MgSO_4$. The solvent was removed under vacuum to give a solid residue, which was washed with DCM and hexane and dried under vacuum. The solubility of intermediate dialdehye compound **6b** was very poor so it was used directly to the next step without purification. Mesitylmagnesium bromide (1.0 M, 1.0 mL) was added to the 15.0 mL dry THF solution of compound **6b** (74.0 mg, 0.10 mmol) under argon atmosphere, and the solution was stirred at room temperature overnight. The reaction mixture was quenched by water and extracted by DCM. The organic layer was dried over Na_2SO_4 and the solvent was removed under reduced pressure. The crude compound was then dissolved in 20.0 mL dry DCM under argon atmosphere and 0.2 mL of $BF_3{\cdot}OEt_2$ was added. The mixture was stirred for 5 minutes and quenched by methanol. The solvent was removed under reduced pressure. DDQ (0.0227 g, 0.10 mmol) was added to the 20.0 mL dry toluene solution of intermediate cyclized product **7b**, and the reaction mixture was heated to 80 ºC for half hour. The solvent was removed under vacuum and the residue was purified by column chromatography (silica gel, DCM/hexane (1/1, v/v) as eluent) to give compound **Cor-D2** (18.0 mg, 19% yield in three steps) as a dark black solid. ^{1}H NMR (THF-d_8, 400 MHz, rt): δ ppm 8.70 (2H, s), 8.04 (2H, d, J = 8.9 Hz), 7.83 (2H, d, J = 8.9 Hz), 7.73 (4H, s), 7.65 (2H, d, J = 8.8 Hz), 7.53 (2H, d, J = 8.8 Hz), 7.15 (4H, br), 7.09 (2 H, s), 6.94 (2H, s), 4.19 (6H, s), 2.52 (6H, s). Clear ^{13}C NMR spectrum could not be obtained due to the poor solubility of the compound. HR-MS (APCI): m/z = 943.3572, calcd. For $C_{72}H_{46}O_2$ $[M+1]^+$: m/z = 943.3571, error = -0.2 ppm. The dianion of **Cor-D2** was prepared by reduction with 2.1 equivalent of freshly prepared sodium anthracenide in anhydrous THF-d_8 in a J-Y NMR tube. ^{1}H NMR (THF-d_8, 400 MHz, rt): δ ppm 9.77 (2H, s), 8.09 (2H, d, J = 8.1 Hz), 7.77 (2H, d, J = 9.0 Hz), 7.72-7.72 (4H, m), 7.61 (2H, d, J = 7.9 Hz), 7.17 (4 H, s), 4.04 (6H, s), 2.48 (6H, s).

2. Data analysis for the bowl inversion barrier

VT ^{1}H NMR spectra of **Cor-D1** and **Cor-D2** were recorded in $CDCl_3$ from 245 K to 335 K. The bowl inversion exchange rate constant k was estimated based on the line-shape change of the protons ***a*** in the central benzene/naphthalene rings.[3] The exchange rate constant k was calculated by individual equation: (1) At slow exchange temperature (T < T_c), two peaks are separated enough, then $k = \pi[(\Delta V_e)_{1/2}-(\Delta V_0)_{1/2}]$. (2) At coalescence temperature (T = T_c), $k = \pi\Delta V_0/2^{1/2}$. In these equations, ΔV is the difference in chemical shift (Hz) between two correlated peaks at one temperature in the slow exchange region. ΔV_0 is defined as the value of ΔV at no exchange temperature (at which the two peaks are mostly separated, herein, it was chosen as 245 K), and ΔV_e is defined as the value of ΔV at all other temperatures in this region if available. $(\Delta V)_{1/2}$ is the linewidth (Hz) at half height of peak at anyone temperature in all region if available. $(\Delta V_0)_{1/2}$ is defined as the value of $(\Delta V)_{1/2}$ at no exchange temperature. $(\Delta V_e)_{1/2}$ is defined as the value of $(\Delta V)_{1/2}$ at all other temperatures in all region if available. T_c is defined as coalescence temperature at which two peaks completely merge into one peak. The obtained k values were then fitted with Eyring equation: $\ln\frac{k}{T} = -\frac{\Delta H^\ddagger}{R} \times \frac{1}{T} + \ln\frac{k_B}{h} + \frac{\Delta S^\ddagger}{R}$ to obtain the thermodynamic parameters $\Delta H^\ddagger$ and $\Delta S^\ddagger$, and then $\Delta G^\ddagger = \Delta H^\ddagger - T\Delta S^\ddagger$.

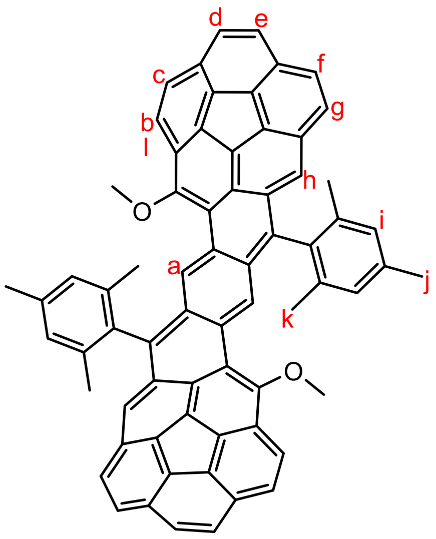

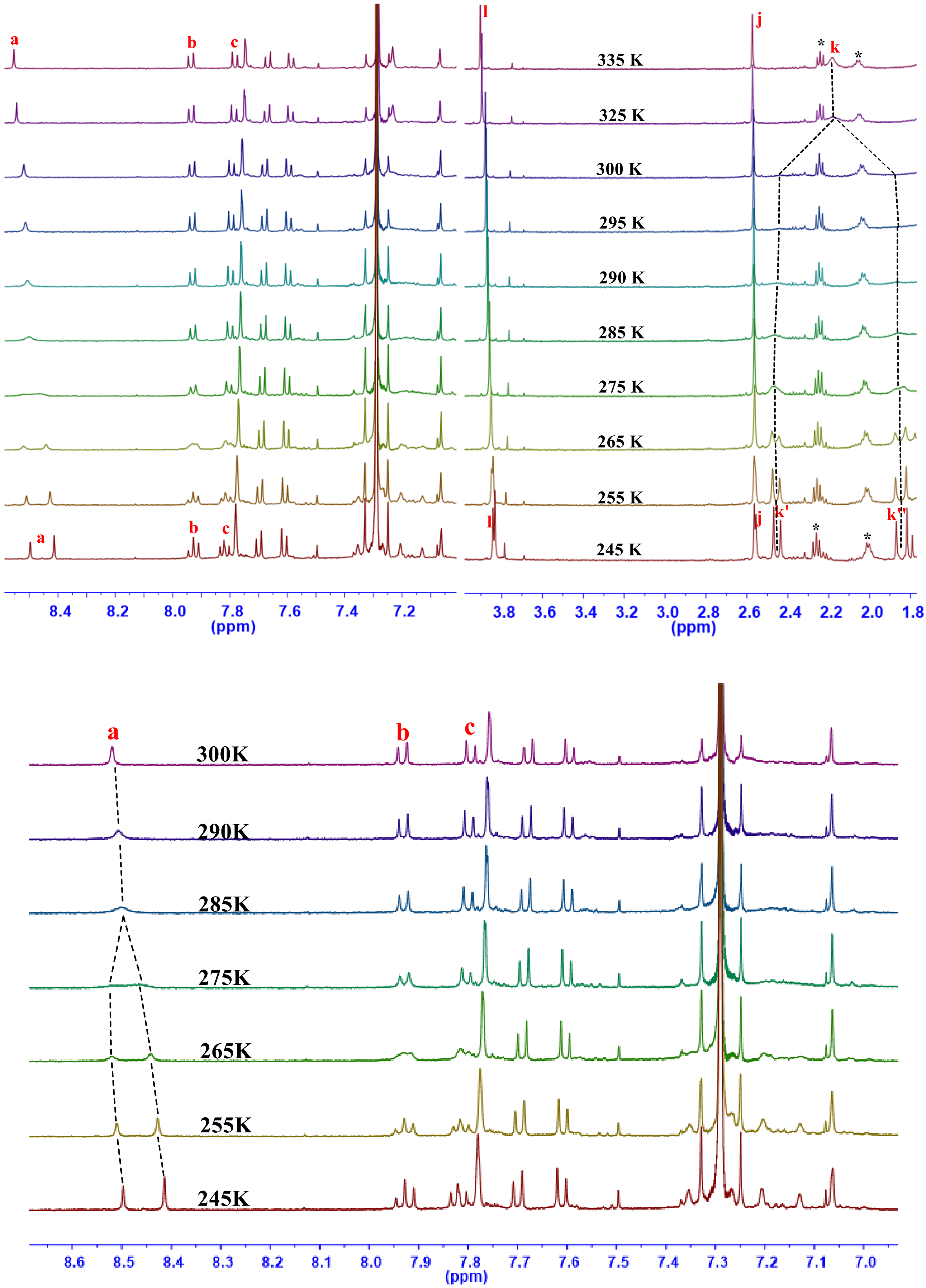

Fig. S1. VT NMR spectra of **Cor-D1** in $CDCl_3$ (245-335 K, 500 MHz). Top: full spectra; bottom: magnified spectra at aromatic region. * indicates impurities that cannot be removed even by column chromatography and repeated washing.

Table S1. Parameters obtained from the line-shape analysis of the resonances for proton ***a*** based on the VT ^{1}H NMR spectra of **Cor-D1** in $CDCl_3$.

T (K)	$(\Delta V_e)_{1/2}$ (Hz)	ΔV_e (Hz)	k (s^{-1})
300	3.44	—	1751.41
290	8.53	—	409.26
285	15.20	—	204.20
275	—	24.17	75.21
265	6.76	40.03	15.30
255	3.09	41.35	3.78
245	1.89 ($(\Delta V_0)_{1/2}$)	41.6 (ΔV_0)	

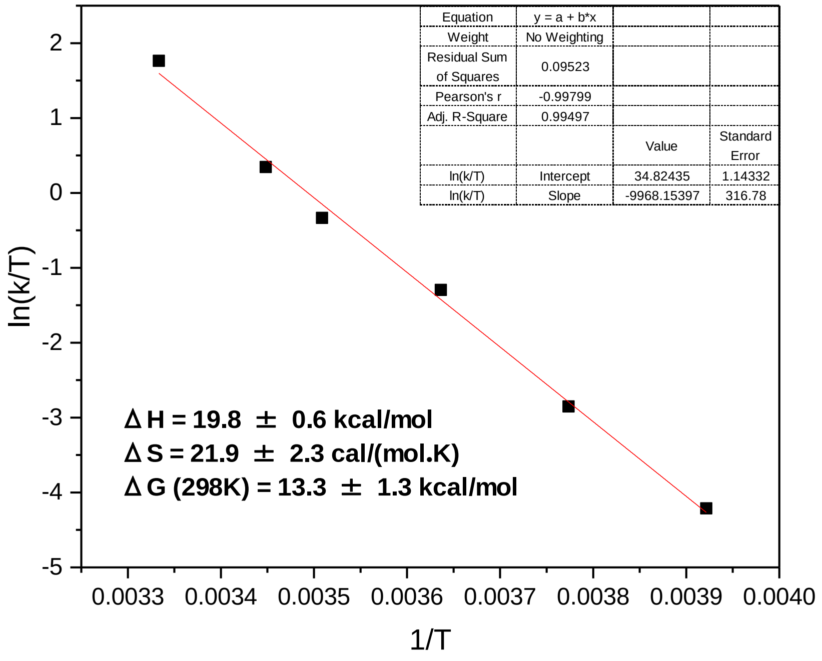

Fig. S2. Fitting of the exchange rate constants with temperature by Erying equation for **Cor-D1** in $CDCl_3$.

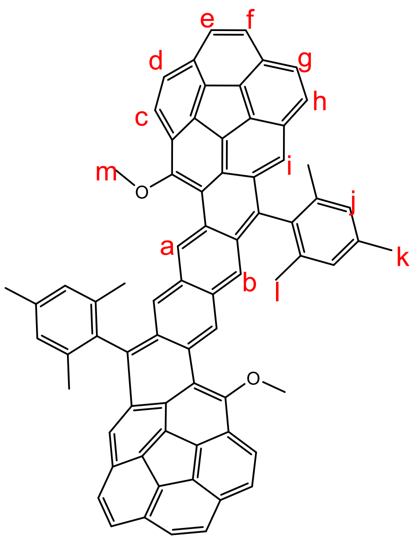

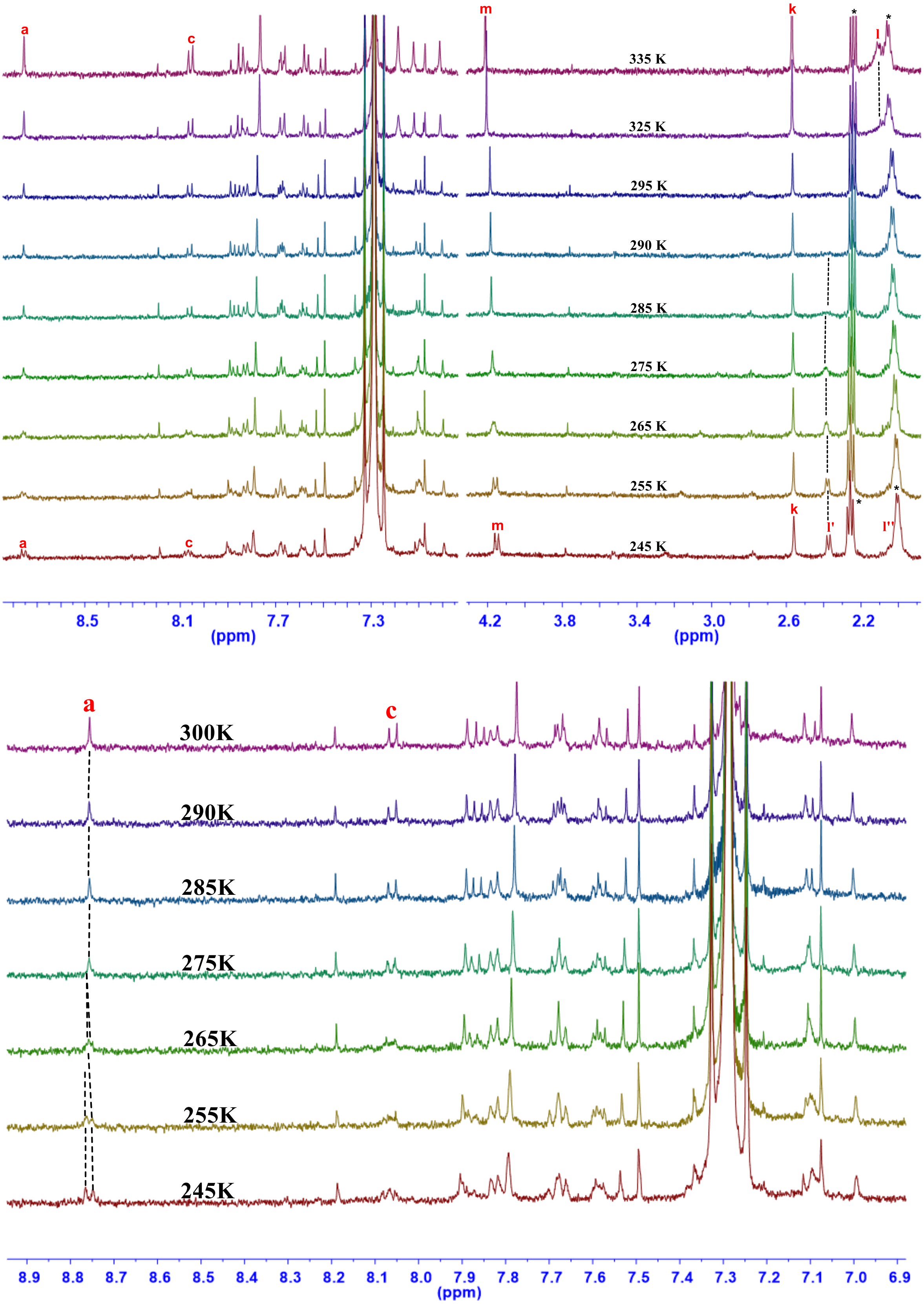

Fig. S3. VT NMR spectra of **Cor-D2** in $CDCl_3$ (245-335 K, 500 MHz). Top: full spectra; bottom: magnified spectra at aromatic region. * indicates impurities that cannot be removed even by column chromatography and repeated washing.

Table S2. Parameters obtained from the line-shape analysis of the resonances for proton ***a*** based on the VT ^{1}H NMR spectra of **Cor-D2** in $CDCl_3$.

T (K)	$(\Delta V_e)_{1/2}$ (Hz)	ΔV_e (Hz)	k (s^{-1})
290	2.48	—	678.63
285	2.77	—	250.80
275	3.30	—	116.31
265	—	4.25	16.53
255	3.96	7.01	5.17
245	2.31($(\Delta V_0)_{1/2}$)	8.57 (ΔV_0)	

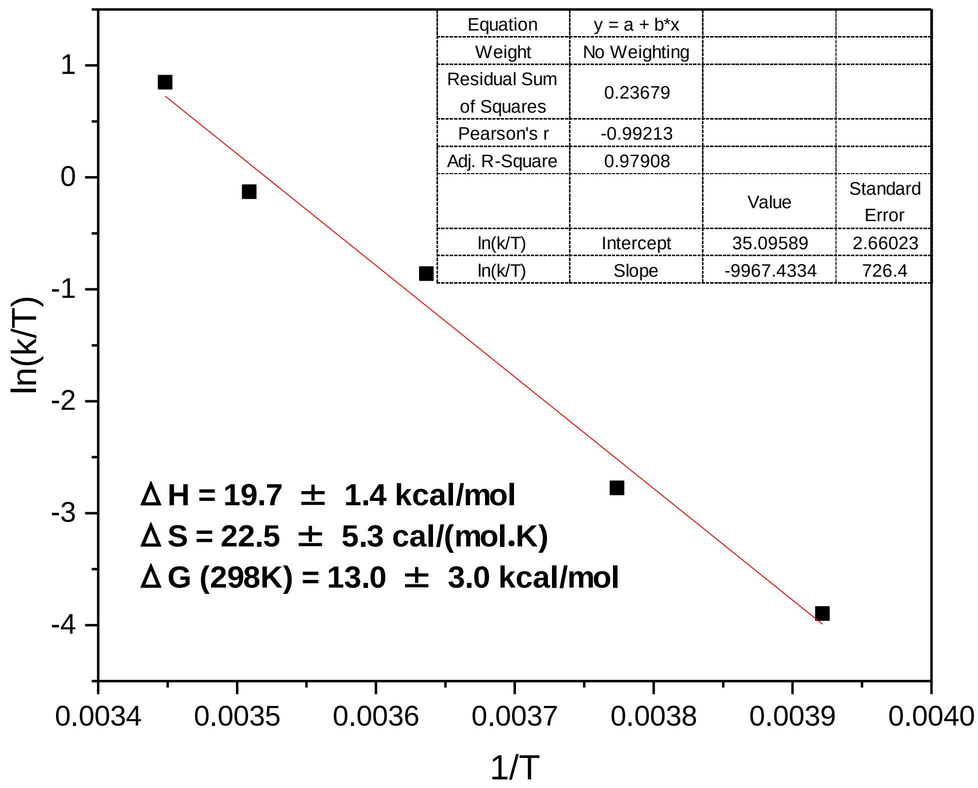

Fig. S4. Fitting of the exchange rate constants with temperature by Erying equation for **Cor-D2** in $CDCl_3$.

3. ESR data

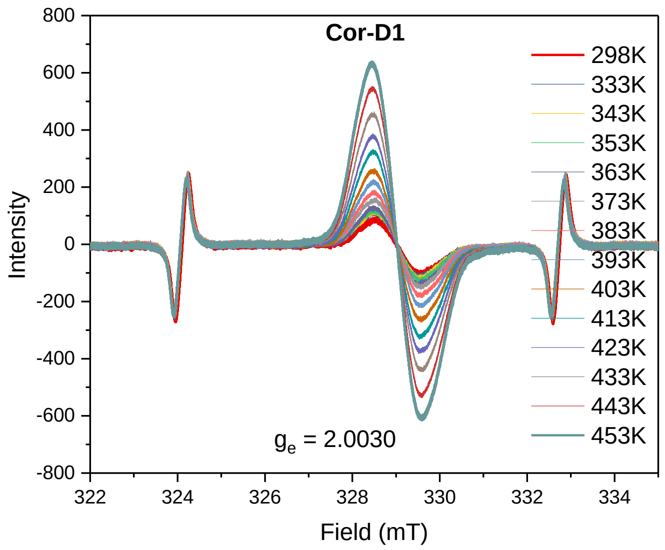

Fig. S5. Solid-state ESR spectra of **Cor-D1** recorded from 298 K to 453K.

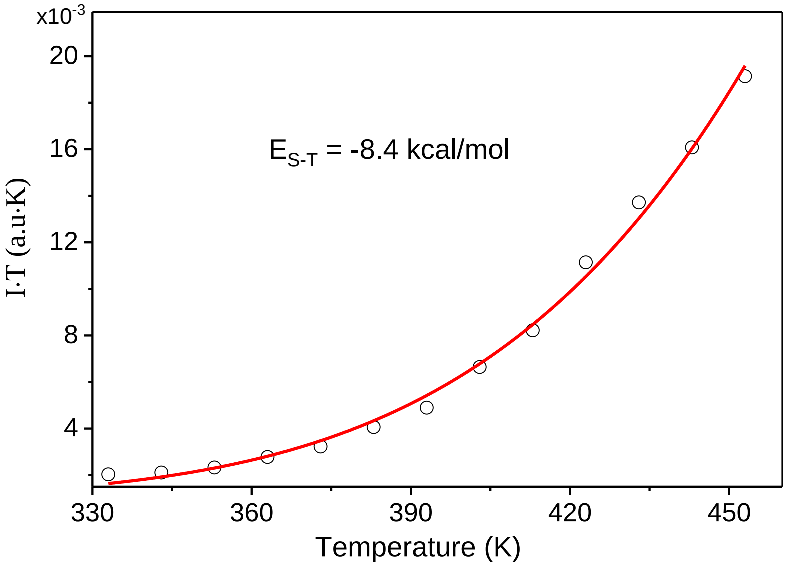

Fig. S6. *IT–T* curve based on the VT ESR spectra of the powder **Cor-D1** upon heating up to 453 K; the red line denotes the fitted curve obtained by using the Bleaney–Bows equation to give a singlet-triplet energy gap of -8.4 kcal/mol. *I* is the integrated ESR intensity and *T* is temperature.

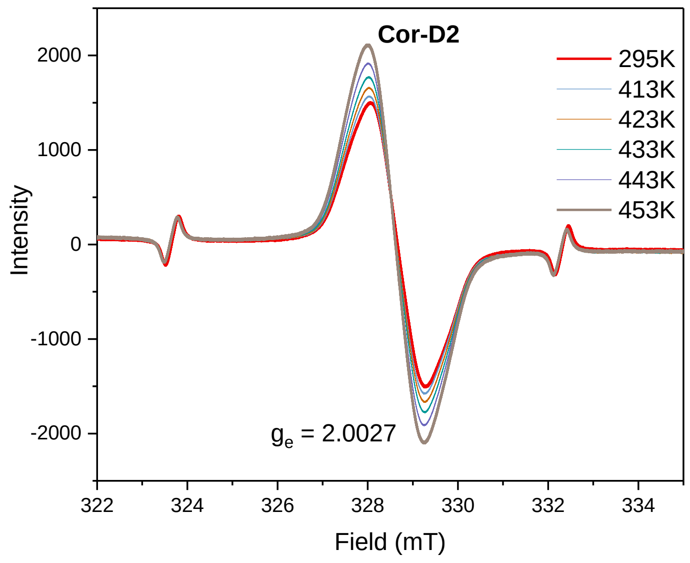

Fig. S7. VT ESR spectra of compound **Cor-D2** at solid state.

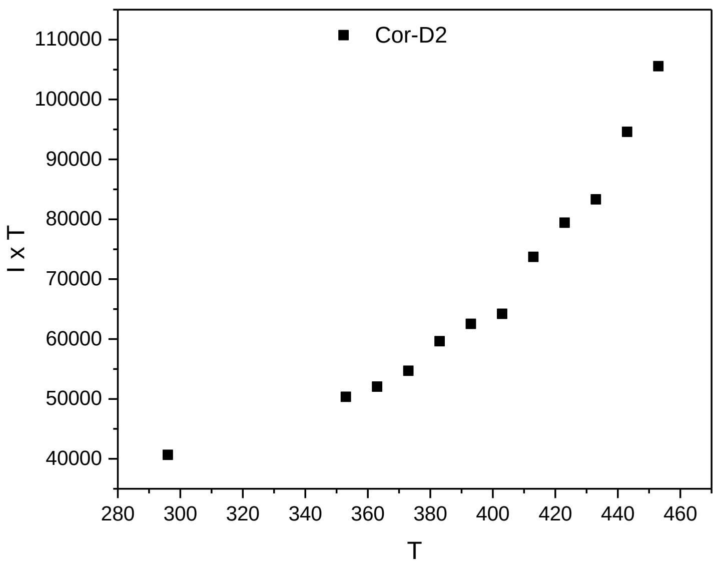

Fig. S8. The change of *IT* with temperature *T* for **Cor-D2**. *I* is the integrated ESR intensity and *T* is temperature.

4. Additional spectra

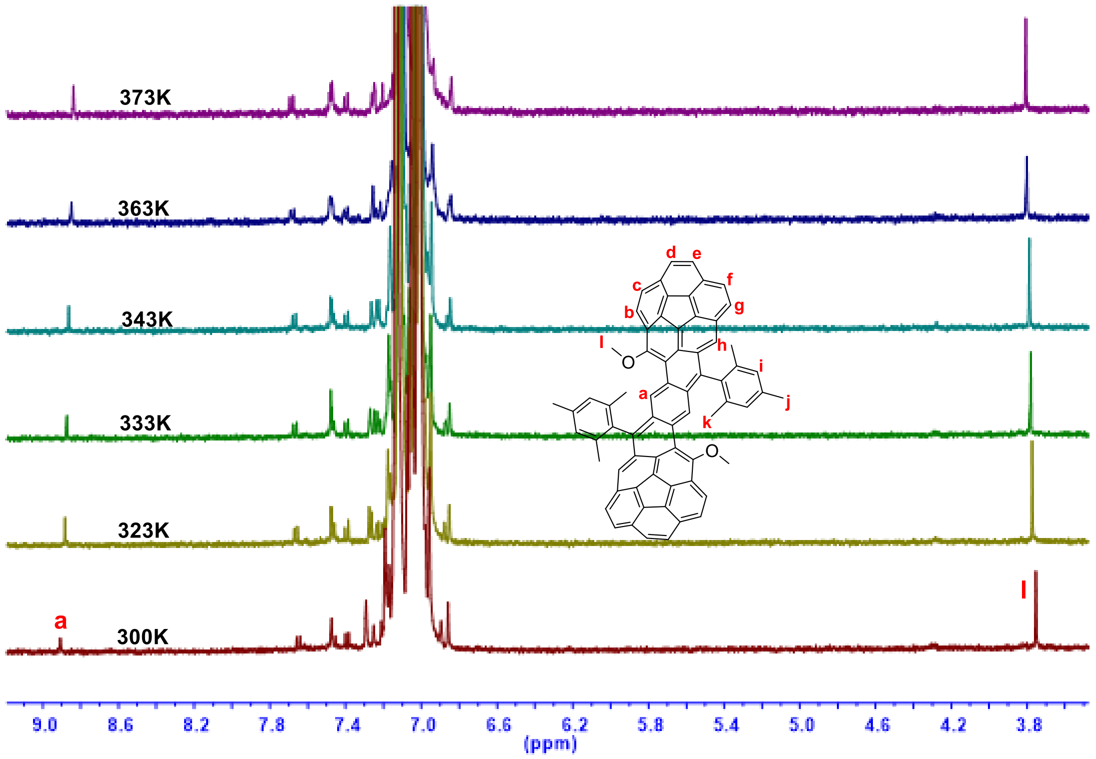

Fig. S9. VT NMR spectra of **Cor-D1** in *d*-toluene (300-373 K, 500 MHz).

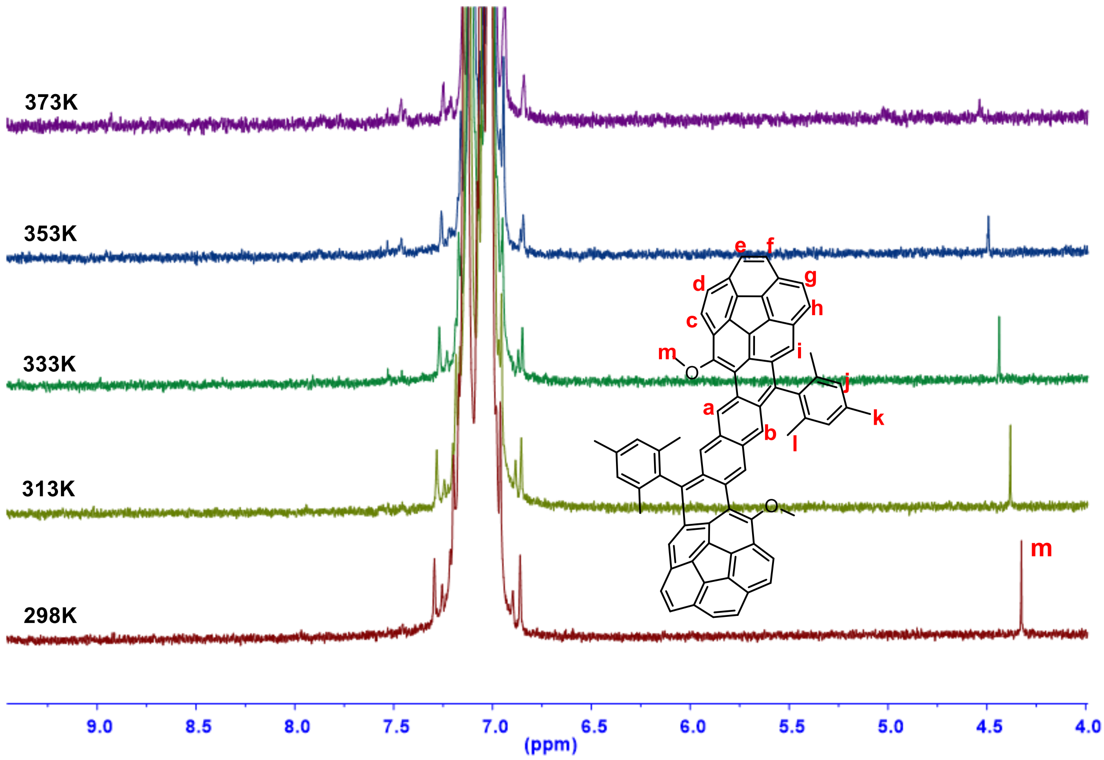

Fig. S10. VT NMR spectra of **Cor-D2** in *d*-toluene (298-373 K, 500 MHz). No signal in the aromatic range is observed in this solvent at all temperatures, due to its extreme poor solubility in *d*-toluene. But protons m on methoxy groups are visible.

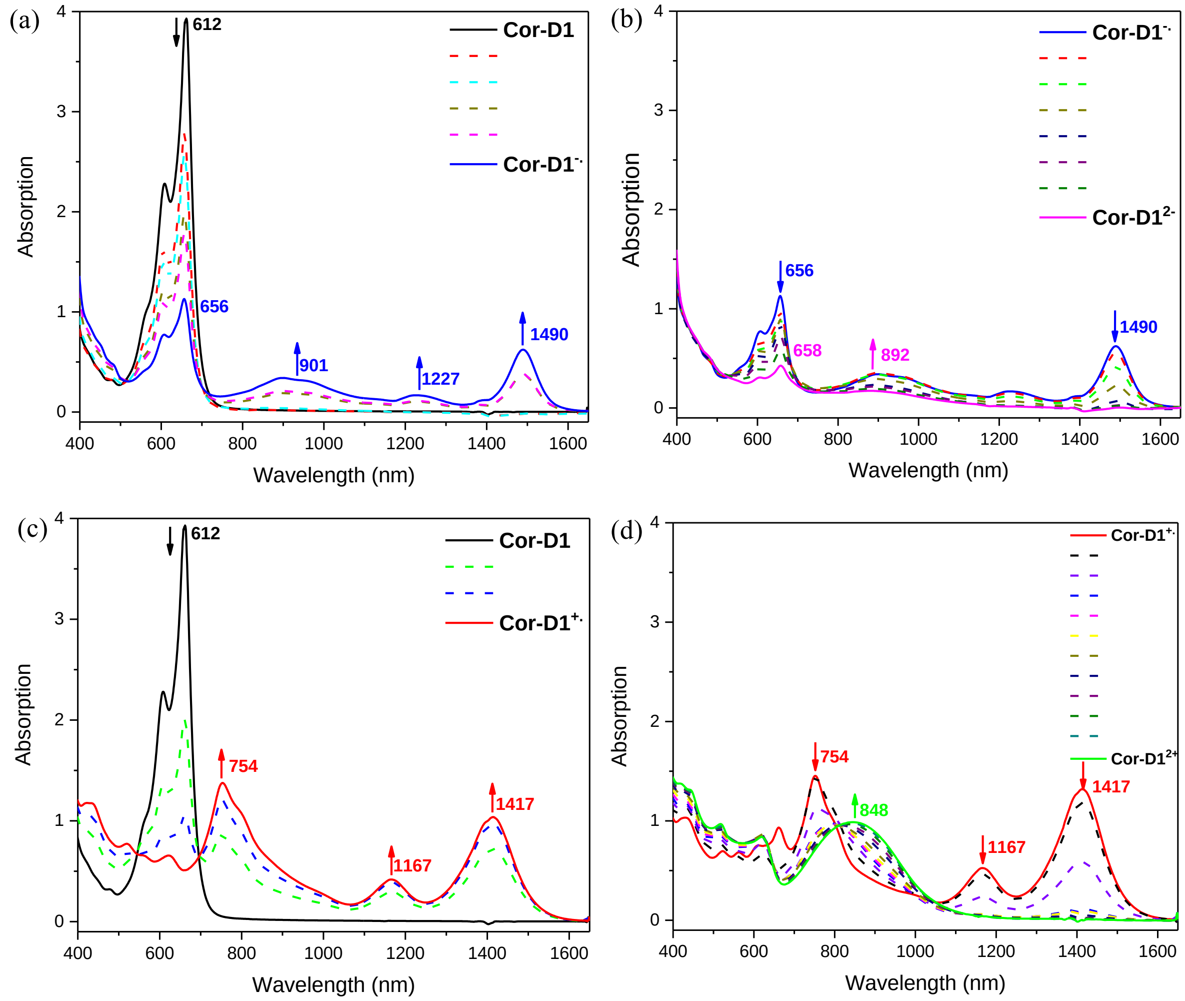

Fig. S11. The change of the UV-vis-NIR absorptuion spectra during the reductive and oxidative titration of **Cor-D1**: (a) from neutral to radical anion, (b) from radical anion to dianion, (c) from neutral to radical cation, and (d) from radical cation to dication. Reductant: Sodium anthracenide (NaAn); oxidant: NO•SbF_6.

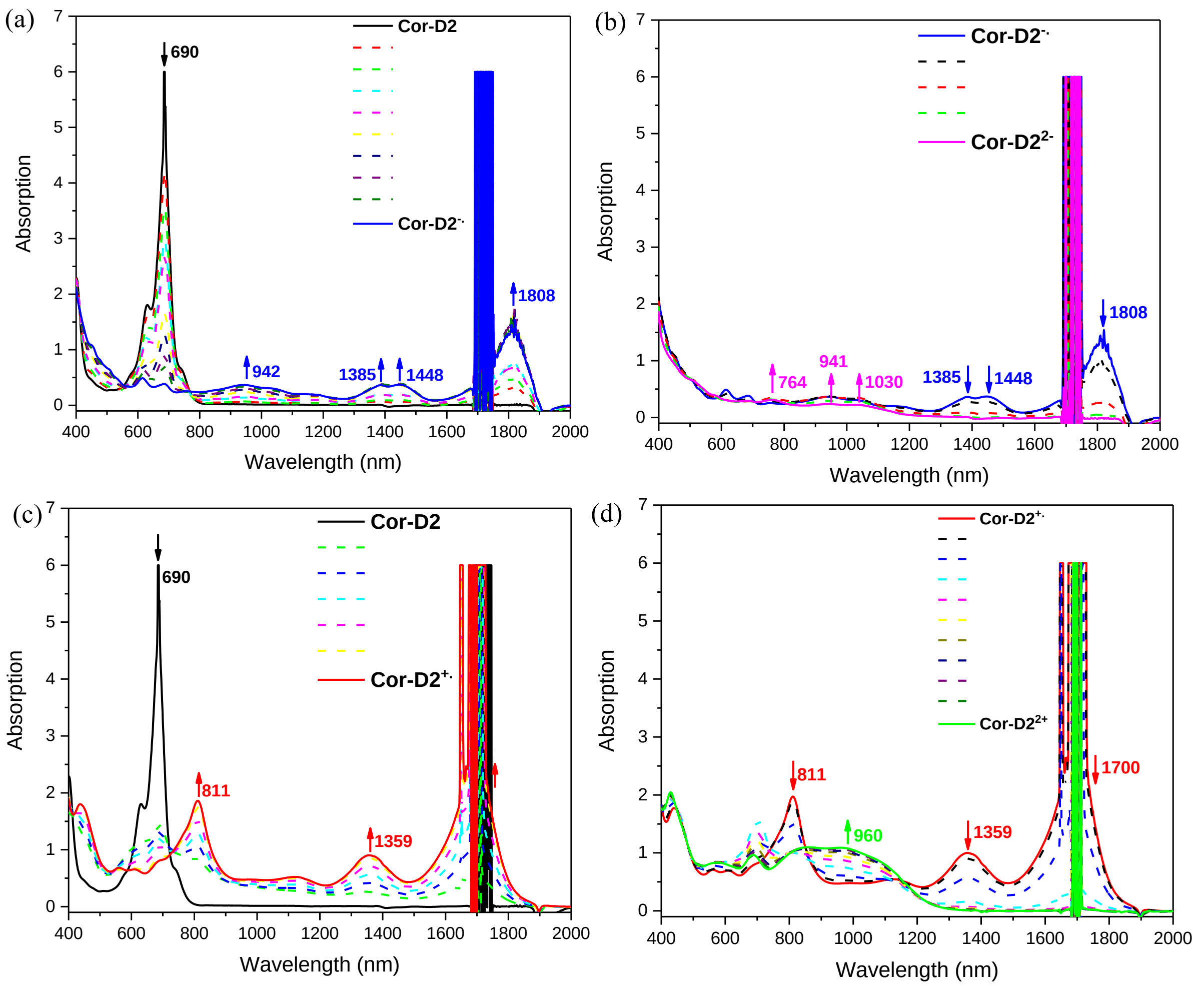

Fig. S12. The change of the UV-vis-NIR absorptuion spectra during the reductive and oxidative titration of **Cor-D2**: (a) from neutral to radical anion, (b) from radical anion to dianion, (c) from neutral to radical cation, (d) from radical cation to dication. Reductant: Sodium anthracenide (NaAn); oxidant: NO•SbF_6.

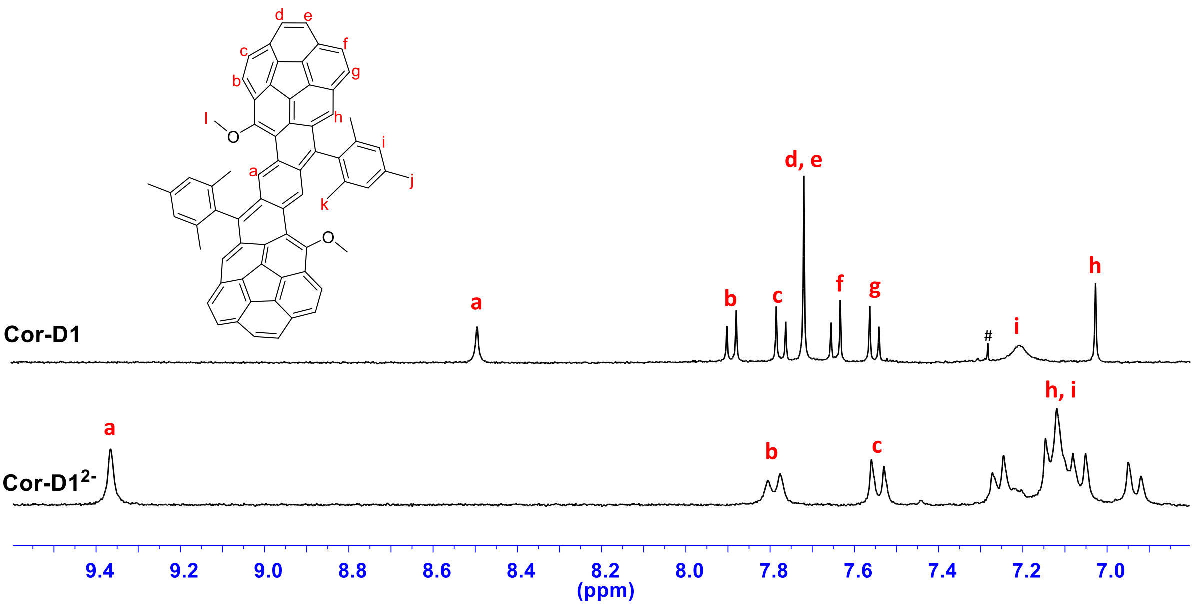

Fig. S13. ^{1}H NMR spectra (aromatic region) of **Cor-D1** (THF-d_8/CS_2 = 2/1, v/v, 400 MHz, rt) and its dianion **Cor-D1^{2-}** (THF-d_8, 300 MHz, rt) by *in situ* reduction with sodium anthracenide. The peak labeled by # comes from impurities in CS_2.

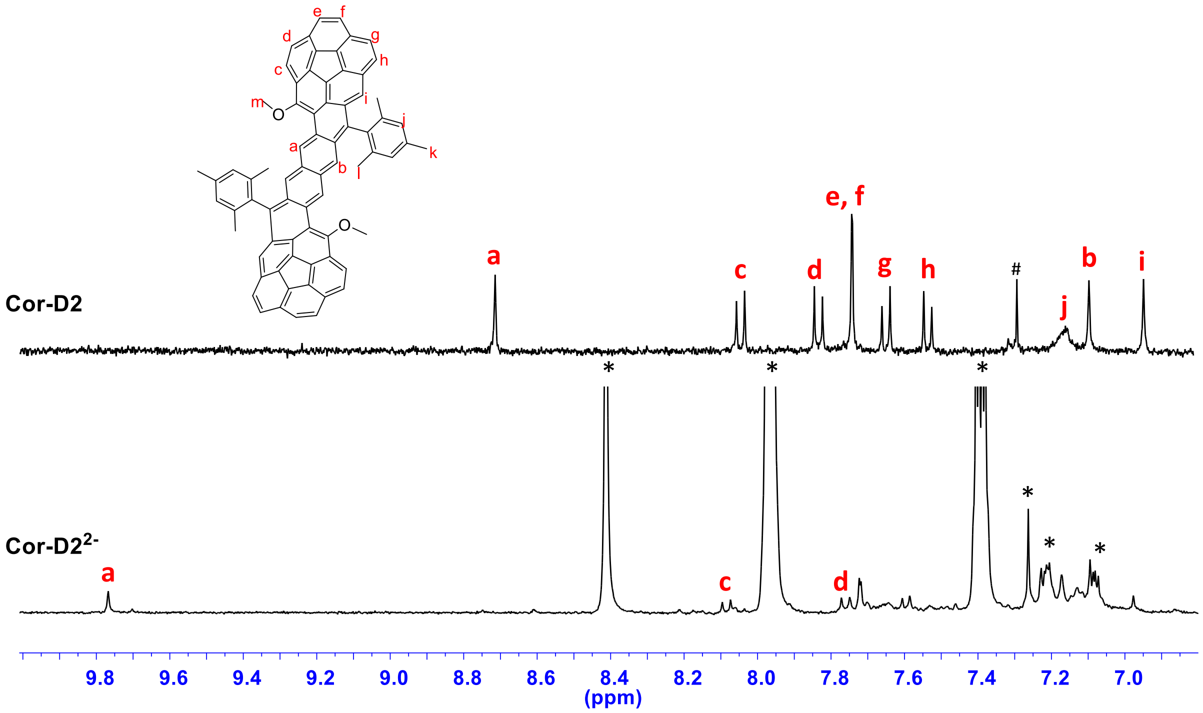

Fig. S14. ^{1}H NMR spectra (aromatic region) of **Cor-D2** (THF-d_8/CS_2 = 2/1, v/v, 400 MHz, rt) and its dianion **Cor-D2^{2-}** (THF-d_8, 300 MHz, rt) by *in situ* reduction with sodium anthracenide. The peak labeled by # comes from impurities in CS_2 and the signals labeled by * are due to the existence of anthracene after chemical reduction. Due to strong aggregation of **Cor-D2** in solution, the ^{1}H NMR spectrum can only be recorded at low concentration. For the dianion, high concentration solution gave broad signals so only the resolved NMR spectrum at very low concentration is shown.

5. DFT calculations

Theoretical calculations were performed with the Gaussian09 program suite.[4] All calculations were carried out using the density functional theory (DFT) method with Becke's three-parameter hybrid exchange functionals and the Lee-Yang-Parr correlation functional (B3LYP) employing the 6-31G(d,p) basis set for all atoms.[5] Time-dependent DFT (TD-DFT) calculations were performed at the (U)B3LYP/6-31G(d,p) level of theory under vacuum. Both the *syn*- and *anti*- isomers were calculated for comparison and they show similar physical properties, so only the *anti*- isomers were considered for other calculations. NOON calculations were done by spin unrestricted UCAM-B3LYP/6-31G(d,p) method based on the X-ray crystallographic structure and the diradical character (y_0) was calculated according to Yamaguchi's scheme: $y_0 = 1 - (2T/(1 + T^2))$, and $T = (n_{\mathrm{HOMO}} - n_{\mathrm{LUMO}})/2$.[6] NICS values were calculated (UB3LYP/6-31G(d,p)) using the standard GIAO procedure (NMR pop=NCSall).[7] AICD plot (B3LYP/6-31G(d,p)) was calculated by using the method developed by Herges based on the optimized ground-state geometries.[8]

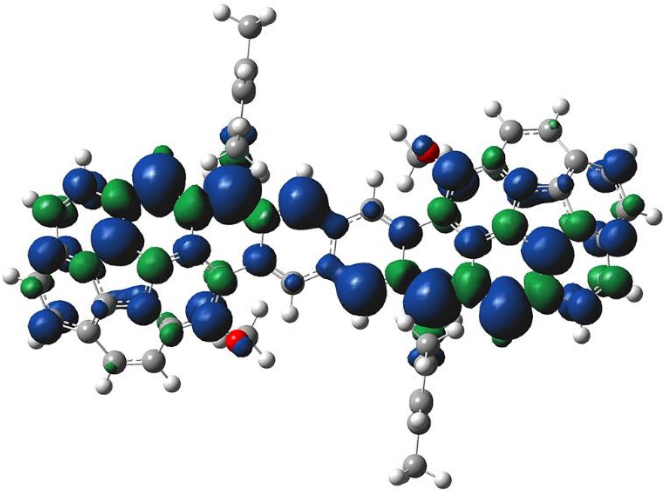

Fig. S15. Calculated spin density distribution map of the triplet biradical of *syn*- **Cor-D2**.

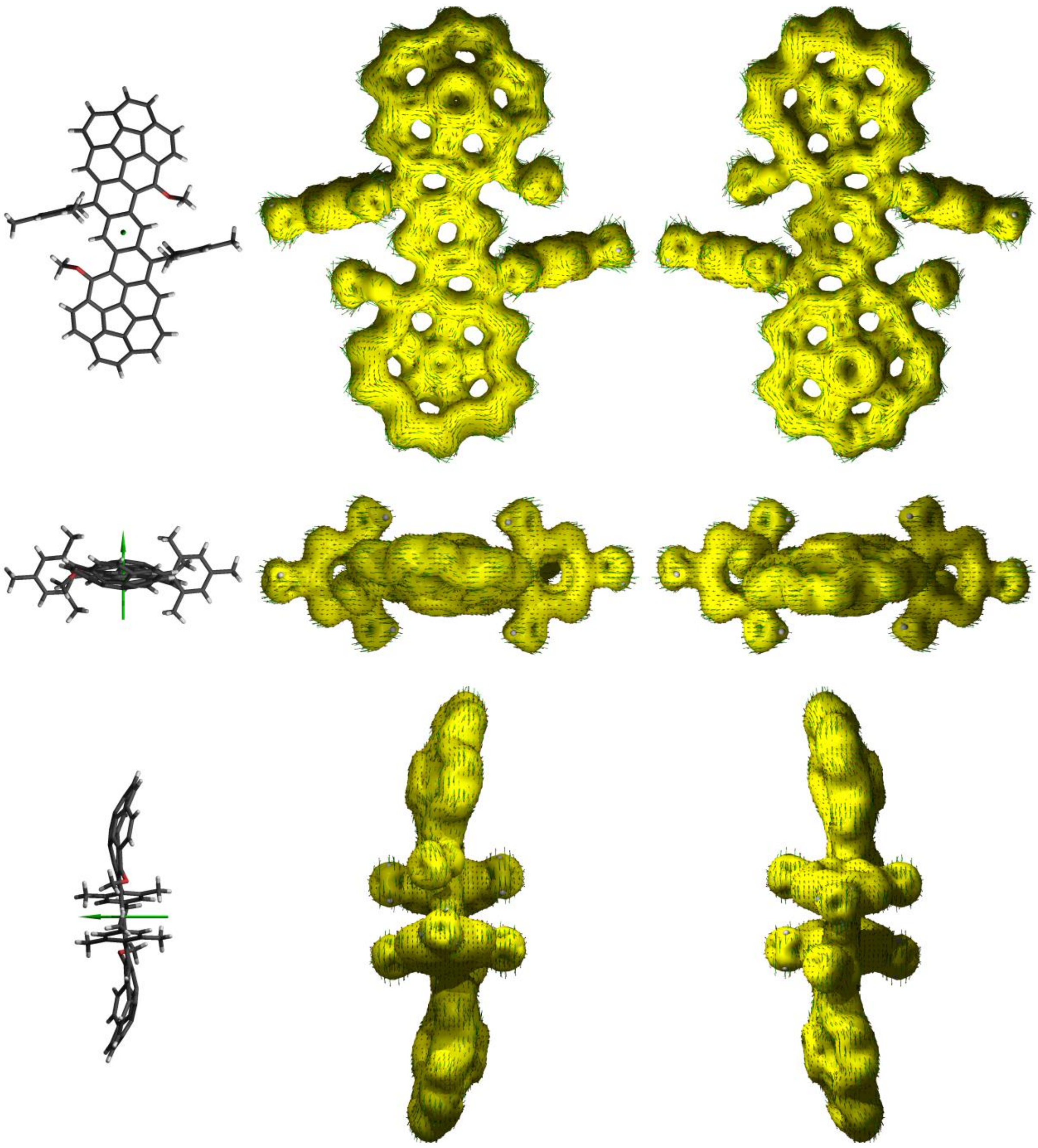

Fig. S16. ACID plots of the *anti*- **Cor-D1** viewed from different angles. The green arrow indicates the magnetic field. Isovalue is 0.02.

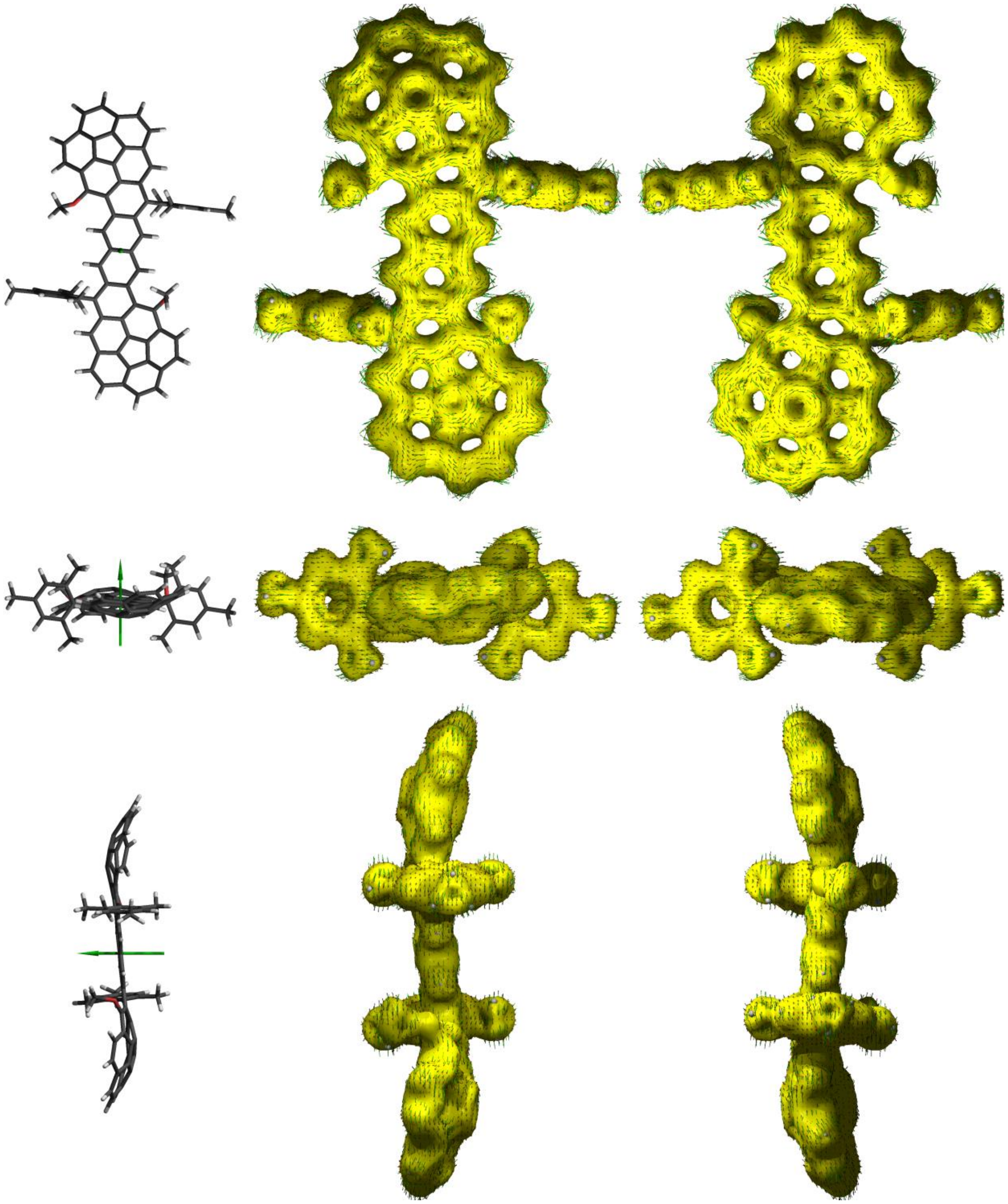

Fig. S17. ACID plots of the *anti*- **Cor-D2** viewed from different angles. The green arrow indicates the magnetic field. Isovalue is 0.02.

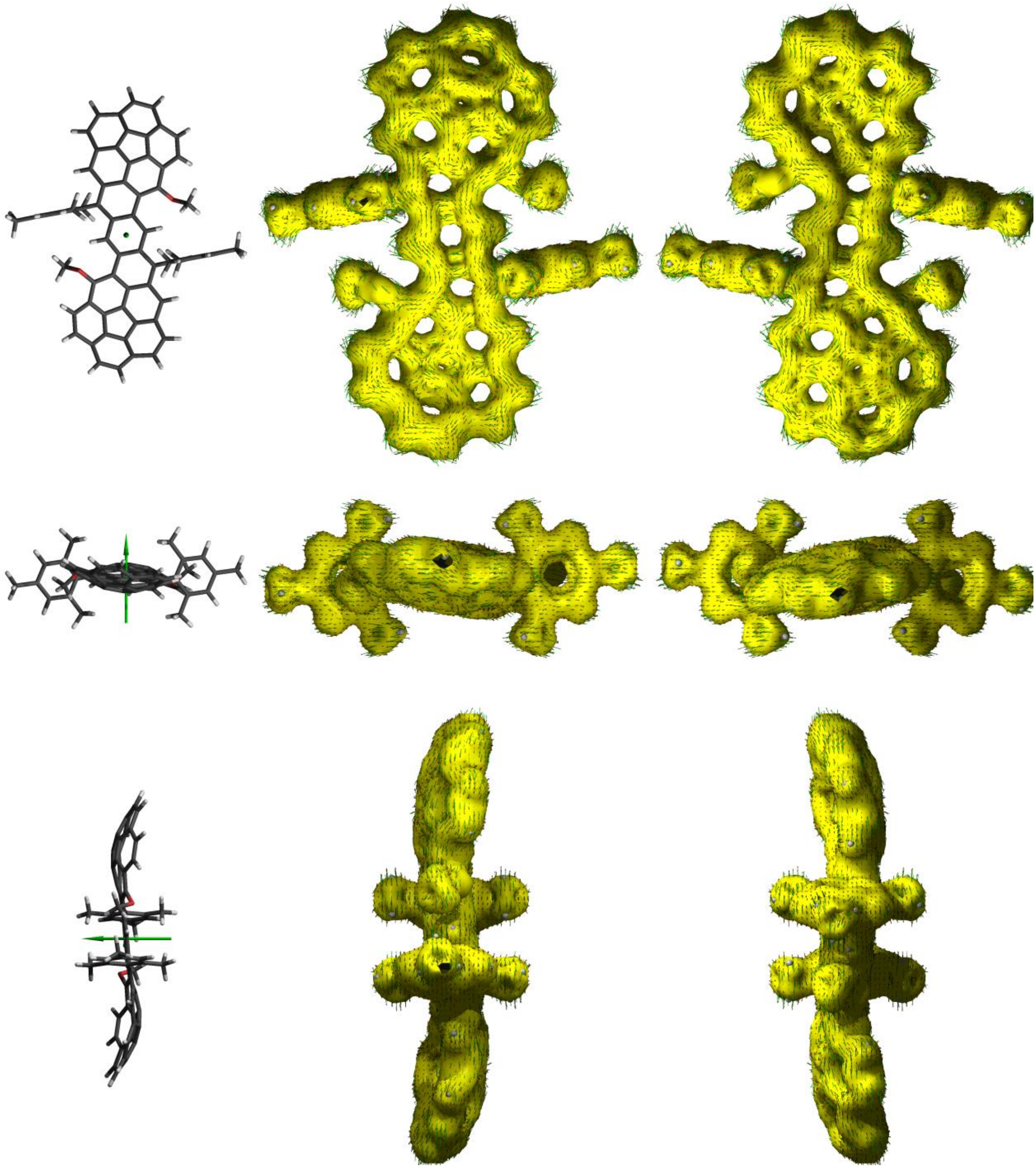

Fig. S18. ACID plots of the *anti*- **Cor-D1^{2-}** viewed from different angles. The green arrow indicates the magnetic field. Isovalue is 0.02.

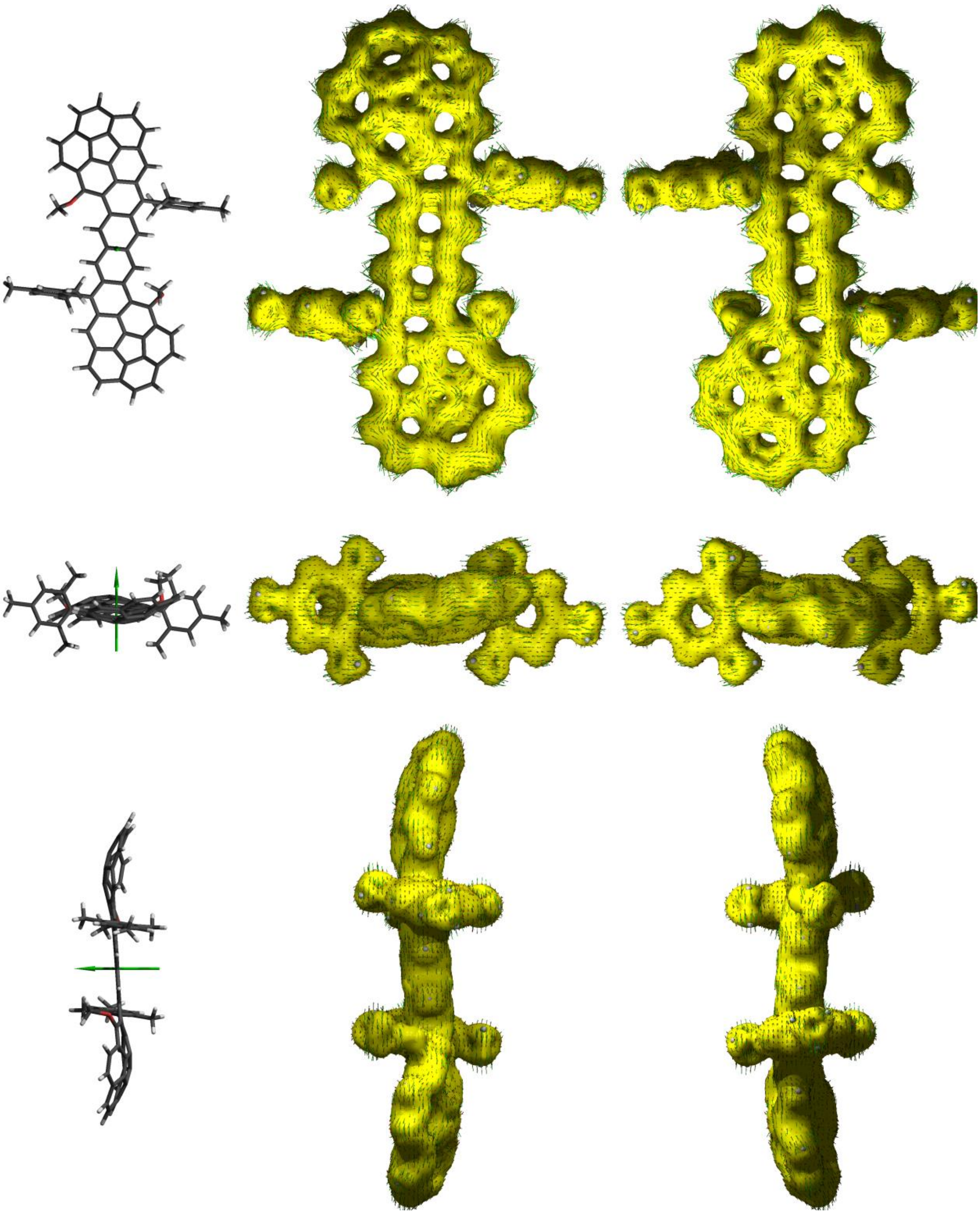

Fig. S19. ACID plots of the *anti*- **Cor-D2^{2-}** viewed from different angles. The green arrow indicates the magnetic field. Isovalue is 0.02.

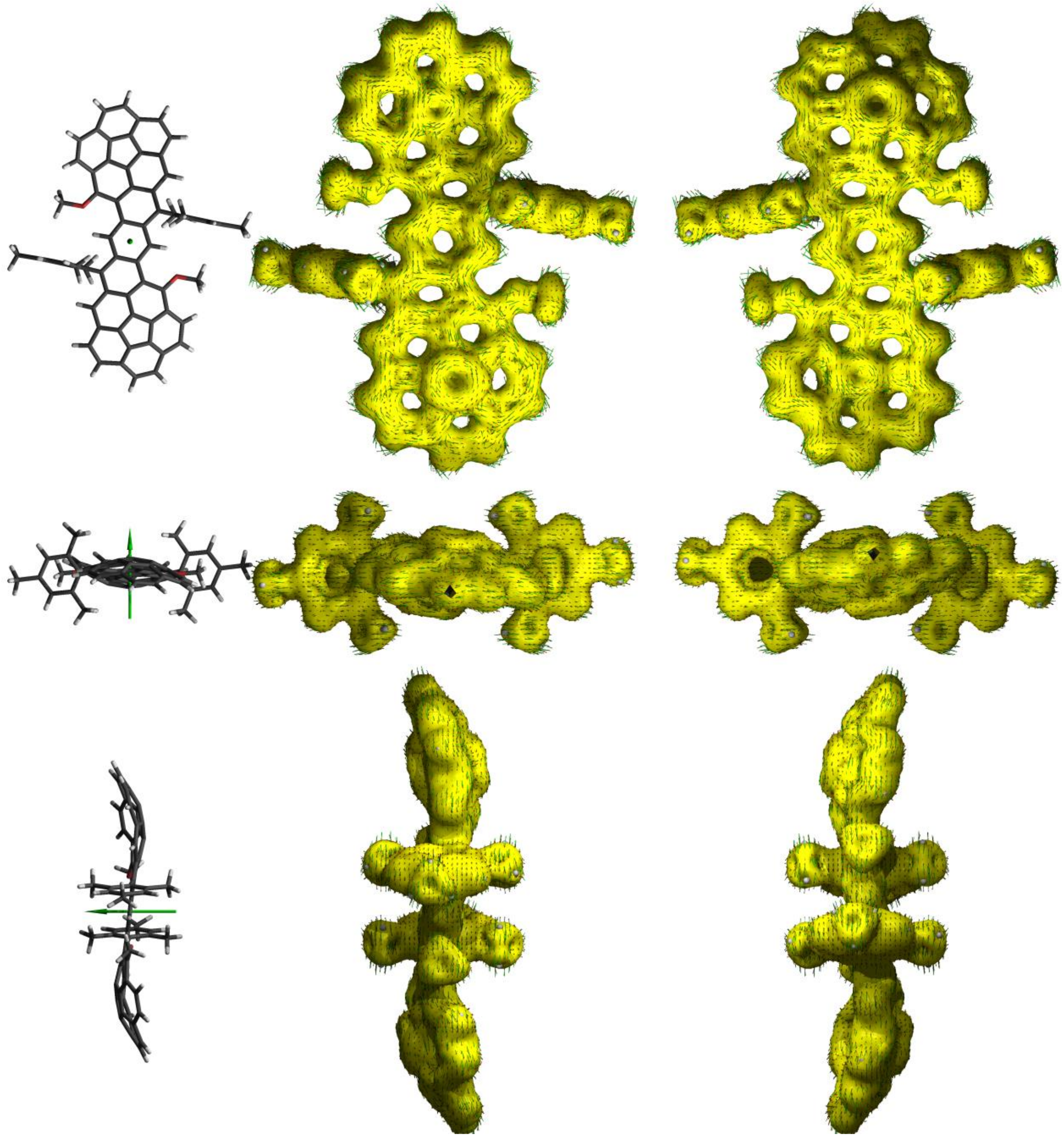

Fig. S20. ACID plots of the *anti*- **Cor-D1^{2+}** viewed from different angles. The green arrow indicates the magnetic field. Isovalue is 0.02.

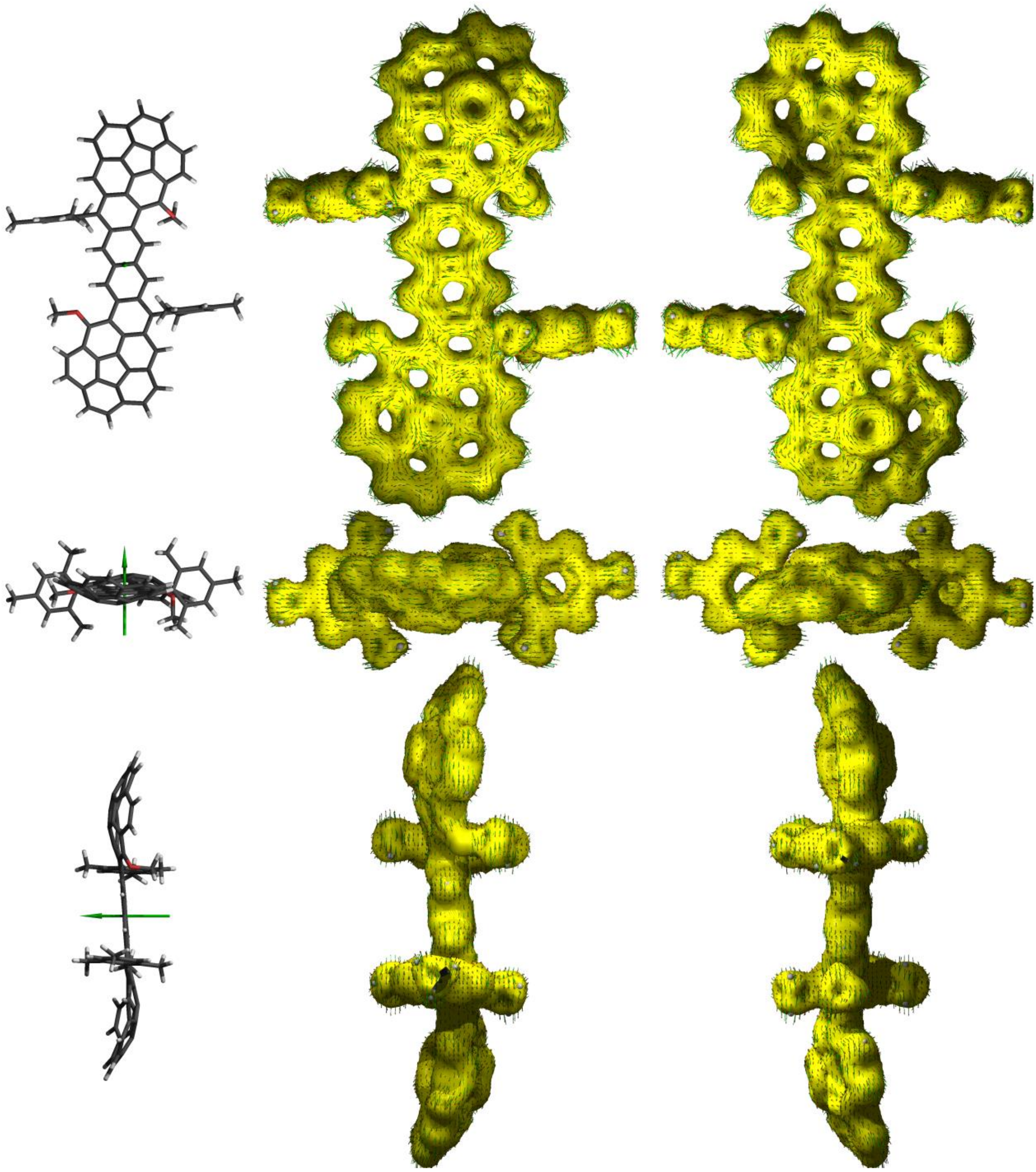

Fig. S21. ACID plots of the *anti*- **Cor-D2**$^{2+}$ viewed from different angles. The green arrow indicates the magnetic field. Isovalue is 0.02.

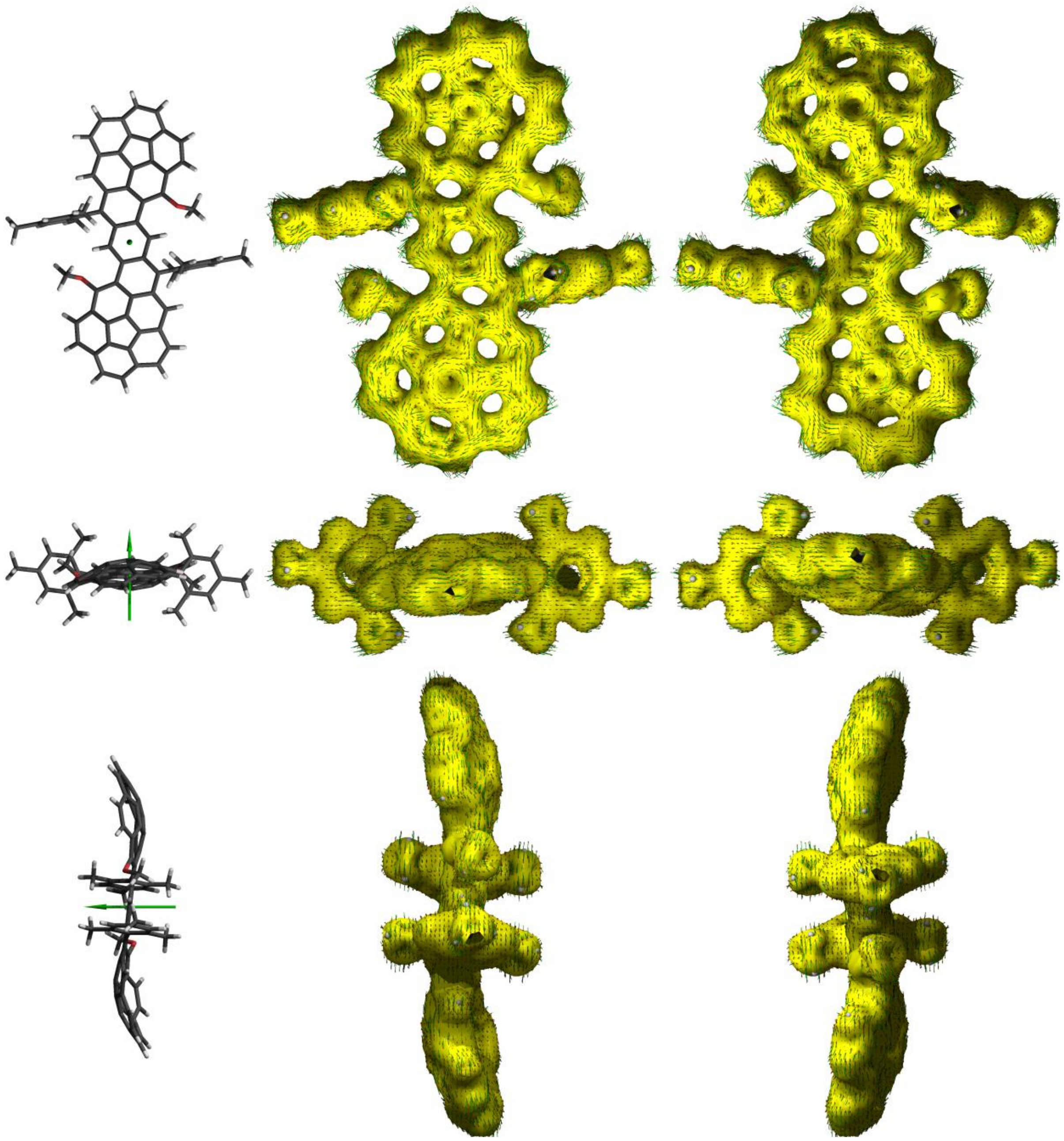

Fig. S22. ACID plots of the *anti*- **Cor-D1$^{-\bullet}$** viewed from different angles. The green arrow indicates the magnetic field. Isovalue is 0.02.

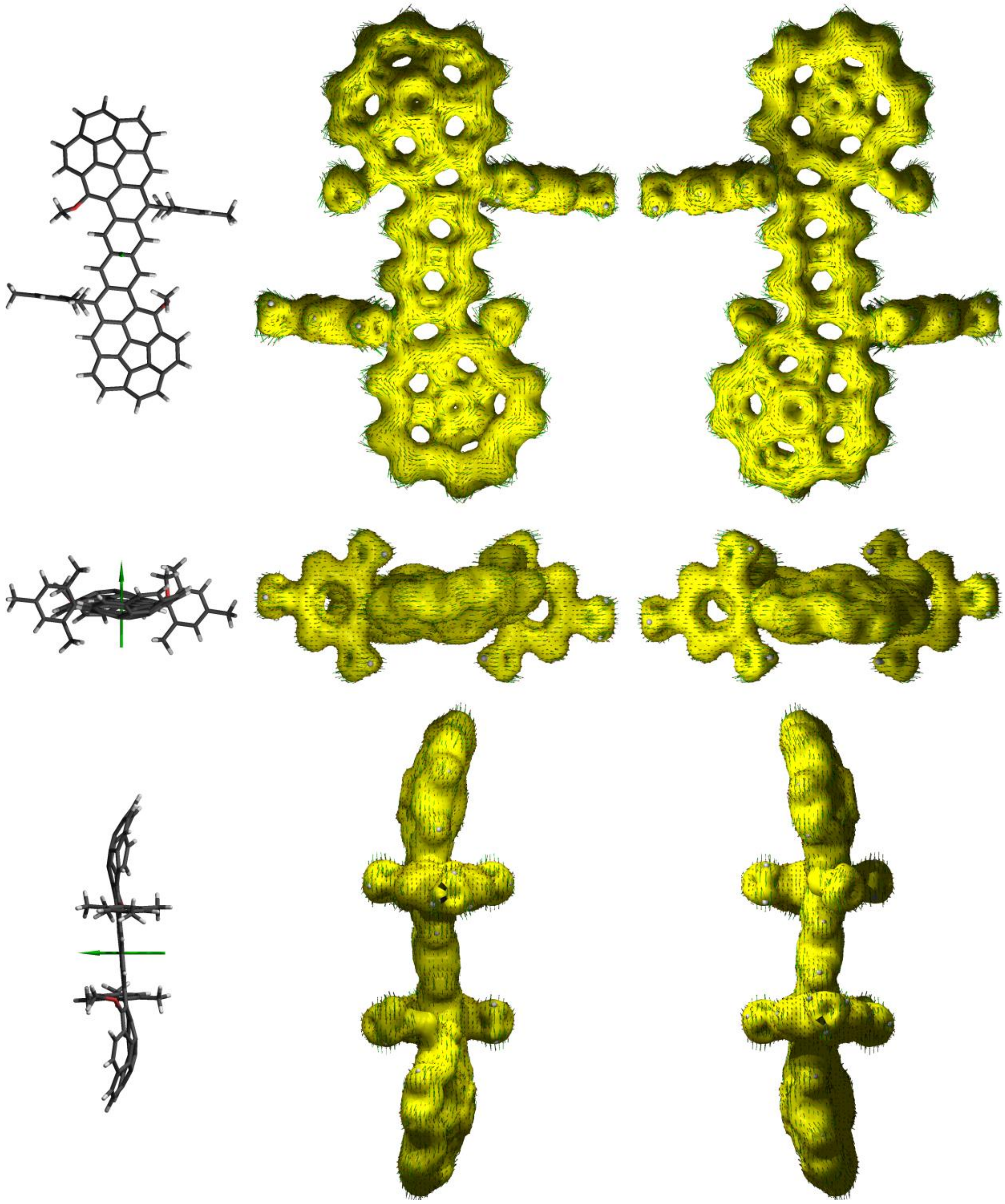

Fig. S23. ACID plots of the *anti*- **Cor-D2$^{-\bullet}$** viewed from different angles. The green arrow indicates the magnetic field. Isovalue is 0.02.

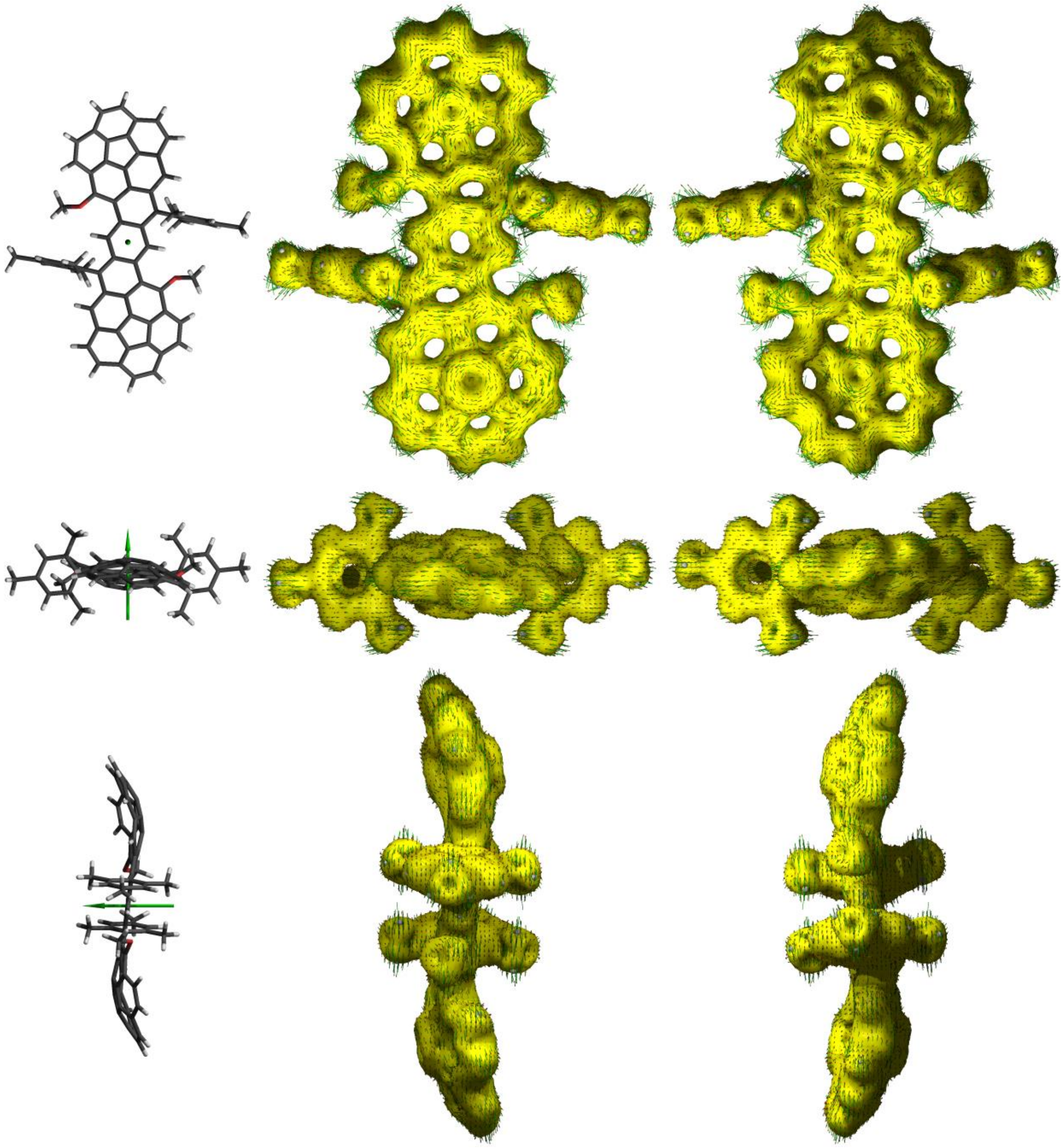

Fig. S24. ACID plots of the *anti*- **Cor-D1**$^{+\bullet}$ viewed from different angles. The green arrow indicates the magnetic field. Isovalue is 0.02.

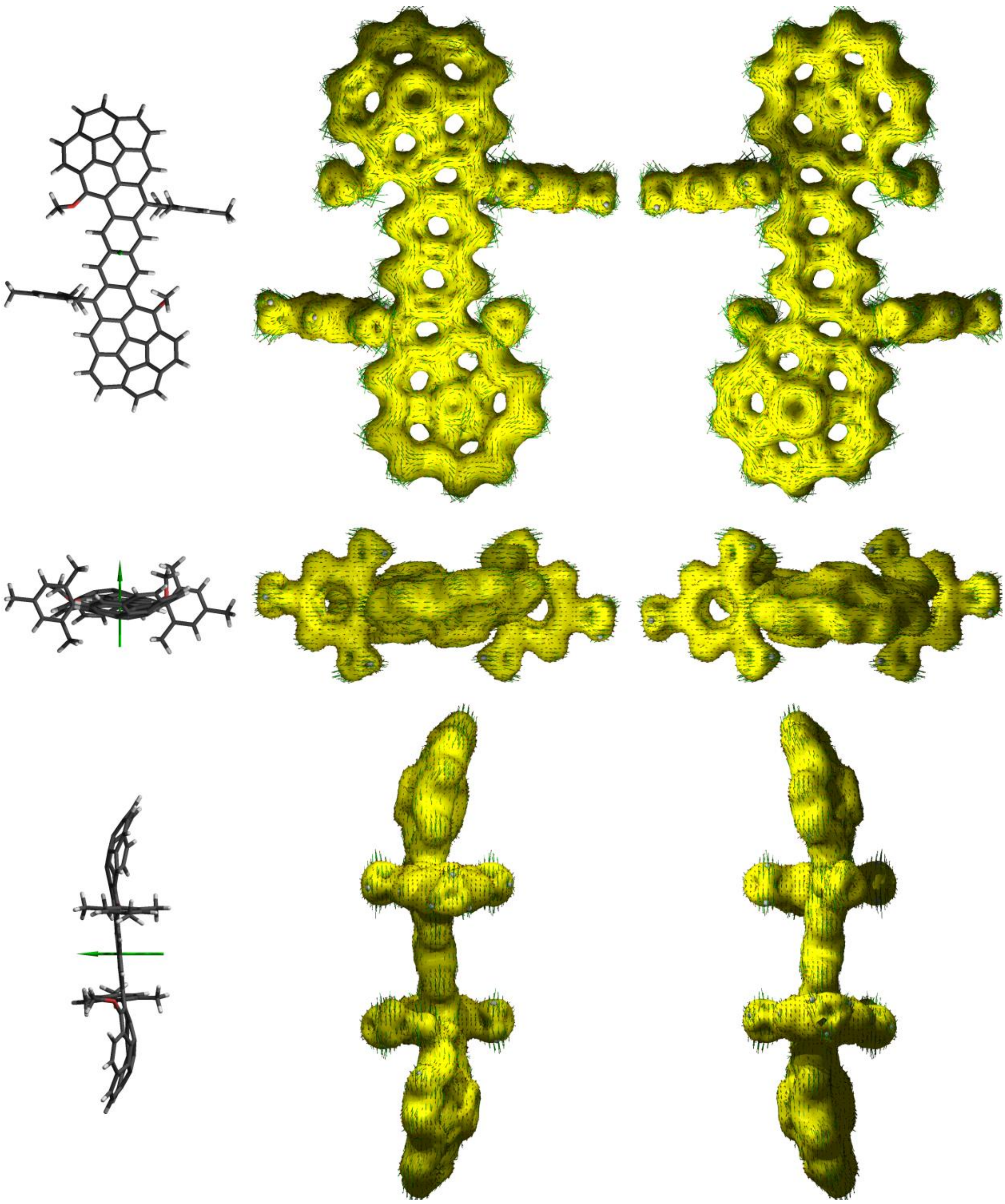

Fig. S25. ACID plots of the *anti*- **Cor-D2**$^{+\bullet}$ viewed from different angles. The green arrow indicates the magnetic field. Isovalue is 0.02.

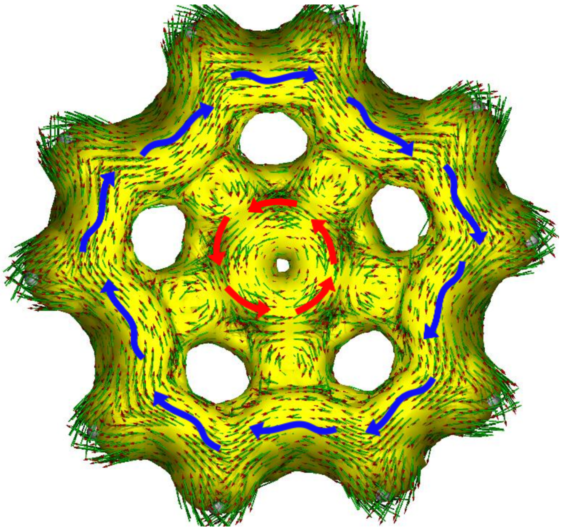

Fig. S26. ACID plots of parent corannulene. The magnetic field is point out through the paper. The blue and red arrows indicate the diamagnetic and paramagnetic ring current flow, respectively. Isovalue is 0.03.

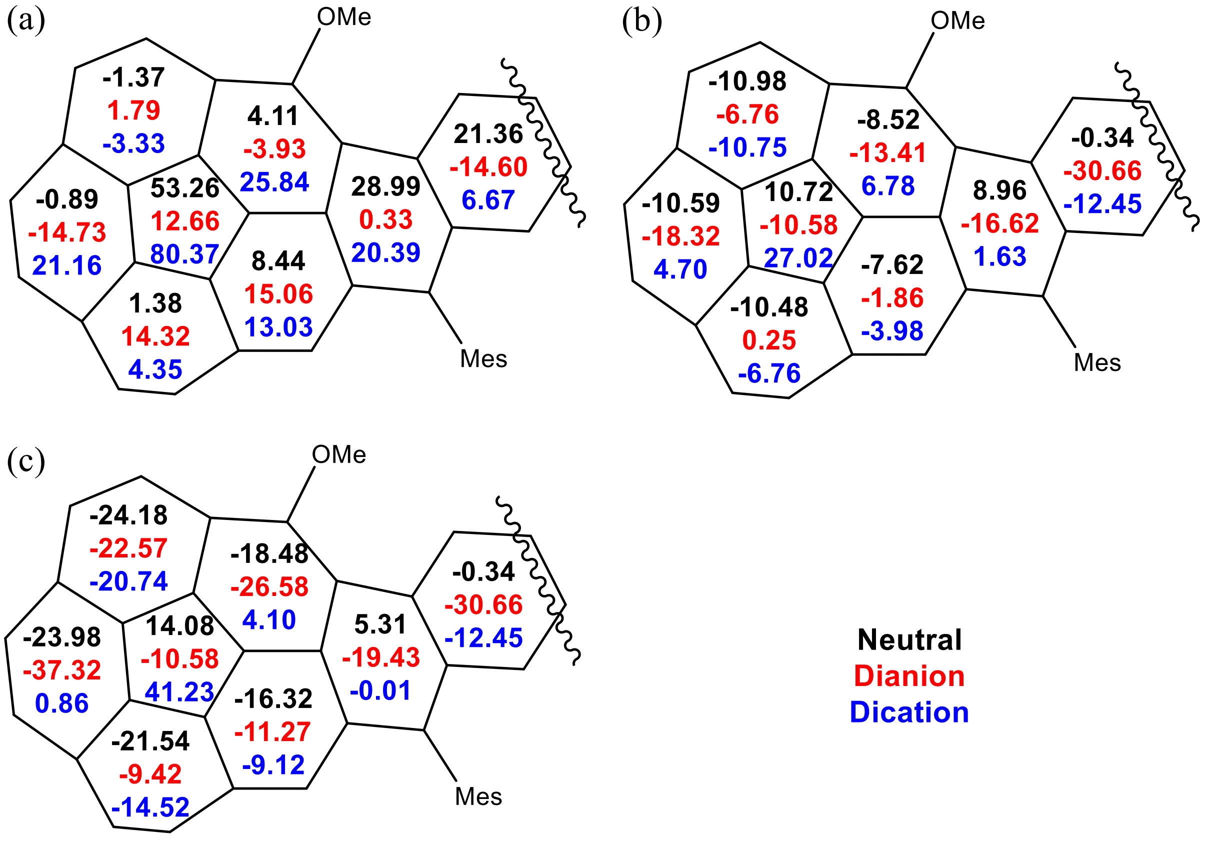

Fig. S27. Calculated (a) NICS(0)zz values, (b) NICS(1)zz values, (c) NICS(-1)zz values of the neutral, dianion and dication of the *anti*- **Cor-D1** at B3LYP/6-31G(d,p) level.

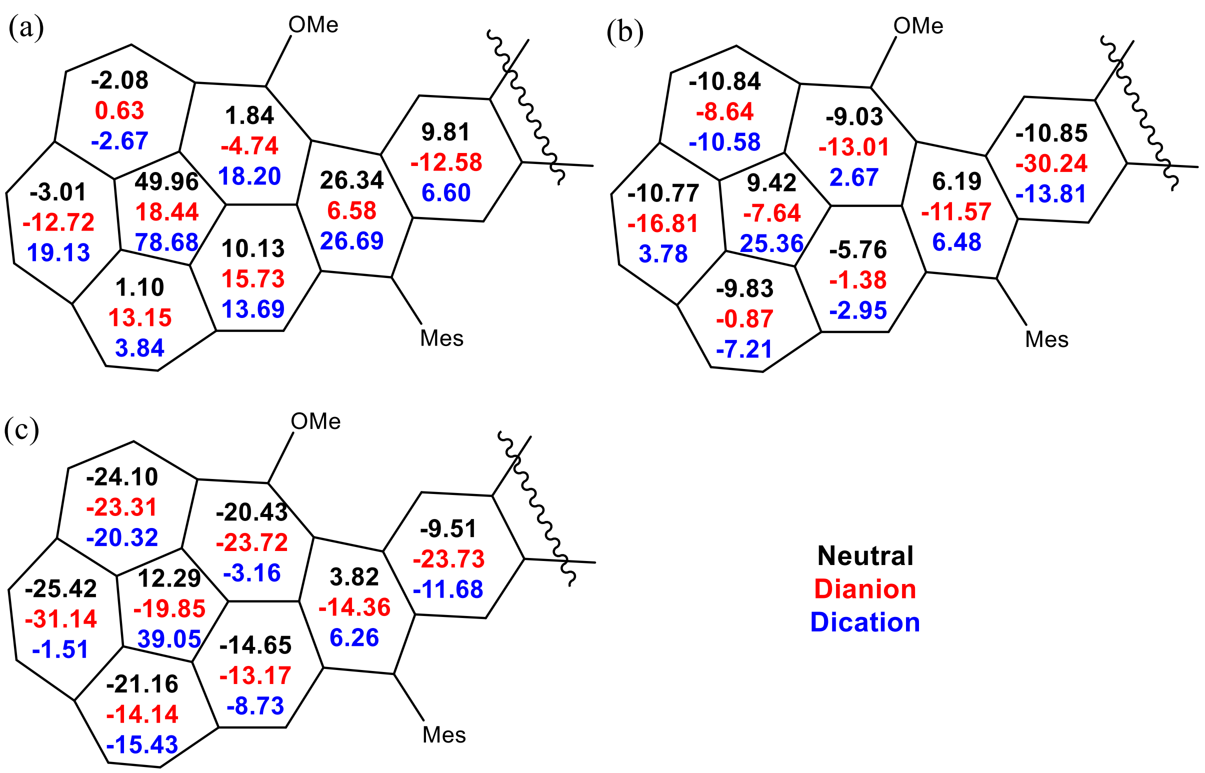

Fig. S28. Calculated (a) NICS(0)zz values, (b) NICS(1)zz values, (c) NICS(-1)zz values of the neutral, dianion and dication of the *anti*- **Cor-D2** at B3LYP/6-31G(d,p) level.

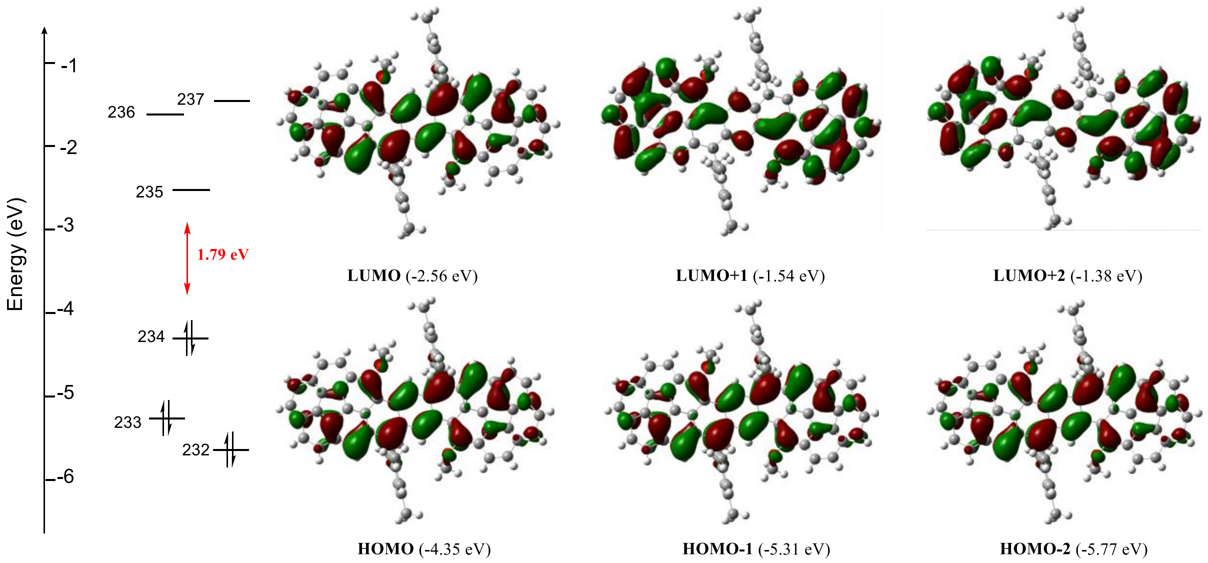

Fig. S29. Frontier molecular orbital profiles and energy diagram of the *anti*- **Cor-D1** obtained by B3LYP/6-31G(d,p) level calculation.

Table S3. Selected TD-DFT (B3LYP/6-31G(d)) calculated energies, oscillator strength and compositions of major electronic transitions of the *anti*- **Cor-D1**.

Wavelength (nm)	Osc. Strength	Major contribs
670.2527	1.0459	HOMO->LUMO (100%)
563.74	0	H-1->LUMO (91%)
520.7633	0	HOMO->L+1 (89%)
495.1411	0.0159	H-2->LUMO (11%), HOMO->L+2 (86%)
466.6817	0	HOMO->L+3 (83%)
455.2353	0.0419	H-2->LUMO (83%)
422.4736	0	H-5->LUMO (38%), H-3->LUMO (48%)
421.8842	0	H-5->LUMO (53%), H-3->LUMO (39%)
420.5391	0.0062	H-4->LUMO (95%)
403.6047	0.0003	H-6->LUMO (76%)
403.5653	0.0226	H-8->LUMO (62%), H-7->LUMO (27%)
402.0342	0.0506	H-8->LUMO (30%), H-7->LUMO (59%)
399.5596	0	H-9->LUMO (93%)
387.8236	0.0027	H-10->LUMO (92%)
385.3526	0	H-11->LUMO (93%)
380.5271	0.0602	HOMO->L+4 (83%)
371.5413	0.3426	H-1->L+1 (88%)
370.3538	0	HOMO->L+5 (91%)

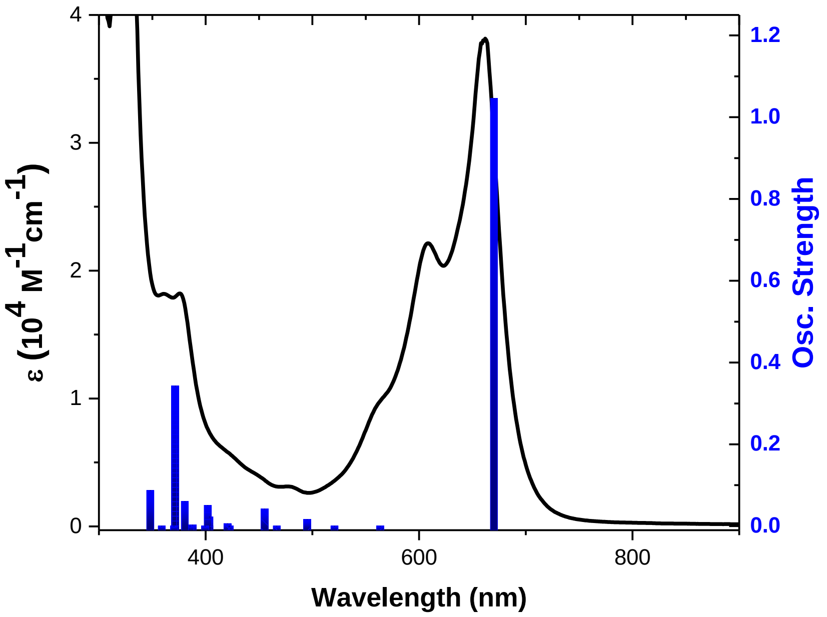

Fig. S30. Calculated stick spectrum (B3LYP/6-31G(d,p)) of the *anti*- **Cor-D1** along with the experimental spectrum.

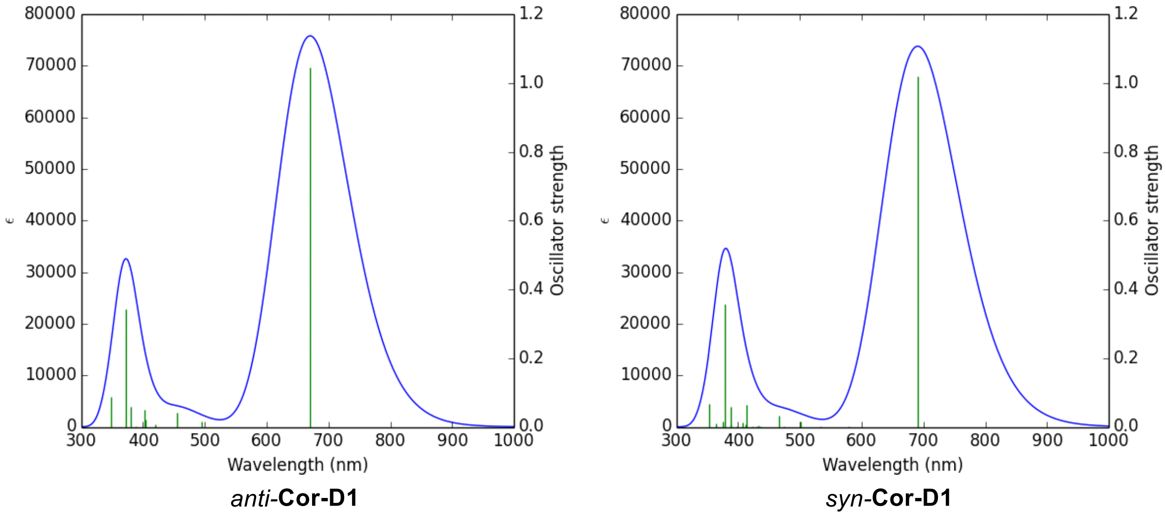

Figure S31. Calculated (B3LYP/6-31G(d,p)) electronic absorption spectra of the *anti*- and *syn*- isomers of **Cor-D1**.

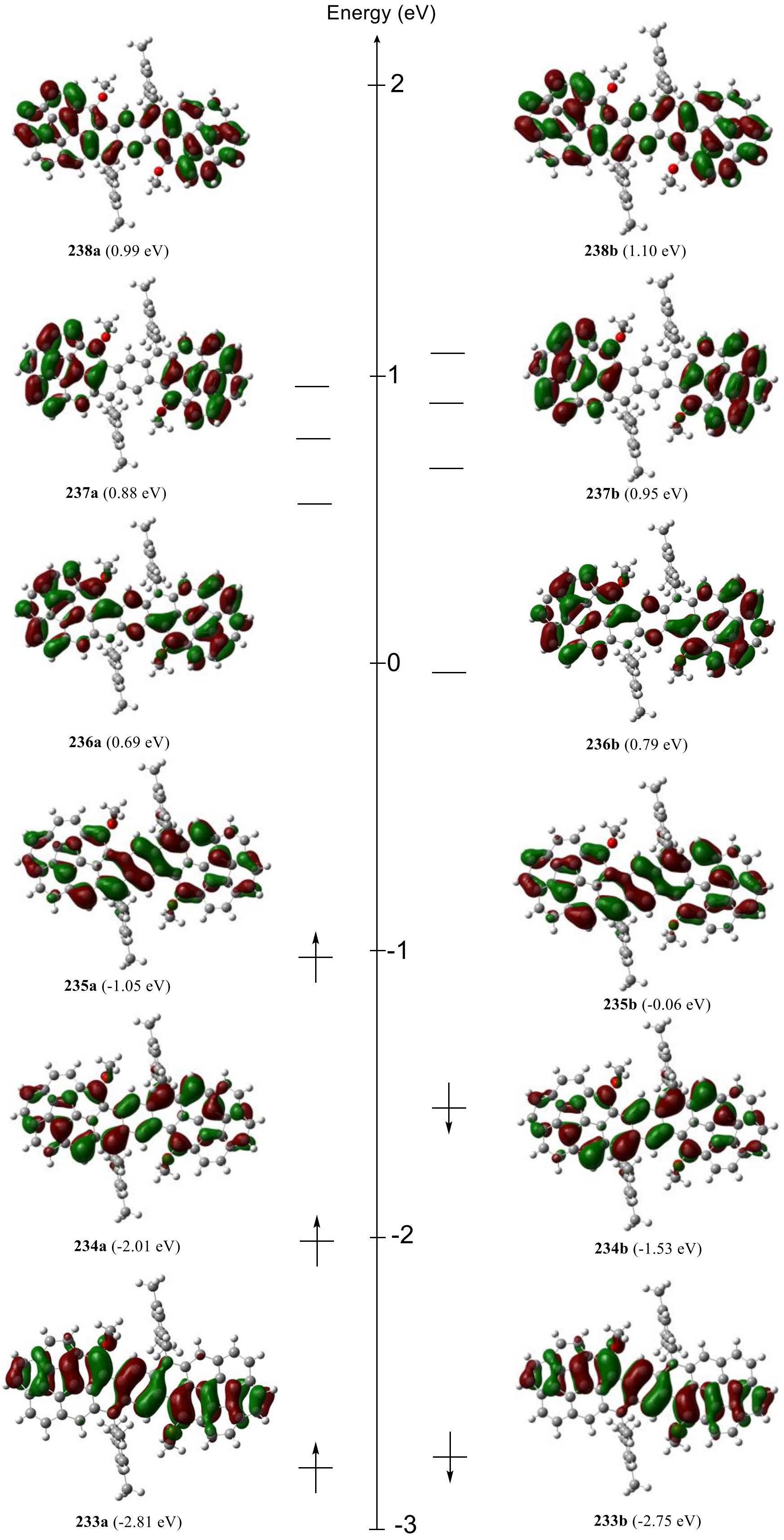

Fig. S32. Frontier molecular orbital profiles and energy diagram of the *anti*- **Cor-D1**$^{-\cdot}$ obtained by UB3LYP/6-31G(d,p) level calculation.

Table S4. Selected TD-DFT (UB3LYP/6-31G(d)) calculated energies, oscillator strength and compositions of major electronic transitions of the *anti*- **Cor-D1**$^{-\cdot}$.

Wavelength (nm)	Osc. Strength	Major contribs
1208.297	0.274	HOMO(B)->LUMO(B) (89%)
1045.303	0.0175	HOMO(A)->LUMO(A) (93%)
936.9254	0	HOMO(A)->L+1(A) (86%)
800.719	0.4655	HOMO(A)->L+2(A) (84%)
742.86	0	HOMO(A)->L+3(A) (12%), HOMO(B)->L+1(B) (54%)
653.4381	0	HOMO(A)->L+3(A) (36%), HOMO(B)->L+1(B) (27%), HOMO(B)->L+3(B) (26%)
643.5678	0.041	HOMO(B)->L+2(B) (87%)
623.0632	0	H-1(B)->LUMO(B) (70%)
571.1675	0	HOMO(A)->L+3(A) (38%), HOMO(B)->L+3(B) (47%)
555.6801	0	H-1(A)->LUMO(A) (76%), H-1(B)->LUMO(B) (11%)
555.2073	0.052	H-1(A)->L+1(A) (16%), HOMO(A)->L+4(A) (71%)
531.9347	0.0056	H-2(A)->LUMO(A) (16%), H-1(A)->L+1(A) (29%), H-2(B)->LUMO(B) (14%), H-1(B)->L+1(B) (13%)
505.8273	0	H-1(A)->L+2(A) (54%), H-3(B)->LUMO(B) (25%), HOMO(B)->L+3(B) (10%)
496.4298	0.1083	H-1(A)->L+1(A) (40%), HOMO(A)->L+4(A) (20%), H-1(B)->L+1(B) (10%)
491.9193	0.0341	H-1(A)->L+3(A) (11%), HOMO(B)->L+4(B) (63%)
479.6817	0	HOMO(A)->L+5(A) (96%)
471.5271	0.0032	HOMO(A)->L+6(A) (94%)
466.2255	0	H-3(A)->LUMO(A) (10%), H-2(A)->L+1(A) (15%), H-1(A)->L+2(A) (15%), H-3(B)->LUMO(B) (16%), H-1(B)->L+2(B) (11%)

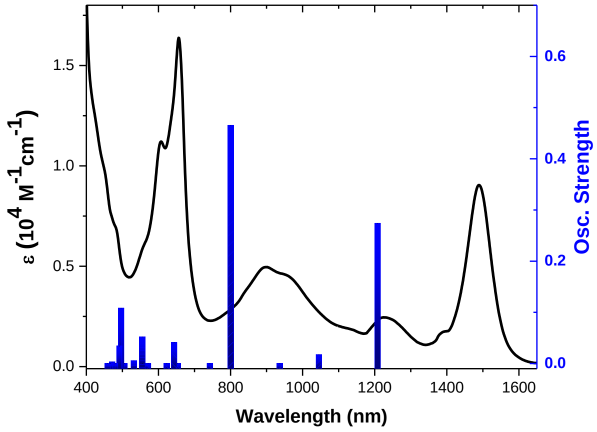

Fig. S33. Calculated stick spectrum (UB3LYP/6-31G*) of the *anti*- **Cor-D1**$^{-\cdot}$ along with the experimental spectrum.

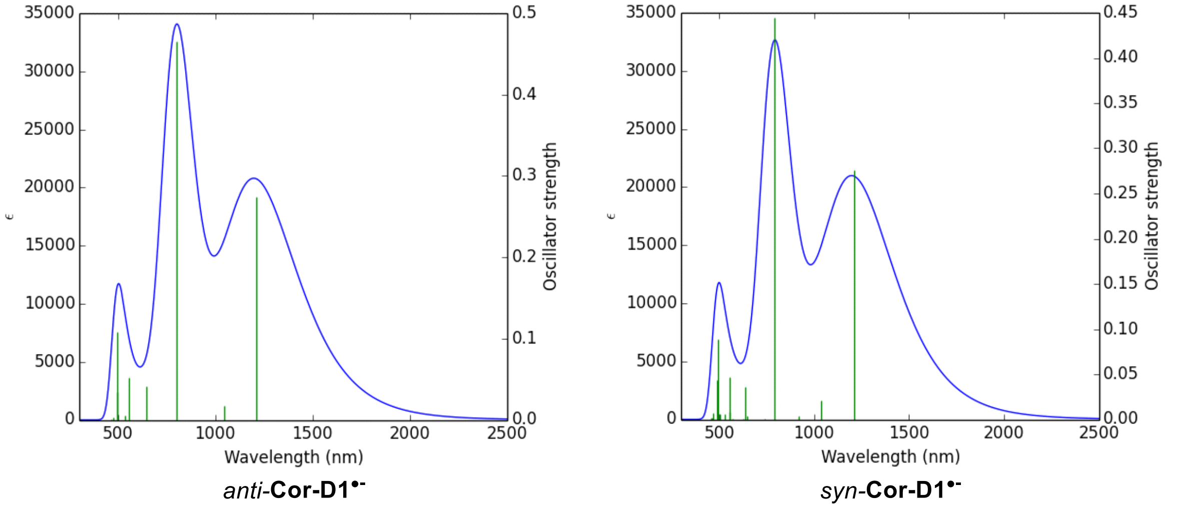

Figure S34. Calculated (UB3LYP/6-31G(d,p)) electronic absorption spectra of the *anti*- and *syn*- isomers of **Cor-D1**$^{\bullet-}$.

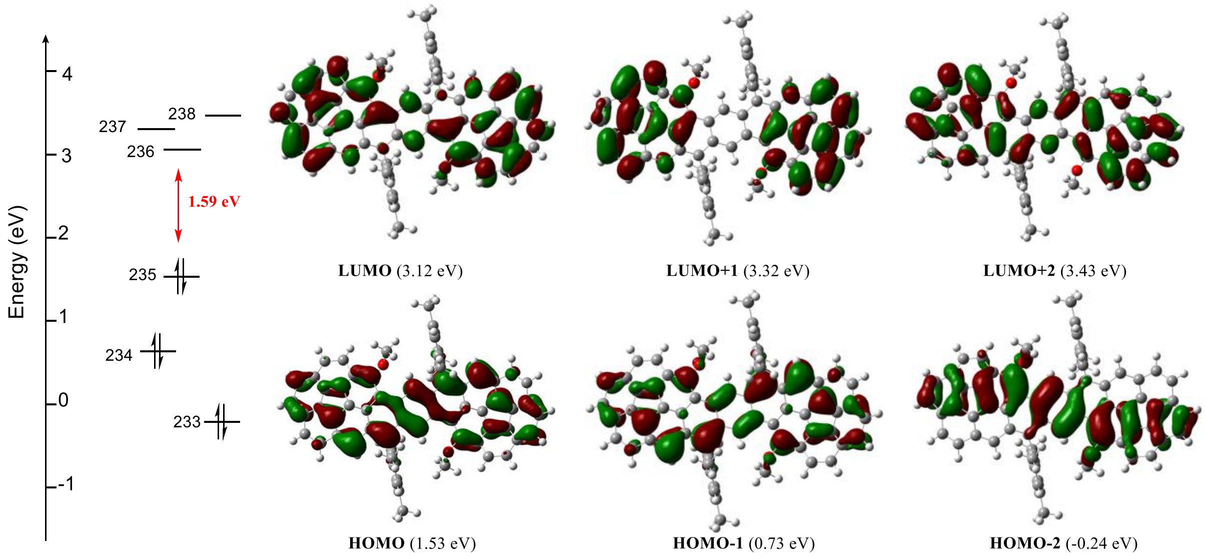

Fig. S35. Frontier molecular orbital profiles and energy diagram of the *anti*- **Cor-D1**$^{2-}$ obtained by B3LYP/6-31G(d,p) level calculation.

Table S5. Selected TD-DFT (B3LYP/6-31G(d)) calculated energies, oscillator strength and compositions of major electronic transitions of the *anti*- **Cor-D1**$^{2-}$.

Wavelength (nm)	Osc. Strength	Major contribs
1039.693	0.0256	HOMO->LUMO (95%)
935.0176	0	HOMO->L+1 (97%)
740.5085	0.5883	HOMO->L+2 (92%)
627.8909	0	H-1->LUMO (60%), HOMO->L+3 (36%)
616.6484	0	H-1->LUMO (36%), HOMO->L+3 (53%)
579.9847	0.0247	H-1->L+1 (79%), HOMO->L+4 (13%)
563.9195	0.0393	HOMO->L+4 (75%), HOMO->L+6 (14%)

562.8698	0.0001	HOMO->L+5 (86%)
534.5031	0.1064	HOMO->L+6 (74%)
527.9032	0	H-1->L+2 (83%)
520.1953	0	HOMO->L+7 (93%)
507.1723	0.0769	HOMO->L+8 (90%)
454.6677	0.0634	H-1->L+3 (92%)
436.8687	0.0102	HOMO->L+9 (83%)
431.3364	0	HOMO->L+10 (78%)
418.9051	0.6217	H-2->LUMO (83%)
411.9869	0	H-1->L+4 (88%)
411.2899	0.0066	H-1->L+5 (93%)

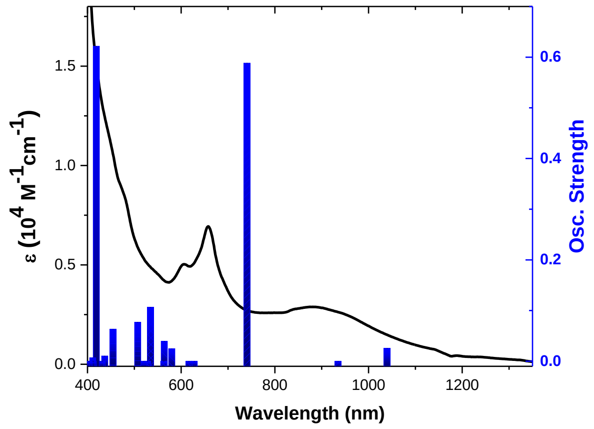

Fig. S36. Calculated stick spectrum (B3LYP/6-31G*) of the *anti*- **Cor-D1^{2-}** along with the experimental spectrum.

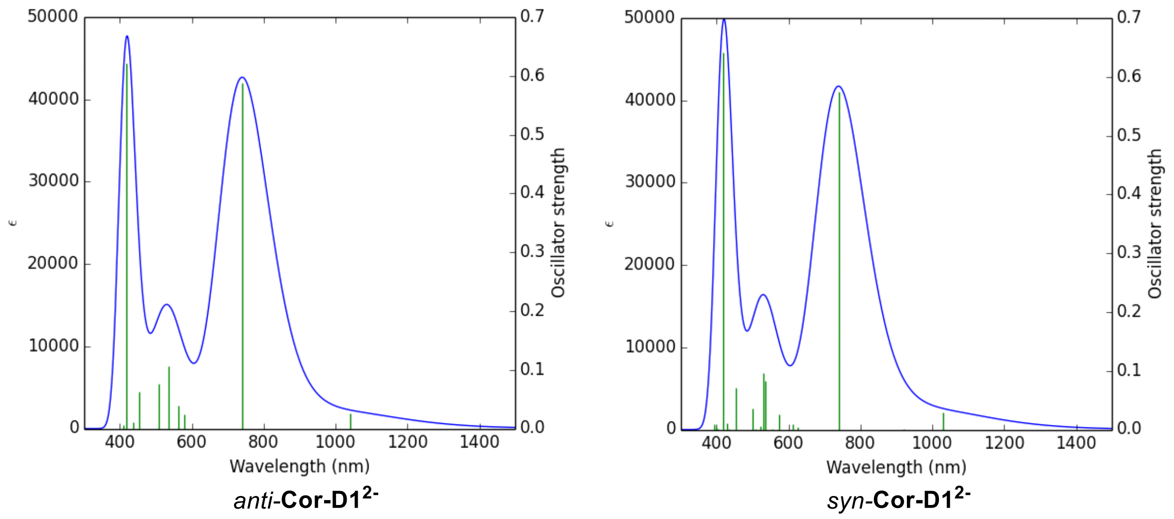

Figure S37. Calculated (B3LYP/6-31G(d,p)) electronic absorption spectra of the *anti*- and *syn*- isomers of **Cor-D1^{2-}**.

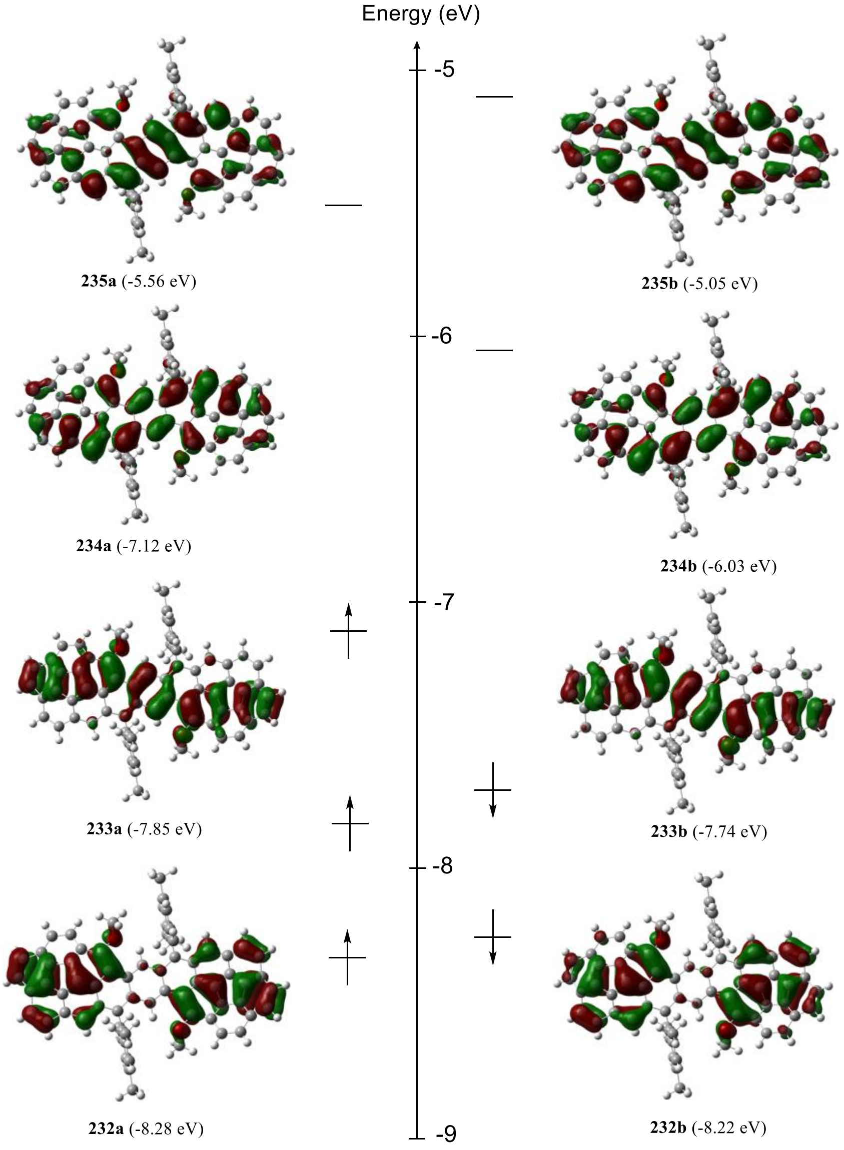

Fig. S38. Frontier molecular orbital profiles and energy diagram of the *anti*- **Cor-D1**$^{+\cdot}$ obtained by UB3LYP/6-31G(d,p) level calculation.

Table S6. Selected TD-DFT (UB3LYP/6-31G(d)) calculated energies, oscillator strength and compositions of major electronic transitions of the *anti*- **Cor-D1**$^{+\cdot}$

Wavelength (nm)	Osc. Strength	Major contribs
1161.11	0.263	HOMO(A)->LUMO(A) (74%), HOMO(B)->LUMO(B) (18%)
1119.589	0.0395	HOMO(A)->LUMO(A) (17%), HOMO(B)->LUMO(B) (79%)
863.2134	0	H-1(A)->LUMO(A) (32%), H-1(B)->LUMO(B) (50%), HOMO(B)->L+1(B) (11%)
748.9178	0	H-1(A)->LUMO(A) (50%), H-1(B)->LUMO(B) (35%)
732.286	0.0189	H-4(B)->LUMO(B) (29%), H-2(B)->LUMO(B) (57%)
726.4507	0.0001	H-3(B)->LUMO(B) (84%)
725.473	0.0288	H-4(B)->LUMO(B) (48%), H-2(B)->LUMO(B) (34%)
681.6016	0	H-5(B)->LUMO(B) (85%)

669.7458	0	H-6(B)->LUMO(B) (95%)
664.1134	0.402	H-7(B)->LUMO(B) (65%), H-4(B)->LUMO(B) (13%)
652.4752	0.005	H-8(B)->LUMO(B) (96%)
617.4469	0	H-9(B)->LUMO(B) (97%)
617.2317	0.0007	H-10(B)->LUMO(B) (98%)
608.537	0	H-1(A)->LUMO(A) (12%), HOMO(B)->L+1(B) (52%)
585.8219	0.0988	H-2(A)->LUMO(A) (72%), H-7(B)->LUMO(B) (15%)
556.5281	0	H-11(A)->LUMO(A) (21%), H-3(A)->LUMO(A) (22%), H-11(B)->LUMO(B) (12%), HOMO(B)->L+1(B) (15%)
539.9266	0.0021	H-4(A)->LUMO(A) (89%)
538.7535	0	H-11(A)->LUMO(A) (13%), H-3(A)->LUMO(A) (62%)

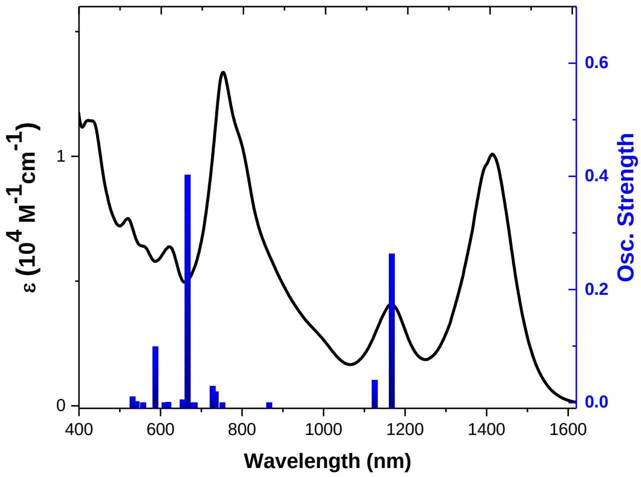

Fig. S39. Calculated stick spectrum (UB3LYP/6-31G*) of the *anti*- **Cor-D1**$^{+\cdot}$ along with the experimental spectrum.

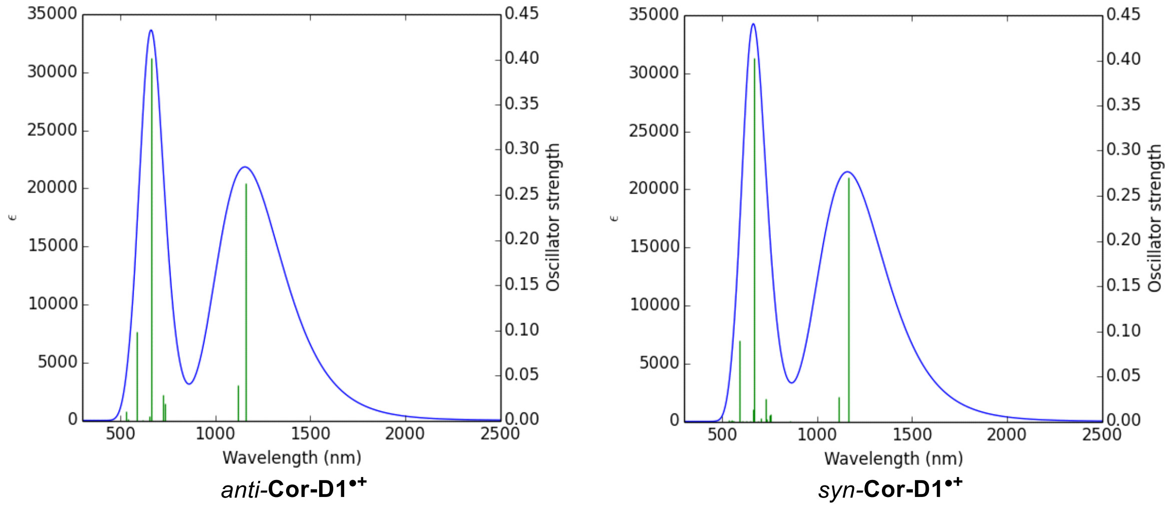

Figure S40. Calculated (UB3LYP/6-31G(d,p)) electronic absorption spectra of the *anti*- and *syn*- isomers of **Cor-D1**$^{\bullet+}$.

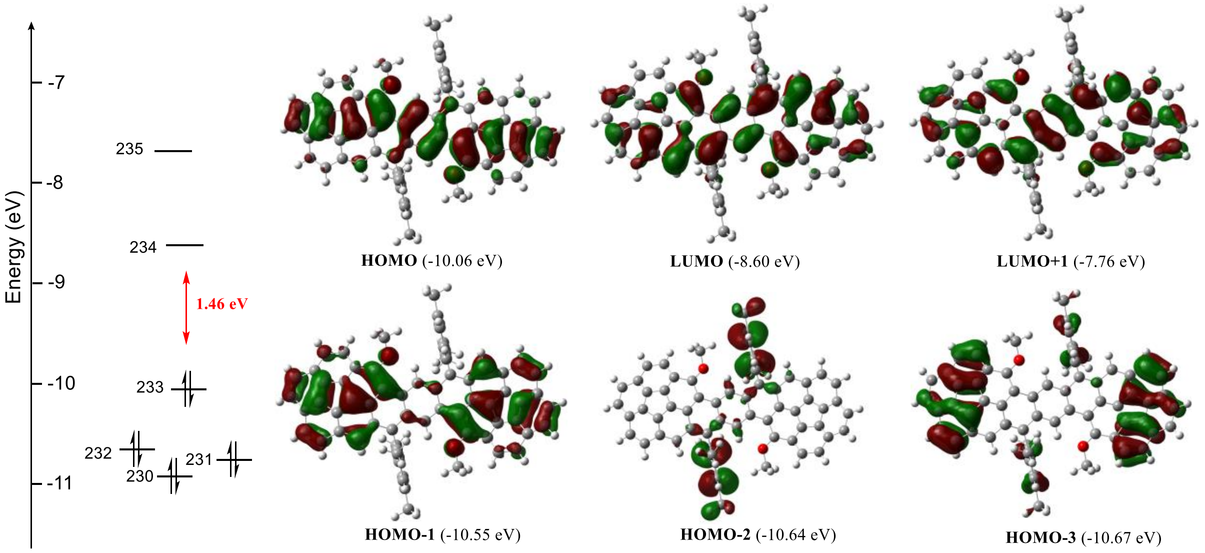

Fig. S41. Frontier molecular orbital profiles and energy diagram of the *anti*- **Cor-D1**$^{2+}$ obtained by B3LYP/6-31G(d,p) level calculation.

Table S7. Selected TD-DFT (B3LYP/6-31G(d)) calculated energies, oscillator strength and compositions of major electronic transitions of the *anti*- **Cor-D1**$^{2+}$.

Wavelength (nm)	Osc. Strength	Major contribs
1163.289	0.026	HOMO->LUMO (98%)
839.2563	0	H-2->LUMO (17%), H-1->LUMO (75%)
829.5419	0	H-2->LUMO (80%), H-1->LUMO (16%)
804.4076	0.0193	H-5->LUMO (75%), H-3->LUMO (14%)
779.3773	0.0002	H-6->LUMO (97%)
779.1814	0	H-7->LUMO (98%)
758.1224	0.0012	H-5->LUMO (14%), H-3->LUMO (82%)
757.4277	0	H-4->LUMO (95%)
718.744	0	H-9->LUMO (46%), HOMO->L+1 (42%)
715.7565	0.0811	H-8->LUMO (85%)
646.5885	0	H-9->LUMO (46%), HOMO->L+1 (52%)
601.4229	0.5886	H-10->LUMO (87%)
535.3339	0.0356	H-1->L+1 (89%)
524.7084	0.0208	H-2->L+1 (96%)
514.9237	0	H-5->L+1 (76%), H-3->L+1 (17%)
503.8335	0	H-6->L+1 (97%)
503.6902	0.0003	H-7->L+1 (98%)
493.6035	0	H-5->L+1 (17%), H-3->L+1 (79%)

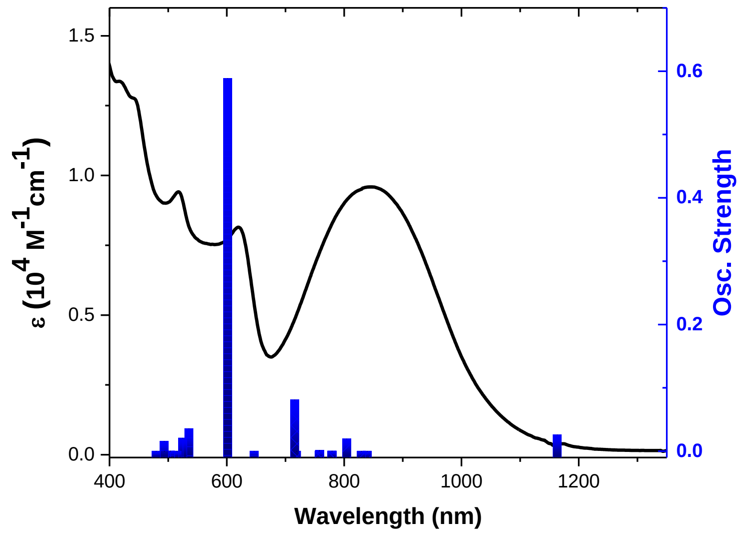

Fig. S42. Calculated stick spectrum (B3LYP/6-31G*) of the *anti*- **Cor-D1^{2+}** along with the experimental spectrum.

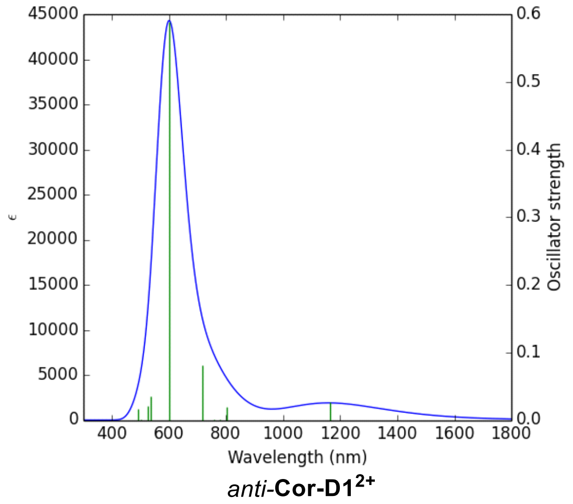

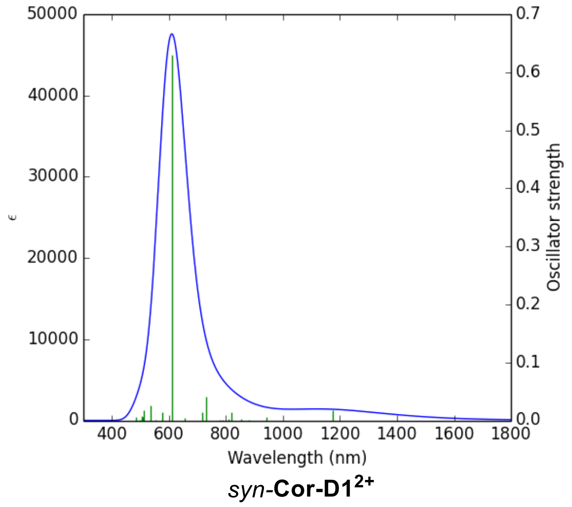

Figure S43. Calculated (B3LYP/6-31G(d,p)) electronic absorption spectra of the *anti*- and *syn*- isomers of **Cor-D1^{2+}**.

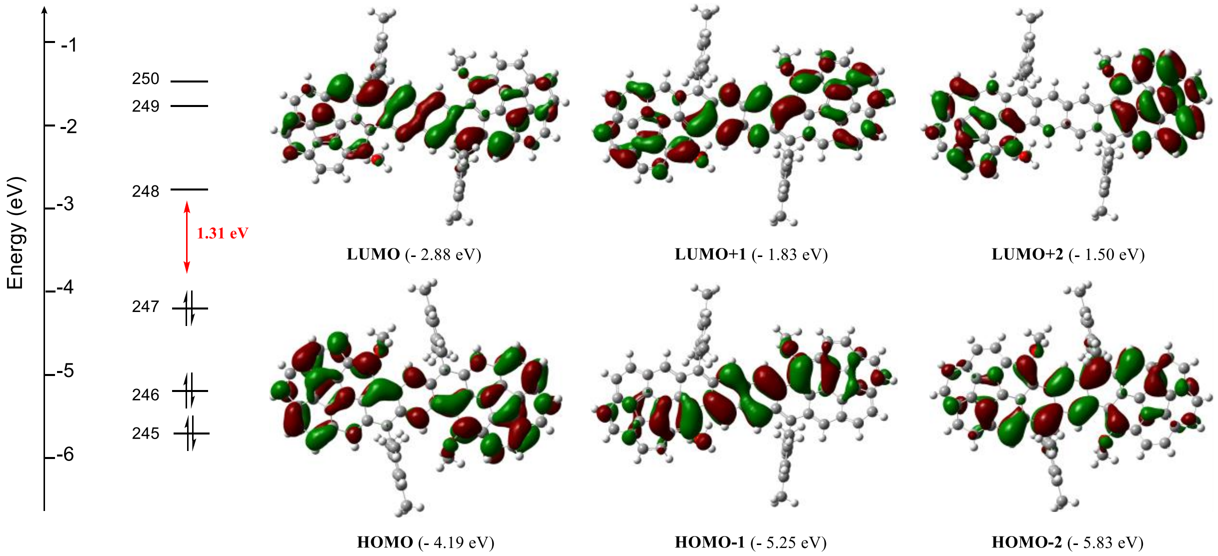

Fig. S44. Frontier molecular orbital profiles and energy diagram of the *anti*- **Cor-D2** obtained by B3LYP/6-31G(d,p) level calculation.

Table S8. Selected TD-DFT (B3LYP/6-31G(d)) calculated energies, oscillator strength and compositions of major electronic transitions of **Cor-D2**.

Wavelength (nm)	Osc. Strength	Major contribs
826.4454	1.2951	HOMO->LUMO (104%)
690.9074	0.0015	H-1->LUMO (71%), HOMO->L+1 (28%)
631.3119	0.0002	H-1->LUMO (26%), HOMO->L+1 (69%)
547.4603	0.0276	HOMO->L+2 (88%)
531.547	0.0013	H-3->LUMO (15%), HOMO->L+3 (78%)
507.4421	0.0303	H-2->LUMO (87%)
470.5607	0.0008	H-3->LUMO (65%)
458.0609	0.0071	H-5->LUMO (19%), H-4->LUMO (73%)
454.651	0.0008	H-6->LUMO (28%), H-5->LUMO (35%), H-4->LUMO (12%)
452.9568	0.0015	H-6->LUMO (54%), H-5->LUMO (33%)
449.6386	0.0083	H-8->LUMO (72%), H-7->LUMO (16%)
444.2733	0.0178	H-9->LUMO (14%), H-8->LUMO (14%), H-7->LUMO (24%), HOMO->L+4 (40%)
441.9138	0.0091	H-9->LUMO (79%), HOMO->L+4 (15%)
436.3306	0.0937	H-7->LUMO (50%), HOMO->L+4 (28%)
423.6719	0.0005	H-10->LUMO (100%)
418.2691	0.0006	H-11->LUMO (100%)
407.612	0.0267	HOMO->L+5 (88%)
404.7907	0.9134	H-1->L+1 (91%)

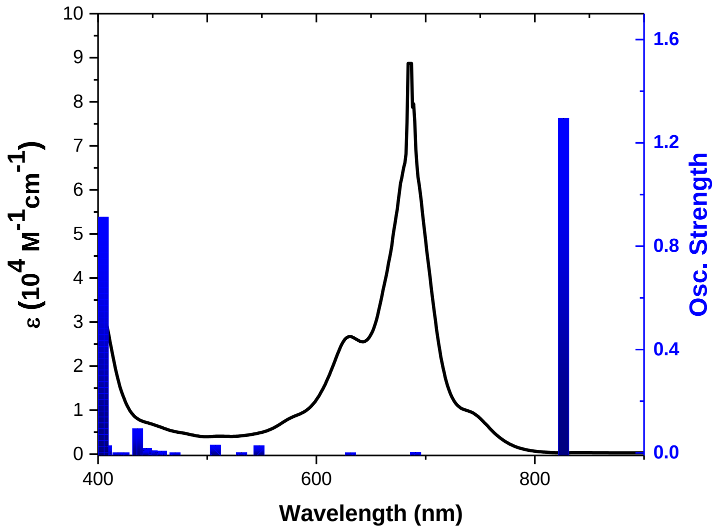

Fig. S45. Calculated stick spectrum (B3LYP/6-31G(d,p)) of the *anti*- **Cor-D2** along with the experimental spectrum. TD DFT calculation on open-shell singlet diradicaloids is a challenging problem and there is a significant discrepancy on the wavelength.

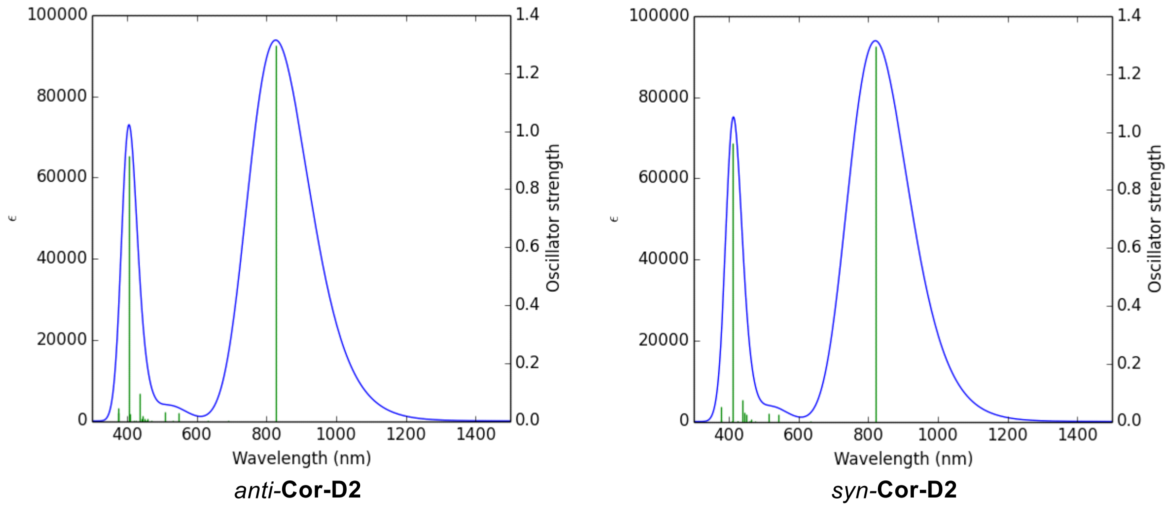

Figure S46. Calculated (B3LYP/6-31G(d,p)) electronic absorption spectra of the *anti*- and *syn*- isomers of **Cor-D2**.

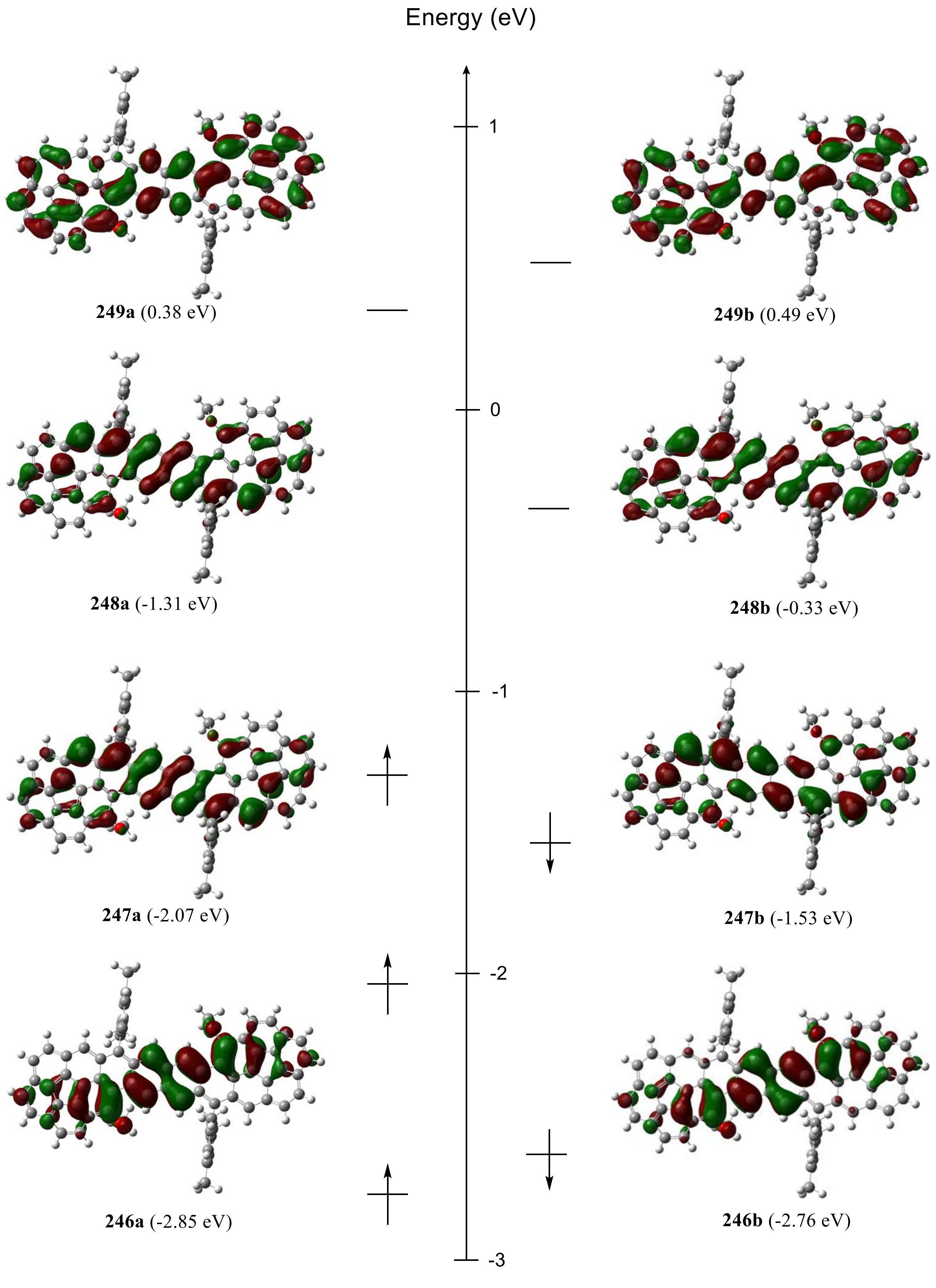

Fig. S47. Frontier molecular orbital profiles and energy diagram of the *anti*- **Cor-D2**$^{-\cdot}$ obtained by UB3LYP/6-31G(d,p) level calculation.

Table S9. Selected TD-DFT (B3LYP/6-31G(d)) calculated energies, oscillator strength and compositions of major electronic transitions of the *anti*- **Cor-D2**$^{-\cdot}$.

Wavelength (nm)	Osc. Strength	Major contribs
1431.678	0.4115	HOMO(B)->LUMO(B) (94%)
1054.998	0.1398	HOMO(A)->LUMO(A) (94%)
917.24	0.0018	H-1(A)->LUMO(A) (11%), HOMO(A)->L+1(A) (26%), HOMO(B)->L+1(B) (55%)
849.5501	0.0378	HOMO(A)->L+1(A) (59%), HOMO(B)->L+1(B) (19%)
825.0155	0.2472	HOMO(A)->L+2(A) (72%)
715.7152	0.0013	H-1(A)->LUMO(A) (14%), H-1(B)->LUMO(B) (47%), HOMO(B)->L+1(B) (20%)
694.5456	0.0692	HOMO(A)->L+2(A) (10%), HOMO(B)->L+2(B) (74%)
681.2271	0.0091	HOMO(A)->L+3(A) (12%), H-1(B)->LUMO(B) (18%), HOMO(B)->L+3(B) (53%)
614.9662	0.0004	H-1(A)->LUMO(A) (44%), HOMO(A)->L+3(A) (39%)
605.3283	0.0003	H-1(A)->LUMO(A) (26%), HOMO(A)->L+3(A) (30%), HOMO(B)->L+3(B) (21%)
598.5774	0.0189	H-2(A)->LUMO(A) (30%), H-1(B)->L+1(B) (29%)
573.1213	0.0011	HOMO(A)->L+4(A) (36%), HOMO(B)->L+4(B) (35%)
541.2938	0.0292	H-1(A)->L+1(A) (49%), HOMO(B)->L+4(B) (21%)
534.3418	0.0217	H-1(A)->L+2(A) (47%), H-2(B)->LUMO(B) (20%), HOMO(B)->L+4(B) (10%)
516.4036	0.1014	H-1(A)->L+1(A) (24%), HOMO(A)->L+4(A) (45%), HOMO(B)->L+4(B) (17%)
492.2904	0.0002	H-1(A)->L+2(A) (28%), H-2(B)->LUMO(B) (53%)
487.0304	0.0003	HOMO(B)->L+5(B) (67%)
482.5569	0.0066	H-3(B)->LUMO(B) (77%)

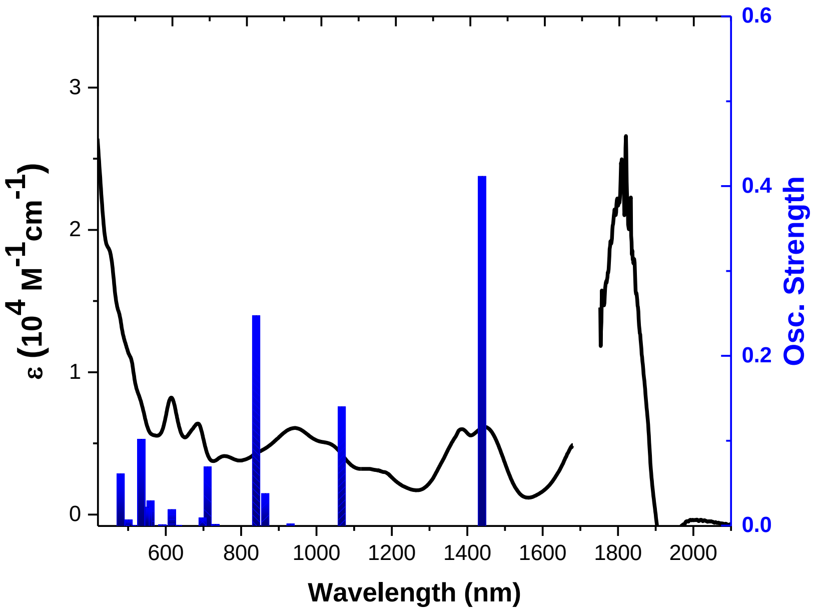

Fig. S48. Calculated stick spectrum (UB3LYP/6-31G*) of the *anti*- **Cor-D2**$^{-\cdot}$ along with the experimental spectrum.

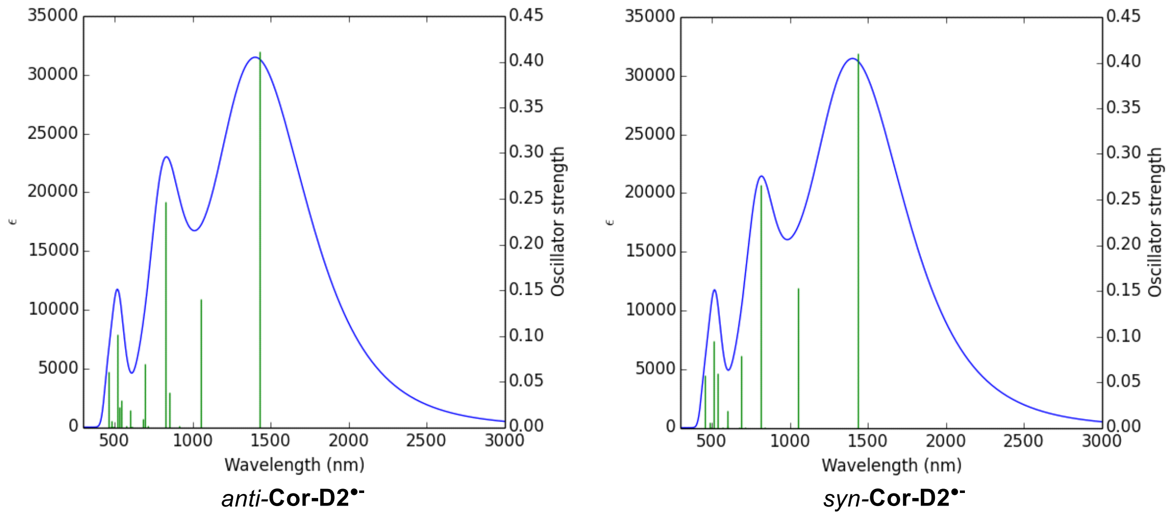

Figure S49. Calculated (UB3LYP/6-31G(d,p)) electronic absorption spectra of the *anti*- and *syn*- isomers of **Cor-D2**$^{\bullet-}$.

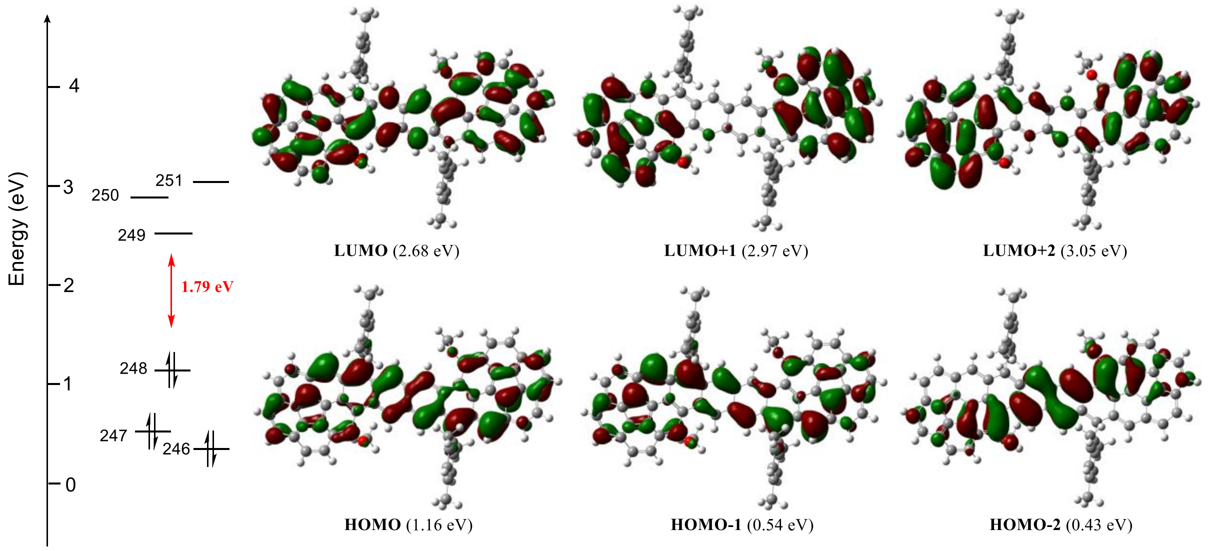

Fig. S50. Frontier molecular orbital profiles and energy diagram of the *anti*- **Cor-D2^{2-}** obtained by B3LYP/6-31G(d,p) level calculation.

Table S10. Selected TD-DFT (B3LYP/6-31G(d)) calculated energies, oscillator strength and compositions of major electronic transitions of the *anti*- **Cor-D2^{2-}**.

Wavelength (nm)	Osc. Strength	Major contribs
1053.205	0.0667	HOMO->LUMO (93%)
902.2876	0.0029	HOMO->L+1 (92%)
780.0638	0.5211	HOMO->L+2 (87%)
703.3717	0.0001	H-1->LUMO (91%)
629.1973	0.0003	HOMO->L+3 (92%)
606.068	0.0272	H-1->L+1 (88%)
570.7731	0.0009	H-1->L+2 (92%)
541.5775	0.1531	HOMO->L+4 (87%)
518.5418	0.0998	HOMO->L+5 (90%)
506.882	0.001	HOMO->L+6 (90%)
489.0283	0.096	H-1->L+3 (94%)
481.4887	0.0009	HOMO->L+7 (92%)
474.5047	0.0017	HOMO->L+8 (91%)
457.7734	0.0223	H-2->LUMO (34%), HOMO->L+9 (56%)
442.2291	1.0869	H-2->LUMO (58%), HOMO->L+9 (34%)
438.429	0.0095	H-1->L+4 (62%), HOMO->L+10 (27%)
422.56	0.0001	H-2->L+1 (18%), H-1->L+4 (25%), HOMO->L+10 (45%)
413.4432	0.0033	H-2->L+1 (73%), HOMO->L+10 (13%)

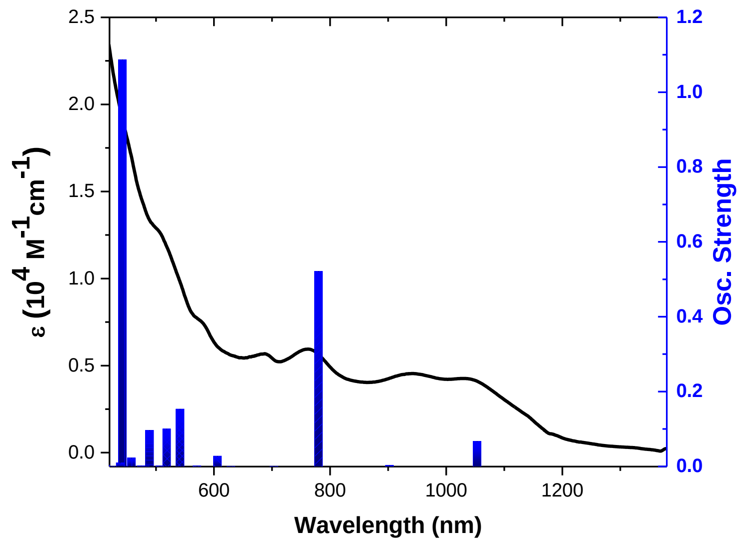

Fig. S51. Calculated stick spectrum (B3LYP/6-31G*) of the *anti*- **Cor-D2^{2-}** along with the experimental spectrum.

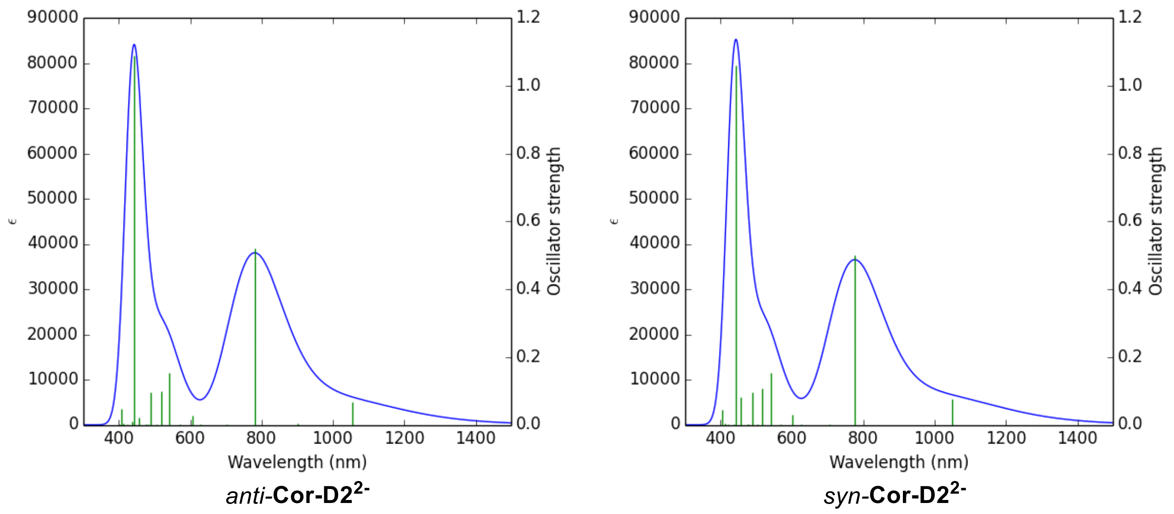

Figure S52. Calculated (B3LYP/6-31G(d,p)) electronic absorption spectra of the *anti*- and *syn*- isomers of **Cor-D2^{2-}**.

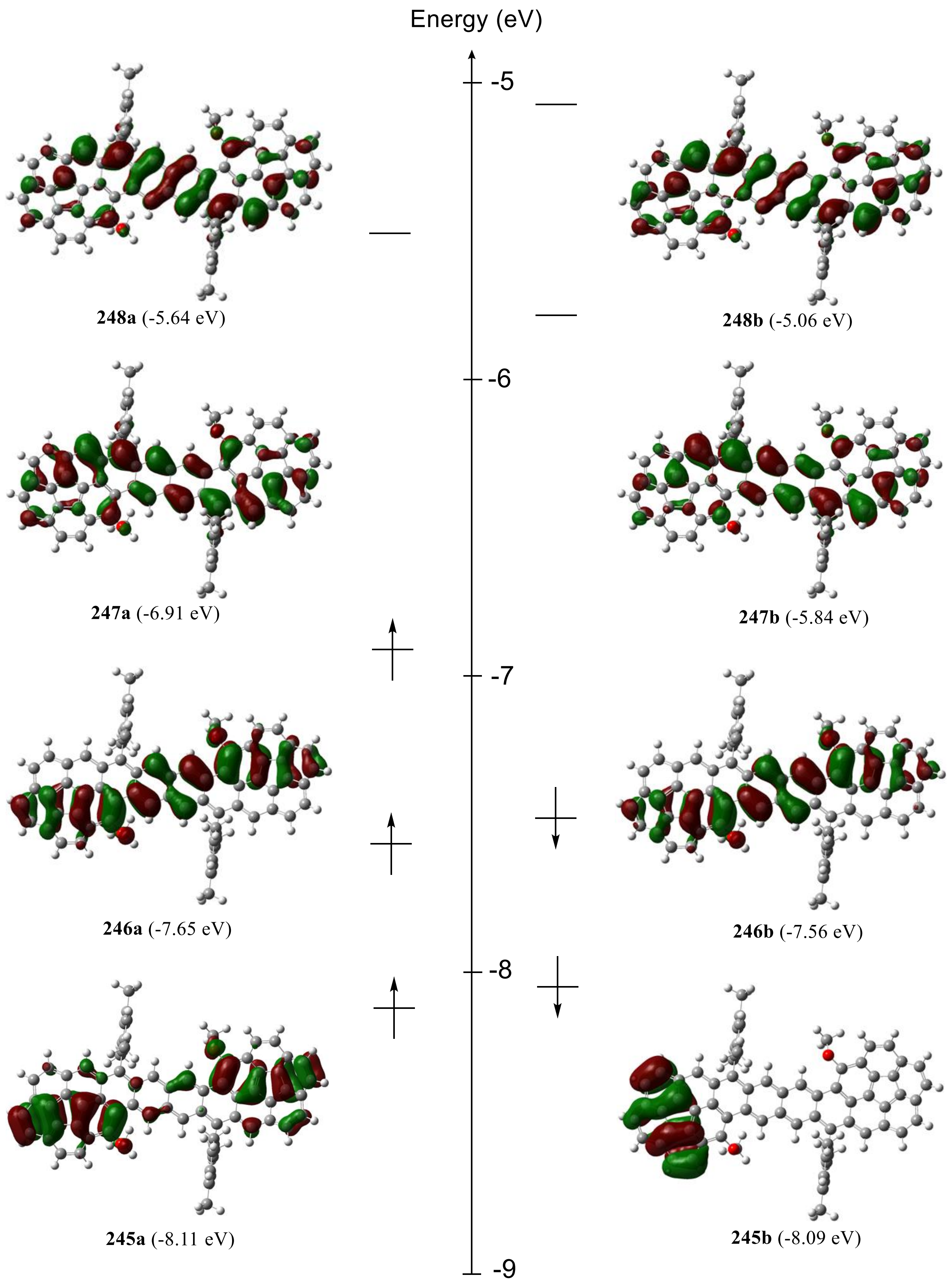

Fig. S53. Frontier molecular orbital profiles and energy diagram of the *anti*- **Cor-D2**$^{+\cdot}$ obtained by UB3LYP/6-31G(d,p) level calculation.

Table S11. Selected TD-DFT (UB3LYP/6-31G(d)) calculated energies, oscillator strength and compositions of major electronic transitions of the *anti*- **Cor-D2**$^{+\cdot}$.

Wavelength (nm)	Osc. Strength	Major contribs
1386.063	0.3764	HOMO(A)->LUMO(A) (91%)
1071.686	0.1048	HOMO(B)->LUMO(B) (95%)
937.6339	0.0005	H-1(A)->LUMO(A) (81%)
767.7462	0.0006	H-1(B)->LUMO(B) (74%)
737.6009	0.2366	H-2(A)->LUMO(A) (19%), H-4(B)->LUMO(B) (24%), H-3(B)->LUMO(B) (13%)
701.6204	0.0055	H-3(B)->LUMO(B) (10%), H-2(B)->LUMO(B) (50%)
691.6011	0.0226	H-4(B)->LUMO(B) (11%), H-3(B)->LUMO(B) (19%), H-2(B)->LUMO(B) (20%), HOMO(B)->L+1(B) (11%)
676.3957	0.0146	H-3(B)->LUMO(B) (35%), HOMO(B)->L+1(B) (21%)
664.505	0.1028	H-5(B)->LUMO(B) (62%), H-4(B)->LUMO(B) (18%)
654.7493	0.0042	H-6(B)->LUMO(B) (66%)
646.8921	0.0088	H-7(B)->LUMO(B) (81%)
636.3994	0.1221	H-2(A)->LUMO(A) (49%), H-8(B)->LUMO(B) (11%), H-4(B)->LUMO(B) (17%)
633.7644	0.0248	H-8(B)->LUMO(B) (81%)
606.6018	0.0024	H-5(A)->LUMO(A) (10%), H-4(A)->LUMO(A) (34%), HOMO(A)->L+1(A) (22%)
605.7127	0.0002	H-9(B)->LUMO(B) (94%)
594.7584	0	H-10(B)->LUMO(B) (96%)
584.7995	0.0028	HOMO(A)->L+1(A) (35%), HOMO(B)->L+1(B) (34%)
581.0176	0.0006	H-3(A)->LUMO(A) (78%)

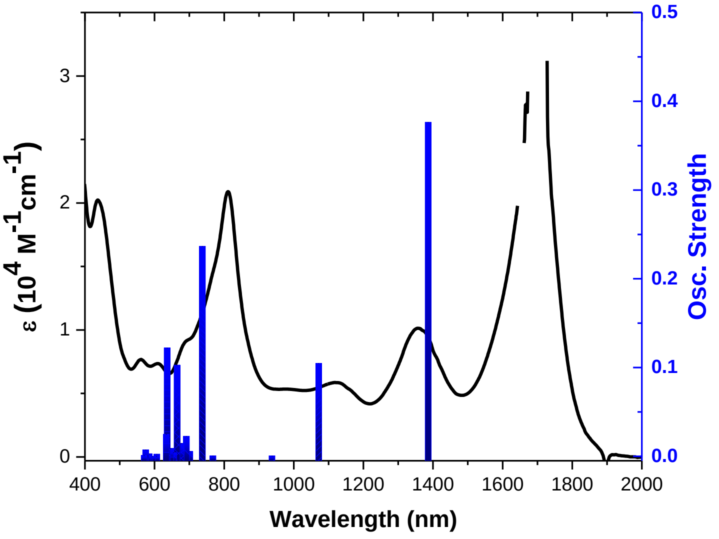

Fig. S54. Calculated stick spectrum (B3LYP/6-31G*) of the *anti*- **Cor-D2**$^{+\cdot}$ along with the experimental spectrum.

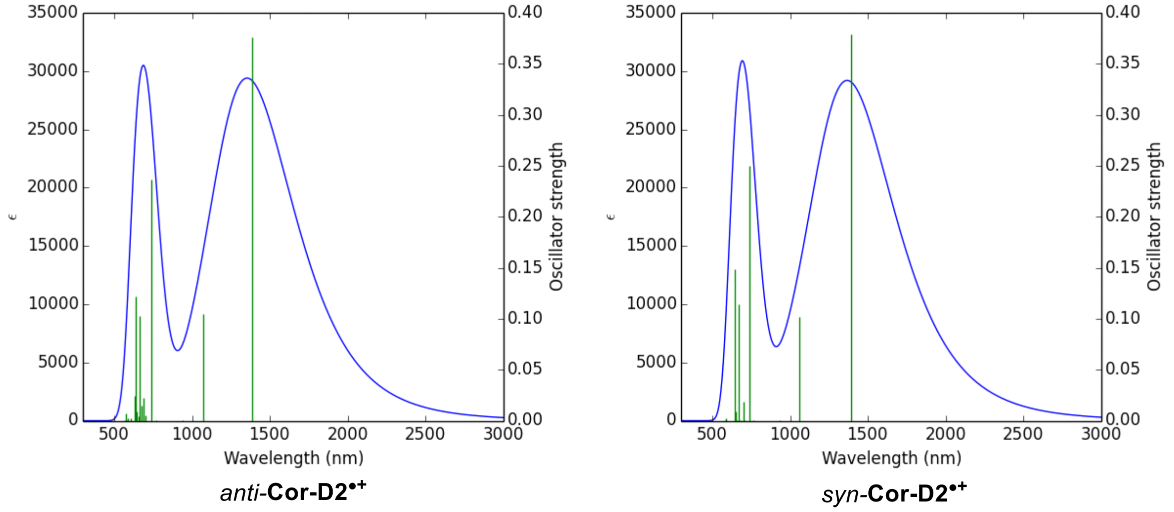

Figure S55. Calculated (UB3LYP/6-31G(d,p)) electronic absorption spectra of the *anti*- and *syn*- isomers of **Cor-D2**$^{+•}$.

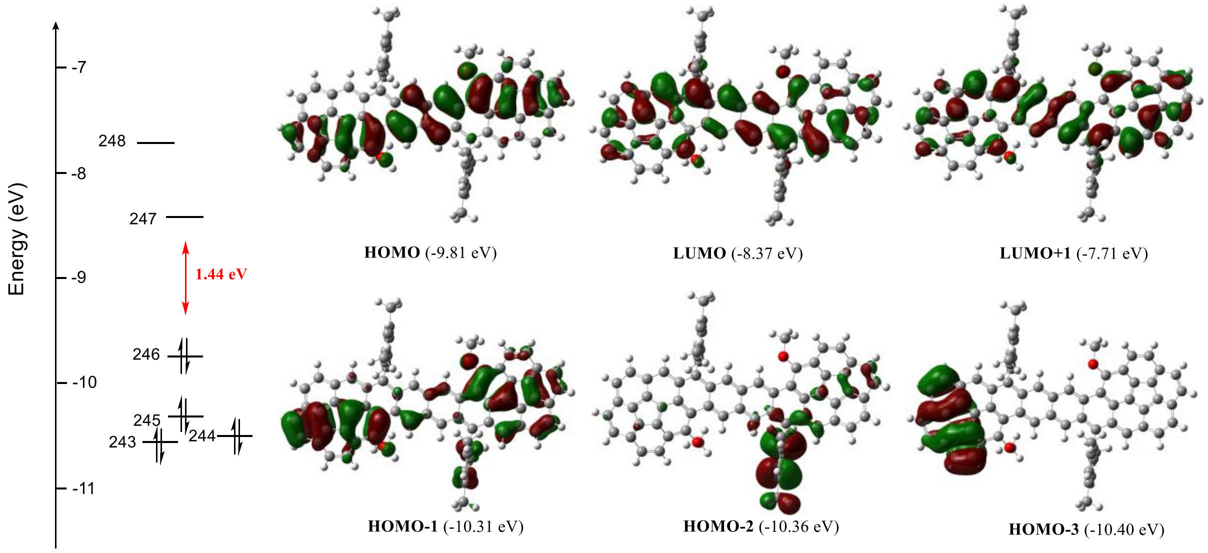

Fig. S56. Frontier molecular orbital profiles and energy diagram of the *anti*- **Cor-D2**$^{2+}$ obtained by B3LYP/6-31G(d,p) level calculation.

Table S12. Selected TD-DFT (B3LYP/6-31G(d)) calculated energies, oscillator strength and compositions of major electronic transitions of the *anti*- **Cor-D2**$^{2+}$.

Wavelength (nm)	Osc. Strength	Major contribs
1187.352	0.0338	HOMO->LUMO (97%)
862.6128	0.0045	H-1->LUMO (74%), HOMO->L+1 (20%)
832.7736	0.0098	H-2->LUMO (90%)
797.6796	0.0001	H-4->LUMO (94%)
786.2473	0.0255	H-7->LUMO (20%), H-6->LUMO (73%)
768.0316	0.0022	H-3->LUMO (95%)

759.2366	0.0004	H-8->LUMO (89%)
740.0665	0.0064	H-1->LUMO (20%), HOMO->L+1 (61%)
731.9401	0.0019	H-5->LUMO (87%)
688.7581	0.159	H-10->LUMO (34%), H-9->LUMO (18%), H-7->LUMO (31%)
667.4742	0.0126	H-10->LUMO (30%), H-9->LUMO (54%), HOMO->L+1 (10%)
666.3621	0.589	H-10->LUMO (29%), H-9->LUMO (13%), H-7->LUMO (34%), H-6->LUMO (11%)
572.1692	0.0492	H-2->L+1 (18%), H-1->L+1 (72%)
567.0402	0.0021	H-2->L+1 (75%), H-1->L+1 (18%)
551.7236	0.0003	H-4->L+1 (94%)
529.0069	0.0094	H-6->L+1 (81%)
526.6698	0.0053	H-3->L+1 (92%)
521.7056	0.0057	H-5->L+1 (93%)

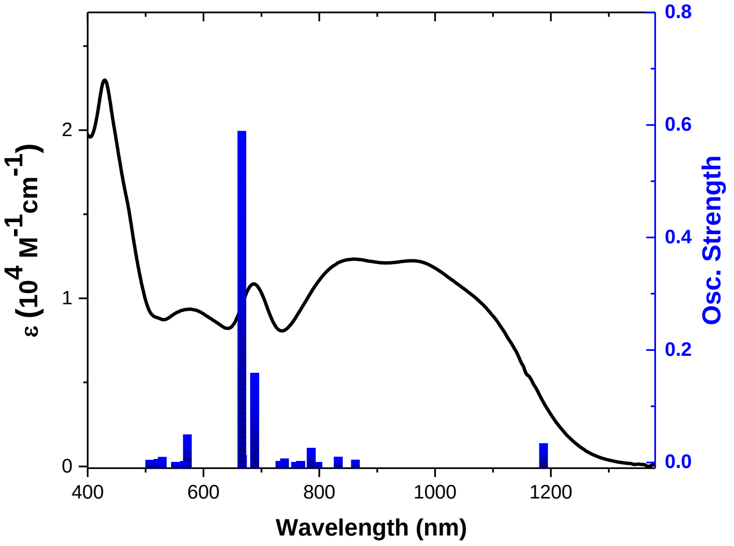

Fig. S57. Calculated stick spectrum (B3LYP/6-31G*) of the *anti*- **Cor-D2**$^{2+}$ along with the experimental spectrum.

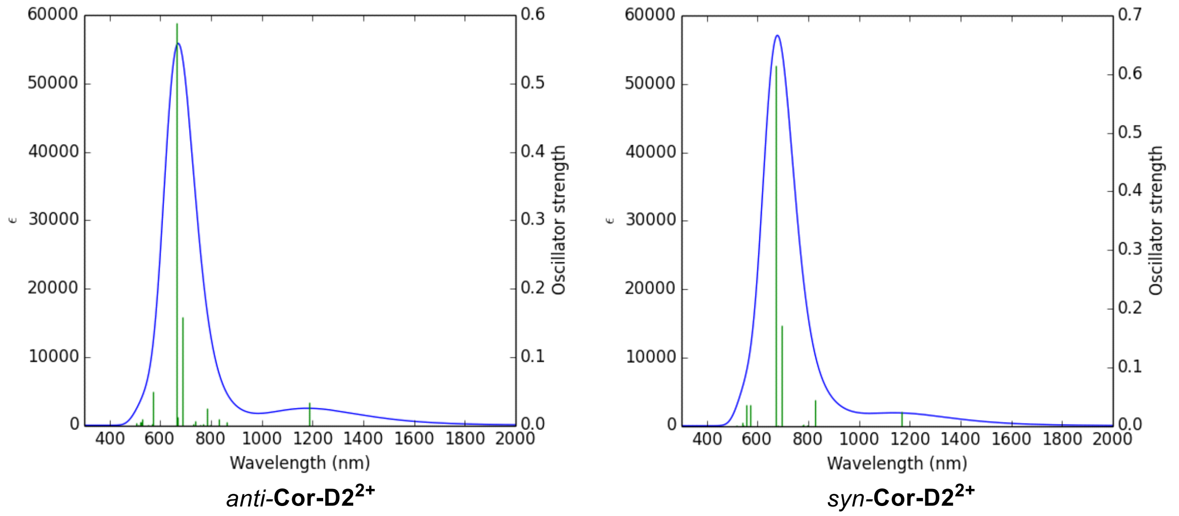

Figure S58. Calculated (B3LYP/6-31G(d,p)) electronic absorption spectra of the *anti*- and *syn*- isomers of **Cor-D2^{2+}**.

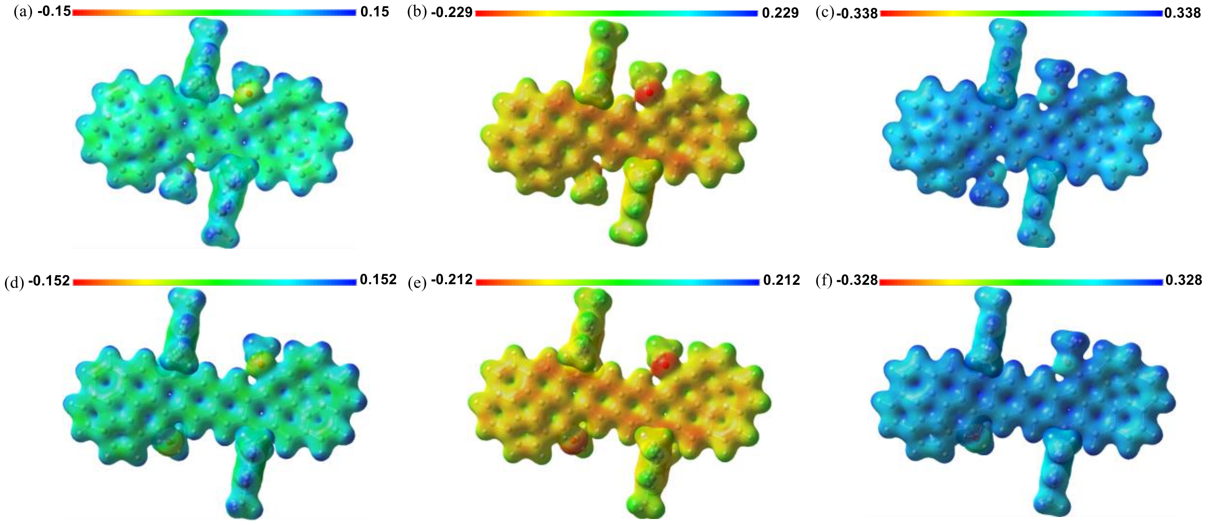

Fig. S59. Electrostatic potential maps of (a) **Cor-D1**, (b) **Cor-D1^{2-}**, (c) **Cor-D1^{2+}**, (d) **Cor-D2**, (e) **Cor-D2^{2-}** and (f) **Cor-D2^{2+}** (all in the *anti*- form).

Table S13. Relative energies (kcal/mol) of the *anti*- and *syn*- isomer of **Cor-D1** and **Cor-D2** at different redox states.

Energies	**Cor-D1**	**Cor-D1^{2-}**	**Cor-D1^{2+}**	**Cor-D1$^{•-}$**	**Cor-D1$^{•+}$**
Anti-	0	0	0	0	0
Syn-	+0.538	-1.142	+4.686	-0.432	+1.539
Energies	**Cor-D2**	**Cor-D2^{2-}**	**Cor-D2^{2+}**	**Cor-D2$^{•-}$**	**Cor-D2$^{•+}$**
Anti-	0	0	0	0	0
Syn-	-0.094	-1.380	+3.145	-0.826	+0.869

6. X-ray crystallographic data

Table S14. Crystallographic data for **Cor-D1**.

**Chemical formula**	$C_{40}H_{27}ClO$	
**Formula weight**	559.06 g/mol	
**Temperature**	100(2) K	
**Wavelength**	1.54178 Å	
**Crystal size**	0.122 x 0.145 x 0.255 mm	
**Crystal system**	triclinic	
**Space group**	P -1	
**Unit cell dimensions**	a = 10.7657(7) Å	α = 65.614(3)°
	b = 11.9402(7) Å	β = 84.355(3)°
	c = 12.6973(7) Å	γ = 71.603(4)°
**Volume**	1409.62(15) $Å^3$	
**Z**	2	
**Density (calculated)**	1.317 g/cm^3	
**Absorption coefficient**	1.441 mm^{-1}	
**F(000)**	584	

Table S15. Data collection and structure refinement for **Cor-D1**.

**Theta range for data collection**	4.27 to 62.36°	
**Index ranges**	-12<=h<=12, -13<=k<=13, -14<=l<=13	
**Reflections collected**	11807	
**Independent reflections**	3995 [R(int) = 0.0383]	
**Coverage of independent reflections**	89.1%	
**Absorption correction**	multi-scan	
**Max. and min. transmission**	0.8440 and 0.7100	
**Refinement method**	Full-matrix least-squares on F^2	
**Refinement program**	SHELXL-2014/7 (Sheldrick, 2014)	
**Function minimized**	$\Sigma w(F_o^2 - F_c^2)^2$	
**Data / restraints / parameters**	3995 / 0 / 383	
**Goodness-of-fit on F^2**	1.435	
**Final R indices**	2858 data; I>2σ(I)	R1 = 0.1049, wR2 = 0.3586
	all data	R1 = 0.1307, wR2 = 0.3667
**Weighting scheme**	$w=1/[\sigma^2(F_o^2)+12.8190P]$ where $P=(F_o^2+2F_c^2)/3$	
**Largest diff. peak and hole**	0.526 and -0.720 $eÅ^{-3}$	

**R.M.S. deviation from mean**	0.055 eÅ^{-3}

Table S16. Crystallographic data for **Cor-D2**.

**Chemical formula**	0.5($C_{72}H_{46}O_2$), (C_6H_5Cl)	
**Formula weight**	584.09 g/mol	
**Temperature**	93 K	
**Wavelength**	1.54184 Å	
**Crystal size**	0.01 x 0.03 x 0.20 mm	
**Crystal system**	triclinic	
**Space group**	P -1	
**Unit cell dimensions**	a = 7.3515(7) Å	α = 74.348(9)°
	b = 11.2839(11) Å	β = 87.386(8)°
	c = 19.4024(19) Å	γ = 73.241(8)°
**Volume**	1483.1(3) Å^3	
**Z**	2	
**Density (calculated)**	1.308 g/cm^3	
**Absorption coefficient**	1.394 mm^{-1}	
**F(000)**	610.0	

Table S17. Data collection and structure refinement for **Cor-D2**.

**Theta range for data collection**	25.5 to 65.7°	
**Index ranges**	-8<=h<=8, -12<=k<=12, -22<=l<=22	
**Reflections collected**	17557	
**Independent reflections**	4657, [R(int) = 0.121]	
**Coverage of independent reflections**	98.2 %	
**Absorption correction**	multi-scan	
**Max. and min. transmission**	0.986 and 0.951	
**Refinement method**	Full-matrix least-squares on F^2	
**Refinement program**	SHELXL-2014/7 (Sheldrick, 2014)	
**Function minimized**	$\Sigma w(F_o^2 - F_c^2)^2$	
**Data / parameters**	4657/ 427	
**Goodness-of-fit on F^2**	1.002	
**Final R indices**	2858 data; $I>2\sigma(I)$	R1 = 0.0854, wR2 = 0.2120
	all data	R1 = 0.1661, wR2 = 0.2730
**Largest diff. peak and hole**	0.55 and -0.38 eÅ^{-3}	

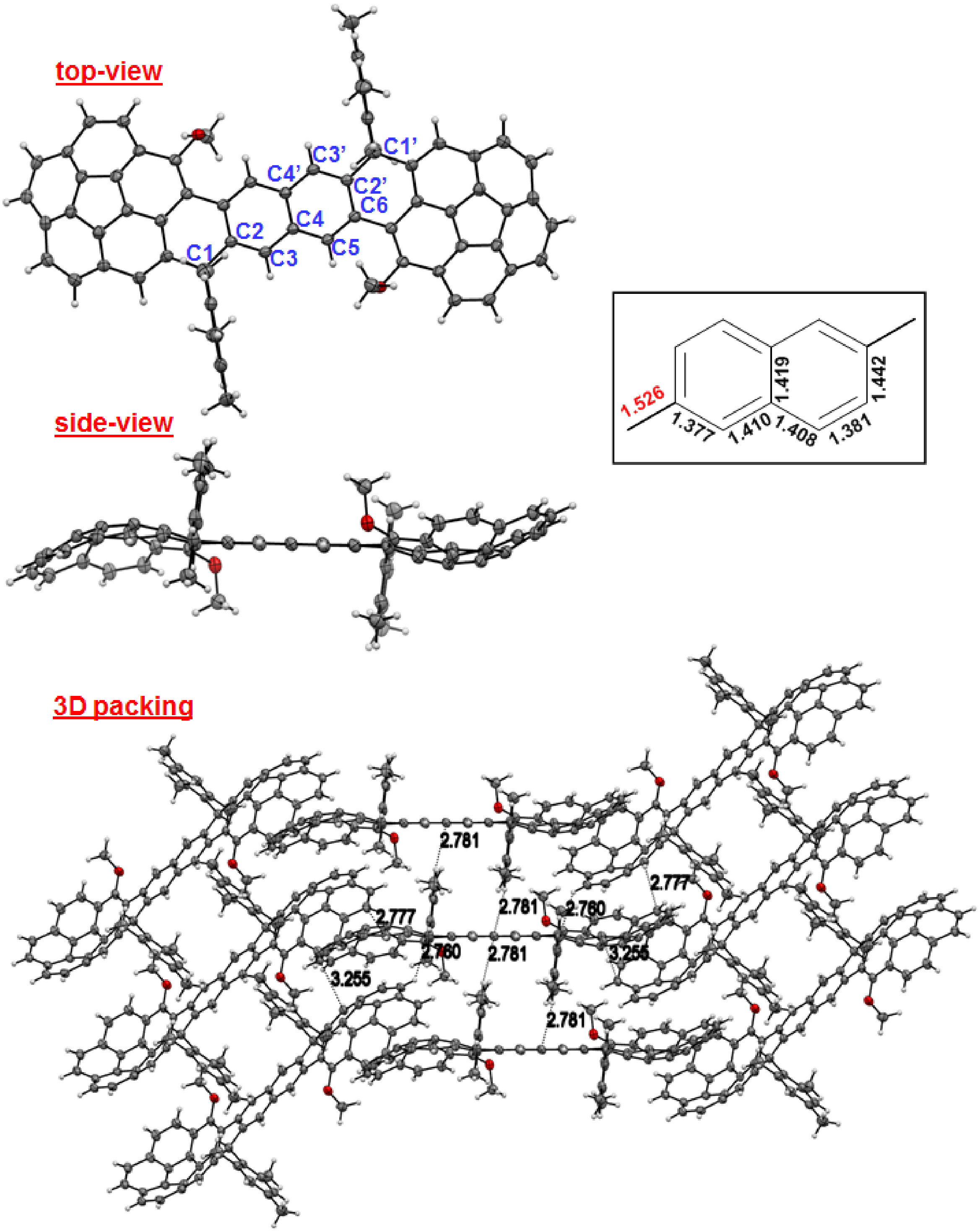

Fig. S60. X-ray crystallographic structure of **Cor-D2-2H** (top-view, side-view and 3D packing), together with selected bond lengths (in Å) for the central 2,6-naphthyl dimethylene unit. There are multiple [CH•••π] interactions between the methyl groups and the backbone, and also π-π and [CH•••π] interactions between the corannulene bowls.

Table S18. Crystallographic data for **Cor-D2-2H**.

**Chemical formula**	$C_{72}H_{48}O_2$

**Formula weight**	945.10 g/mol	
**Temperature**	93 K	
**Wavelength**	1.54184 Å	
**Crystal size**	0.01 x 0.05 x 0.05 mm	
**Crystal system**	Monoclinic	
**Space group**	P 21/c	
**Unit cell dimensions**	a = 10.7377(4) Å	α = 90°
	b = 7.2096(3) Å	β = 97.730(3)°
	c = 30.6438(10) Å	γ = 90°
**Volume**	2350.72(15) $Å^3$	
**Z**	2	
**Density (calculated)**	1.335 g/cm^3	
**Absorption coefficient**	0.605 mm^{-1}	
**F(000)**	992.0	

Table S19. Data collection and structure refinement for **Cor-D2-2H**.

**Theta range for data collection**	2.9 to 67.0°	
**Index ranges**	-12<=h<=12, -8<=k<=8, -31<=l<=36	
**Reflections collected**	15717	
**Independent reflections**	4182 [R(int) = 0.052]	
**Coverage of independent reflections**	99.6 %	
**Absorption correction**	multi-scan	
**Max. and min. transmission**	0.994 and 0.970	
**Refinement method**	Full-matrix least-squares on F^2	
**Refinement program**	SHELXL-2014/7 (Sheldrick, 2014)	
**Function minimized**	$\Sigma w(F_o^2 - F_c^2)^2$	
**Data / parameters**	4182/338	
**Goodness-of-fit on F^2**	1.01	
**Final R indices**	data; I>2σ(I)	R1 = 0.0475, wR2 = 0.1140
	all data	R1 = 0.0739, wR2 = 0.1294
**Weighting scheme**	$w=1/[\sigma^2(F_o^2)+12.8190P]$ where $P=(F_o^2+2F_c^2)/3$	
**Largest diff. peak and hole**	0.24 and -0.23 $eÅ^{-3}$	

7. Appendix: NMR spectra and HR mass spectra

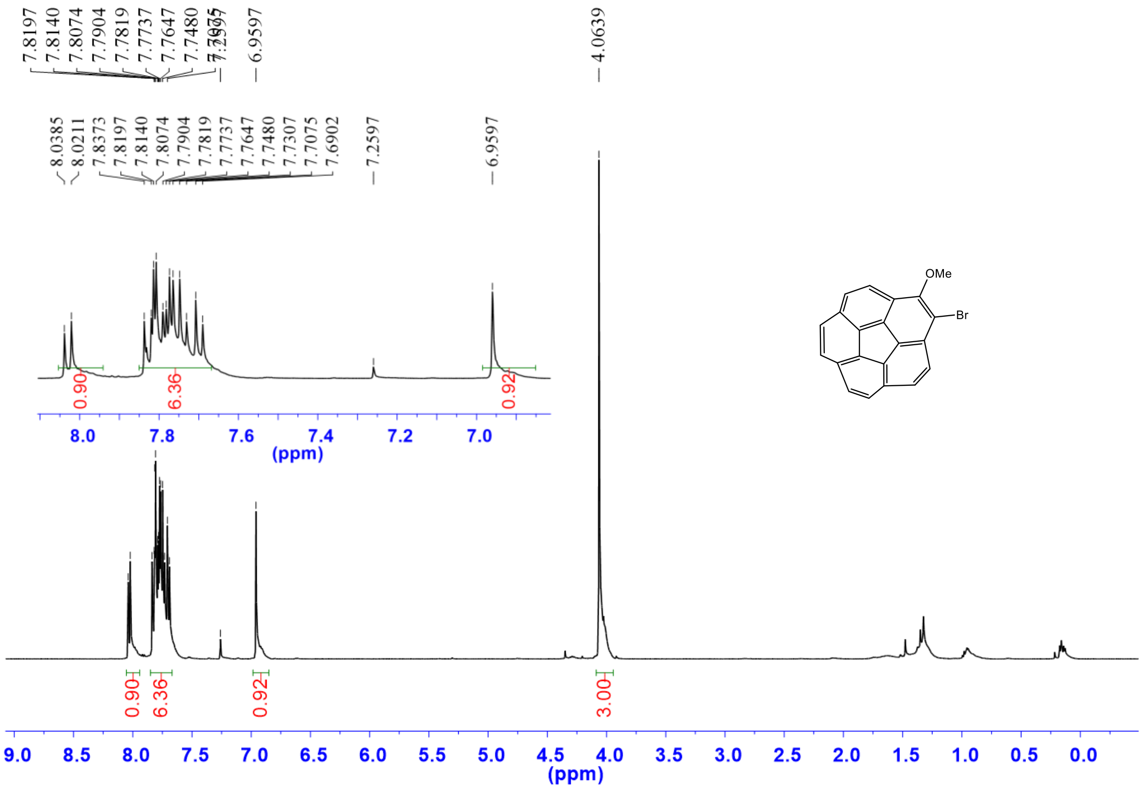

Fig. S61. ^{1}H NMR spectrum of compound **2** (300 MHz, $CDCl_3$, rt)

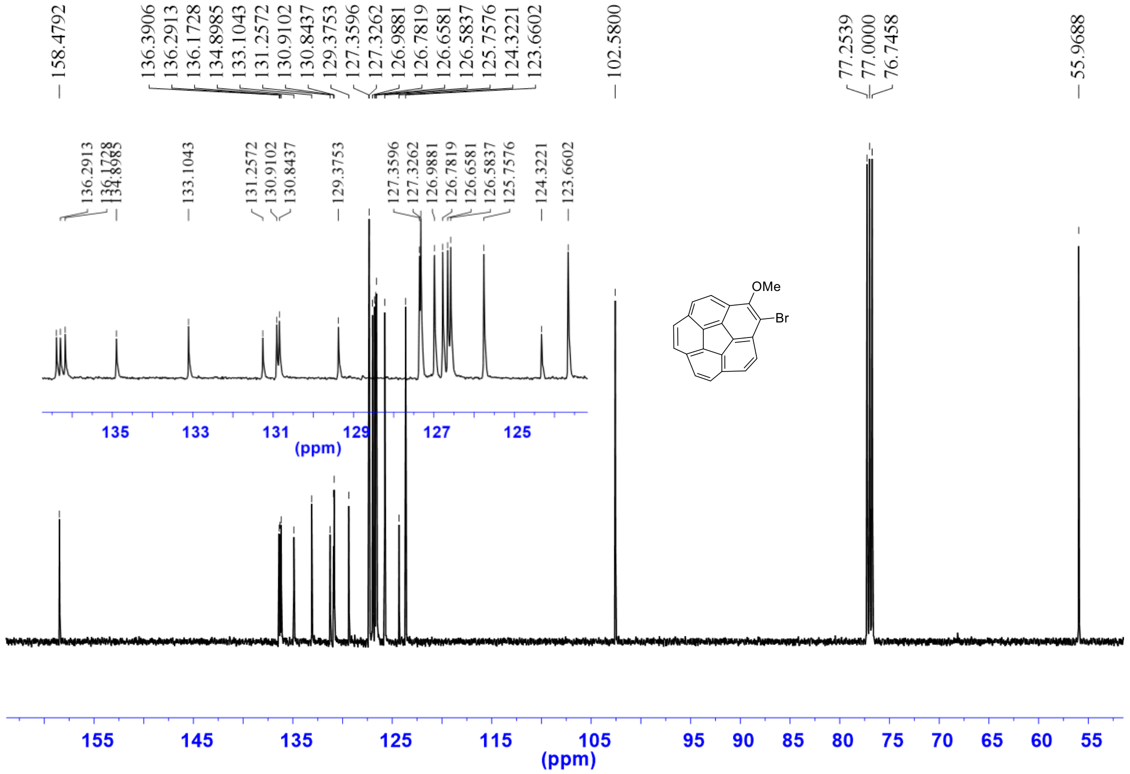

Fig. S62. ^{13}C NMR spectrum of compound **2** (125 MHz, $CDCl_3$, rt)

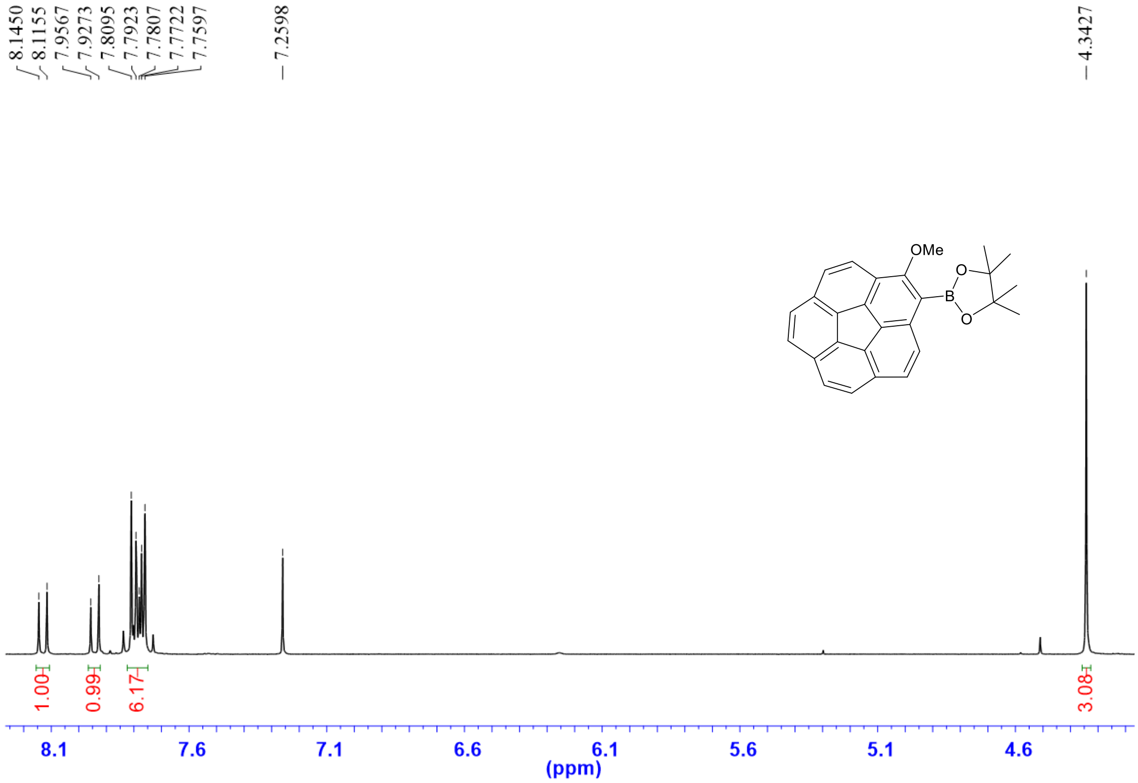

Fig. S63. ^{1}H NMR spectrum of compound **3** (300 MHz, $CDCl_3$, rt).

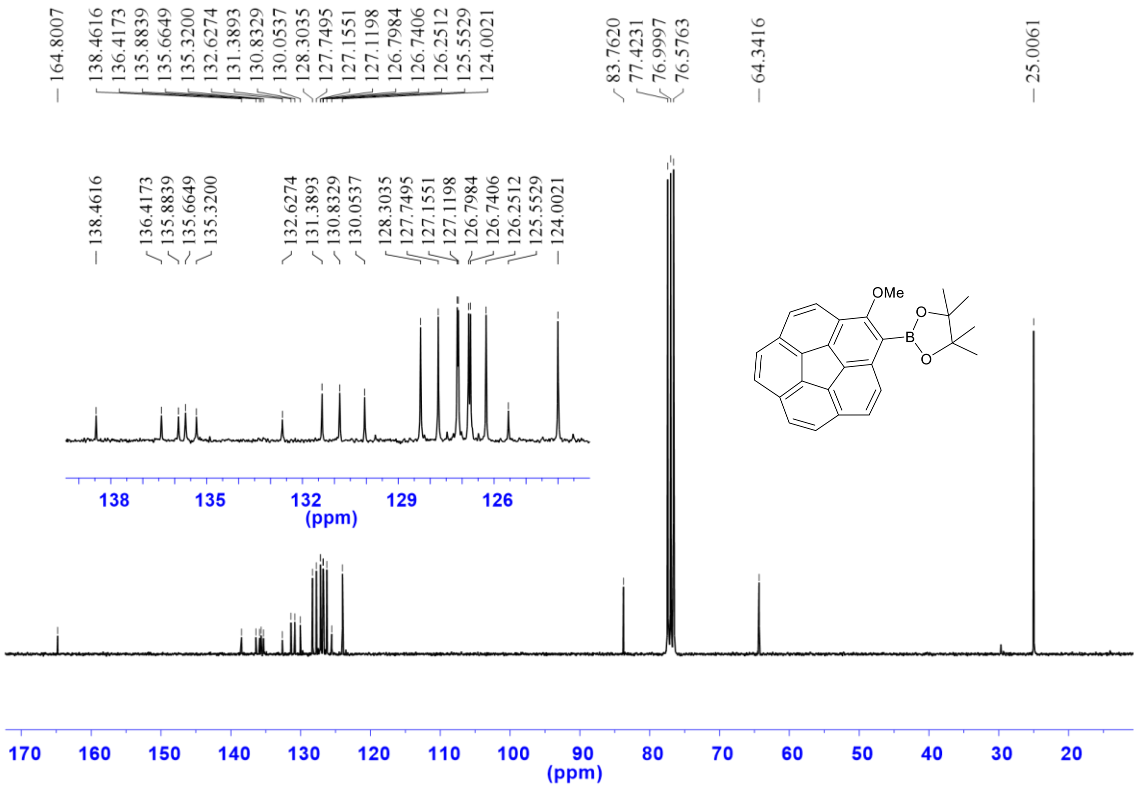

Fig. S64. ^{13}C NMR spectrum of compound **3** (125 MHz, $CDCl_3$, rt)

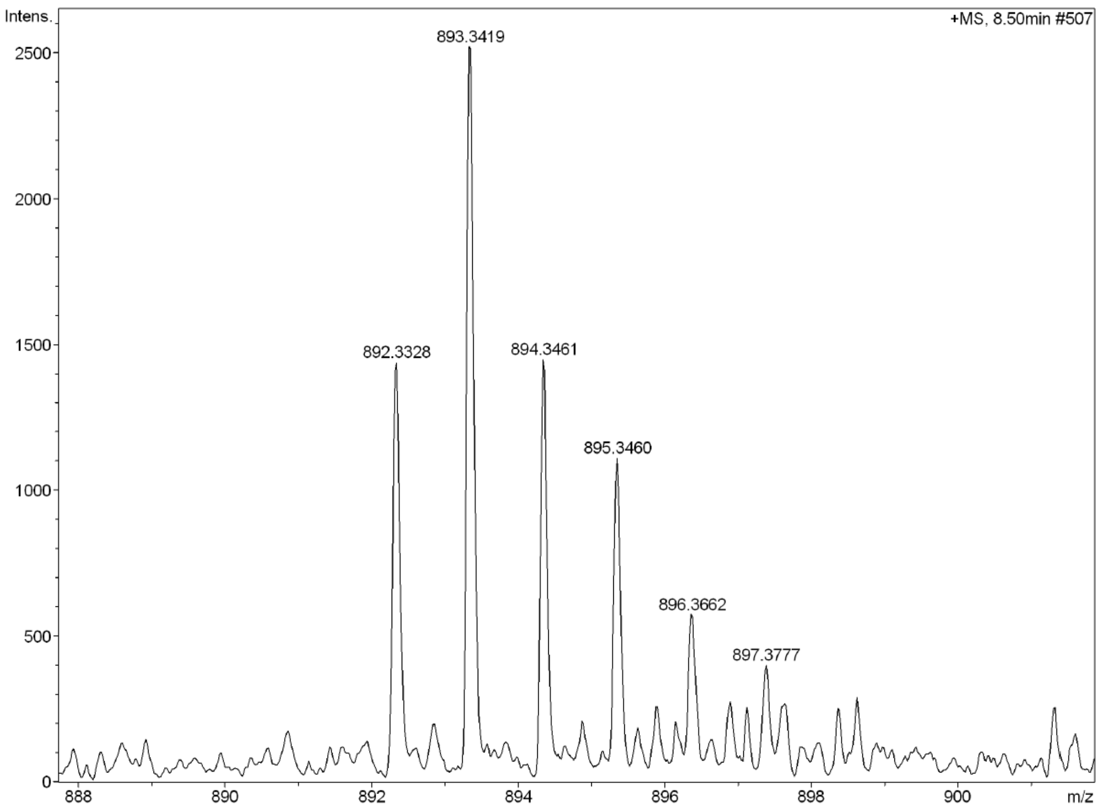

Fig. S65. HR mass spectrum (ESI) of **Cor-D1**.

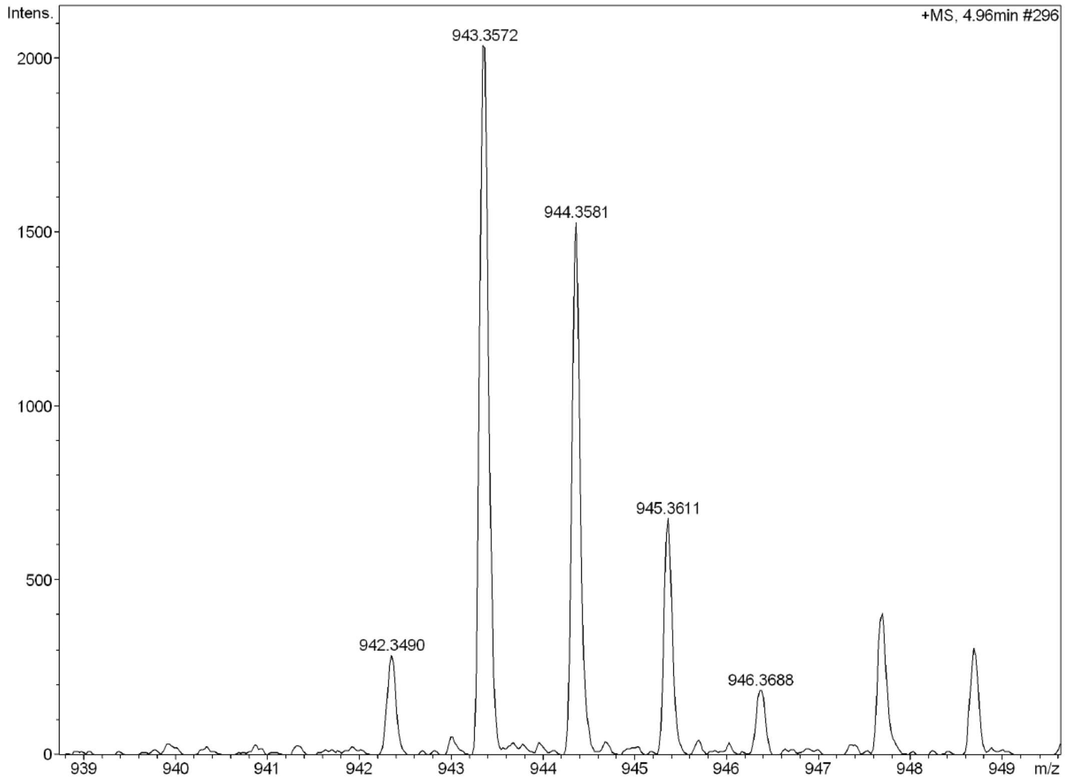

Fig. S66. HR mass spectrum (APCI) of the compound **Cor-D2**.

8. References

[1] A. Sygula, A. Sygula, L. Kobryn, *Org. Lett.* **2008**, *10*, 3927.

[2] P. Hu, S. Lee, K. H. Park, S. Das, T. S. Herng, T. P. Gonçalves, K.-W. Huang, J. Ding, D. Kim, J. Wu, *J. Org. Chem.* **2016**, *81*, 2911.

[3] F. P. Gasparro, N. H. Kolodny, *J. Chem. Educ.* **1977**, *54*, 258.

[4] *Gaussian 09; Revision A.2*; Frisch, M. J.; Trucks, G. W.; Schlegel, H. B.; Scuseria, G. E.; Robb, M. A.; Cheeseman, J. R.; Scalmani, G.; Barone, V.; Mennucci, B.; Petersson, G. A.; Nakatsuji, H.; Caricato, M.; Li, X.; Hratchian, H. P.; Izmaylov, A. F.; Bloino, J.; Zheng, G.; Sonnenberg, J. L.; Hada, M.; Ehara, M.; Toyota, K.; Fukuda, R.; Hasegawa, J.; Ishida, M.; Nakajima, T.; Honda, Y.; Kitao, O.; Nakai, H.; Vreven, T.; Montgomery, J., J. A.; Peralta, J. E.; Ogliaro, F.; Bearpark, M.; Heyd, J. J.; Brothers, E.; Kudin, K. N.; Staroverov, V. N.; Kobayashi, R.; Normand, J.; Raghavachari, K.; Rendell, A.; Burant, J. C.; Iyengar, S. S.; Tomasi, J.; Cossi, M.; Rega, N.; Millam, N. J.; Klene, M.; Knox, J. E.; Cross, J. B.; Bakken, V.; Adamo, C.; Jaramillo, J.; Gomperts, R.; Stratmann, R. E.; Yazyev, O.; Austin, A. J.; Cammi, R.; Pomelli, C.; Ochterski, J. W.; Martin, R. L.; Morokuma, K.; Zakrzewski, V. G.; Voth, G. A.; Salvador, P.; Dannenberg, J. J.; Dapprich, S.; Daniels, A. D.; Farkas, Ö.; Foresman, J. B.; Ortiz, J. V.; Cioslowski, J.; Fox, D. J.; Gaussian, Inc., Wallingford CT, **2009**.

[5] (a) Becke, A. D. *J. Chem. Phys.* **1993**, *98*, 5648. (b) Lee, C.; Yang, W.; Parr, R. G. *Phys. Rev. B: Condens. Matter* **1988**, *37*, 785. (c) Yanai, T.; Tew, D.; and Handy, N. *Chem. Phys. Lett.* **2004**, *393*, 51. (d) Ditchfield, R.; Hehre, W. J.; Pople, J. A. *J. Chem. Phys.* **1971**, *54*, 724. (e) Hehre, W. J.; Ditchfield R.; Pople, J. A. *J. Chem. Phys.* **1972**, *56*, 2257. (f) Hariharan, P. C.; Pople, J. A. *Theor. Chim. Acta* **1973**, *28*, 213.

[6] (a) S. Yamanaka, M. Okumura, M. Nakano and K. Yamaguchi, *J. Mol. Struct.* **1994**, *310*, 205. (b) K. Kamada, K. Ohta, A. Shimizu, T. Kubo, R. Kishi, H. Takahashi, E. Botek, B. Champagne and M. Nakano, *J. Phys. Chem. Lett.* **2010**, *1*, 937.

[7] Chen, Z.; Wannere, C. S.; Corminboeuf, C.; Puchta, R.; Schleyer, P. V. R. *Chem. Rev.* **2005**, *105*, 3842.

[8] Geuenich, D.; Hess, K.; Köhler, F.; Herges, R. *Chem. Rev.* **2005**, *105*, 3758.
